# Selection of reference genes for normalization of cranberry (*Vaccinium macrocarpon* Ait.) gene expression under different experimental conditions

**DOI:** 10.1371/journal.pone.0224798

**Published:** 2019-11-12

**Authors:** Chen Li, Jian Xu, Yu Deng, Haiyue Sun, Yadong Li

**Affiliations:** 1 Engineering Center of Genetic Breeding and Innovative Utilization of Small Fruits of Jilin Province, College of Horticulture, Jilin Agricultural University, Changchun, China; 2 College of Life Sciences, Jilin Agricultural University, Changchun, China; Nazarbayev University, KAZAKHSTAN

## Abstract

Real-time fluorescent quantitative PCR (qRT-PCR) is often chosen as an effective experimental method for analyzing gene expression. However, an appropriate reference gene as a standard is needed to obtain accurate gene expression data. To date, no internal reference genes have been reported for research on cranberries. Expanding the selection of internal reference genes for cranberry will enable reliable gene expression analysis, and, at the same time, can also lay a solid foundation for revealing the biological characteristics of cranberry. Here, we selected ten candidate reference gene families and used three statistical software tools—geNorm, NormFinder and BestKeeper—to evaluate their expression stability under the influence of different experimental factors. The results showed that *protein phosphatase 2A regulatory subunit* (*PP2A*) or *RNA helicase-like 8* (*RH 8*) was the best choice for an internal reference gene when analyzing different cranberry cultivars. In two sample sets comprising different cranberry organs and three abiotic stress treatments, *sand family protein* (*SAND*) was the best choice as a reference gene. In this study, we screened genes that are stably expressed under the influence of various experimental factors by qRT-PCR. Our results will guide future studies involving gene expression analysis of cranberry.

## Introduction

Cranberry (*Vaccinium macrocarpon* Ait.) is an evergreen shrub with considerable cold resistance and is often found in cool and high-altitude areas in the northern hemisphere [1]. Studies have shown that cranberries have a high nutrient content and are rich in polyphenolic bioactive substances such as flavonols, resveratrol and anthocyanidins [2]. These bioactive substances have positive effects such as preventing urinary tract infection, clearing Helicobacter pylori infection, and anti-oxidation and anti-tumor effects [2]. These advantages make it become one of the fruit crops, which have a good prospect for development. With the advancement of molecular biology research on cranberries, gene function research and transformation, as well as genetic improvement, have become hot research topics. Therefore, gene expression analysis is important for revealing the regulatory mechanisms of cranberry genes.

Gene expression analysis can help researchers to better understand genetic development mechanisms in biological research, and thus has become a common and important method. There are many methods for detecting gene expression levels. The most commonly used methods are DNA microarrays, Northern blotting, in situ hybridization and real-time fluorescent quantitative PCR (qRT-PCR). It is well known that qRT-PCR has many advantages for quantifying levels of gene transcription, and many researchers have shown that it is a fast and reliable experimental method. Its advantages include the simplicity of experimental operation and its specificity and sensitivity [3–5]. This effective tool has been widely used in many fields of research, including diagnostics, microbiology, molecular medicine, and biological sciences [3, 6, 7]. The reliability of qRT-PCR is based on high-quality template cDNA, as well as strict primer specificity, high amplification efficiency and appropriate selection of internal reference genes [8, 9]. Many researchers often use internal reference genes to compensate for differences in RNA quality, reverse transcription efficiency, and amplification efficiency among different samples, because internal reference genes can be used for data correction and standardization [10]. As a standard for gene expression analysis, an ideal internal reference gene needs to be expressed constantly regardless of experimental factors. At present, there are many commonly used internal reference genes. However, if we examine many previous studies, we find that none of these internal reference genes are stably expressed. Any reference gene that is claimed to be stably expressed is only stable within a particular cell type or a certain range of experimental factors [10]. As the demand for quantitative analysis continues to increase, researchers need to choose an appropriate gene or genes as an internal reference based on the specific experimental conditions to obtain more reliable results from gene expression analysis.

The selection of stably expressed internal reference genes under various experimental conditions is critical for the analysis of cranberry gene expression, but no detailed reports on screening internal reference genes for cranberry have been found. Based on the potential internal reference genes screened in other plant systems in previous studies, 10 candidate internal reference genes were chosen for this study. These 10 genes included *actin* (*ACTIN*), *cyclophilin* (*CYP 2*), *elongation factor-1α* (*EF-1α*), *F-box protein family* (*F-box*), *glyceraldehyde-3-phosphate dehydrogenase* (*GAPDH*), *tubulin beta* (*TUBB*), *sand family protein* (*SAND*), *18s rRNA*, *protein phosphatase 2A regulatory subunit* (*PP2A*) and *RNA helicase-like 8* (*RH 8*). We analyzed the expression levels of these candidate internal reference genes using qRT-PCR in different cranberry cultivars and organs, and in cranberry plants treated with three different abiotic stresses. Then, the experimental data were evaluated to determine the expression stability of the candidate genes using three statistical software tools, geNorm [11], NormFinder [12] and BestKeeper [13]. The stably expressed internal reference genes were screened separately under different experimental conditions. The screening of internal reference genes in this study will help researchers perform reliable gene expression analysis in cranberry, and also lay a foundation for revealing the bionomics of cranberries.

## Materials and methods

### Plant materials

We selected six different cultivars (Brewer, Bain Fav.No.1, Bain 11, Hollister Red, Bain 6, Washington) from the small berry base of Jilin Agricultural University for this study and collected young leaves from each cultivar at the same time. Different organs were collected from the same genotype (Bain 11) including roots, stems, leaves, flowers, fruits and seeds. The roots, young stems and young leaves were collected at the same age as the young leaves of different cultivars, fully open flowers were collected during the flowering period, and seeds were obtained from the harvested mature fruits. Fruit sampling included two ripening stages, the white fruit (W, 30 days after full bloom) and red fruit (R, 60 days after full bloom). The basic research material for abiotic stress treatments was cranberry (Bain 11) cutting seedlings, and we collected leaves and roots after stress treatment. All of the plant materials used in this study were frozen in liquid nitrogen and stored in a freezer at −80°C.

### Abiotic stress treatment methods

The roots of the cutting seedlings were trimmed to a length of 5.0 cm, and the leaves at the upper 1/3 of the roots were removed to fix the plants. The test cutting seedlings were immersed in a test solution of 1.0% carbendazim for 30 min and rinsed with tap water after disinfection. Then, the seedlings were transferred to a culture pot with six wells containing 4 L of nutrient solution; every well had a fixture to hold the test seedlings. One plant was placed in each well, and the wells were oxygenated throughout the day. The culture was carried out with modified Hoagland nutrient solution (pH = 5.5), and the culture solution was changed once every 3 days. Using a completely randomized experiment design, cranberry seedlings with good growth and uniform size were treated with drought stress, salt stress and alkali stress. The abiotic stress treatment schemes were: (1) salt stress treatment: 200 mmol/L NaCl; (2) alkali stress treatment: 200 mmol/L NaHCO_3_; (3) PEG simulated drought stress treatment: stress treatment was carried out with a nutrient solution supplemented with 8% PEG 8000. Untreated seedlings were used as a blank control. Leaf and roots samples were collected at five time points (0, 3, 6, 9, 12 h), with three biological replicates each. After the stresses are completed, the activity of SOD enzyme in the samples are separately detected, and the result shown in S1 File. Finally, all of the treated and control materials were frozen in liquid nitrogen and stored in a freezer at −80°C. All the above experimental materials are detailed in the S1 File.

### RNA extraction and detection

We used the improved CTAB method to extract total RNA [14]. The RNA of all extracted samples was stored in a freezer at −80°C. The purity and concentration of the RNA were analyzed with an ultra-micro UV spectrophotometer, and 1.2% agarose gel electrophoresis was used to examine the integrity of the total RNA.

### Synthesis of cDNA

In this study, we used a PrimeScript^™^ RT Reagent Kit with a gDNA Eraser-Perfect Real Time kit (Takara) for reverse transcription of sample RNA. The specific operation followed the kit instructions. The obtained cDNA was stored at −20°C.

### Selection of candidate internal reference genes and differential expression analysis

According to the results of previous studies in other plant systems [15–22], we selected 10 candidate internal reference gene families for this research. These 10 gene families included *GAPDH*, *EF*-*1α*, *ACTIN*, *TUBB*, *RH 8*, *PP2A*, *18s rRNA*, *CYP 2*, *SAND* and *F-box*. The 10 candidate internal reference gene families were screened in an annotated library consisting of 57,331 Unigenes obtained by transcriptome sequencing of cranberry fruit by Sun et al. [23]. Some Unigenes were identified for each gene family. The expression levels of all the initially screened genes were calculated through the RPKM (Reads Per kb per Million reads) method [24] using the formula:

RPKM=(10^9)×CN×L

where RPKM is the expression level of tested Unigene X, C is the number of Unigene X reads that are aligned, N is the total number of reads of all Unigenes that are aligned, and L is the total number of bases of Unigene X. The quantitative relationship of differential expression values was determined using the formula Log_2_Ratio = log_2_(R_RPKM/W_RPKM) to analyze the expression of each gene in the two libraries (red fruit and white fruit), where R_RPKM is the expression level of the gene in red fruit and W_RPKM is the expression level of the gene in white fruit. A |log_2_Ratio|≥1 indicated a significant expression difference between the libraries. A positive log_2_Ratio value indicated that the gene was significantly enriched in red fruit and a negative log_2_Ratio value indicated that the gene was significantly enriched in white fruit. Finally, we selected the gene sequence in each of the ten candidate reference gene families that had the smallest |log_2_Ratio| value (<1), which indicated that the difference in expression between red and white fruit was the least significant. Subsequent primer design was performed using this gene sequence.

### qRT-PCR

The experimental operation was completed on a StepOne^™^ Real-Time PCR System (Applied Biosystems^™^) machine. Three replicates and NTC controls were included for each sample. After optimization, the reaction system was: 10 μL TB Green Premix Ex Taq II (Tli RNaseH Plus) (2×), 0.4 μL ROX Reference Dye (50×), 0.8 μL each forward and reverse primers, 2 μL cDNA, and 6 μL sterilized water in a 20 μL total volume. The reaction conditions were a standard two-step amplification procedure for qRT-PCR; stage 1: pre-denaturation (Reps: 1, 95°C for 30 s), stage 2: PCR reaction (Reps: 40, 95°C for 5 s, 60°C for 30 s). After the amplification, the temperature was slowly increased from 50°C to 95°C, and the fluorescence intensity of the sample was continuously measured to obtain a melting curve.

### Primer design and validation

Based on the differential expression analysis of the 10 candidate reference gene families, the best gene sequence was screened from each gene family. Then, Primer Express 3.0.1 was used to design primers for the candidate internal reference genes based on the design principles for qRT-PCR primers. The design principles included the following parameters: amplicon length, about 100 bp; primer length, 18–22 bp; melting temperature (Tm), 57–61°C; GC base content, 40–60%. After primer design, the BLAST tool was used to analyze the amplicon specificity of each primer (http://blast.ncbi.nlm.nih.gov/Blast.cgi) [25]. The designed primers were synthesized by Suzhou Jinweizhi Biotechnology Co., Ltd., China.

To verify the primer amplification efficiency and create standard curves, equal amounts of cDNA template were prepared for the 10 samples. A concentration gradient was then prepared by serially diluting the cDNA templates with 5-fold dilution to give 1, 1/5, 1/25, 1/125, and 1/625 times dilutions. Three replicates were included for each reaction. The melting temperature was obtained from the software that came with the qRT-PCR machine. A standard curve was drawn from the obtained Ct values to obtain the slope k and linear correlation coefficient (regression coefficient) R^2^. The amplification efficiency E (E = 10^(−1/k)^ − 1) was calculated using the obtained slope k.

### Data analysis

We initially analyzed the expression stability of the candidate internal reference genes using box-plots [26]. A preliminary analysis of the block diagram of the quantitative cycle (Ct) values of each candidate reference gene under all experimental conditions used in this study was performed. The range of expression of each gene was calculated using the formula:

ΔCt=Ctmax‐Ctmin

Three statistical algorithms—NormFinder, geNorm and BestKeeper—were then used to further analyze the expression stability of each reference gene. geNorm [11] calculates an M value (average expression stability value) based on the Q value (calculated from 2−Δct) by stepwise removal of the most unstable genes and ranks the stability of the candidate internal reference genes by their M values. The smaller the M value, the more stable the gene, and conversely, the larger the M value, the more unstable the gene. In addition, this software recommends the optimal number of internal reference genes by pairwise variation analysis (Vn/n+1) of candidate internal reference gene normalization factors. The default threshold of Vn/n+1 is 0.15. If Vn/n+1>0.15, the combination of n genes is not very stable, and if the n+1th gene is introduced, the stability of the internal reference gene combination will be significantly improved; conversely, when Vn/n+1<0.15, the combination of n genes is sufficiently stable. When Vn/n+1 does not reach the default value, according to the geNorm manual, we can select the 2–3 most stable internal reference genes based on the V value trend. The software NormFinder [12] calculates a stability value according to the Q value (calculated from 2^−Δct^), combines the variance within each group and the variance between groups, and then evaluates the expression stability of each candidate internal reference gene according to its stability value; if a gene has a smaller stability value, it will be more stable, and conversely, the larger the value, the more unstable the gene. The software BestKeeper [13] performs pairwise correlation analysis based on the average Ct value of the 10 candidate internal reference genes in each sample. This software directly calculates the SD (standard deviation), CV (coefficient of variation) and the pairwise correlation coefficient (Poisson correlation coefficient) between each gene. The evaluation of gene stability is based on the SD values. An internal reference gene with a SD<1 is considered to be stably expressed, and the smaller the SD, the more stable the gene; conversely, the larger the SD, the more unstable the gene.

### Validation of reference genes

To validate the accuracy of the candidate reference genes, two cytochrome P450 monooxygenase (CYPs, CL1200.Contig2_All and CL3597.Contig4_All) genes were selected from our cranberry transcriptome. The primers used for analyzing expression of genes were listed in S2 File. The most stable and variable reference genes in different organs were validated using the 2^-ΔΔct^ method with three biological sample.

## Results

### RNA detection

RNA was extracted from the plant materials used in this experiment with the modified CTAB method, and all extracted RNA was tested for quality and concentration. The experimental results showed that the RNA concentration of all plant materials was above 1000 ng/μL and the 260/280 nm optical density ratios were all in the range of 1.8–2.0, indicating the RNA was of high quality. Additionally, 1.2% agarose gel electrophoresis was used to determine the integrity of the total RNA. The results (S1 Fig) indicated that the extracted total RNA was clear, and the 25S rRNA band was about twice as bright as the 18S rRNA band, there was no degradation and no visible DNA was observed. The above results indicated that the extracted total RNA met the requirements for subsequent experimental operations.

### Screening results of candidate internal reference genes

The 10 candidate internal reference gene families were screened in an annotated library of 57,331 Unigenes obtained from transcriptome sequencing of cranberry fruit, and some Unigene sequences were identified from each family. The expression differences in the obtained genes were compared and analyzed between the two libraries (red and white fruit libraries of cranberry), and the 10 candidate gene families were screened for differentially expressed genes (S1 Table). Then, the gene sequence with the smallest |log_2_Ratio| value was selected from the differentially expressed genes in each gene family. These gene sequences were used as the basis for primer design (Table 1).

**Table 1:** The genes for primer design were screened in each candidate gene family.

Gene name	Gene ID	Length	W_FPKM	R_FPKM	|log2Ratio|
***ACTIN***	CL7164.Contig7_All	649	31.4889	33.5053	0.089545964
***CYP 2***	CL4850.Contig1_All	838	738.7621	747.7516	0.017449237
***EF-1a***	Unigene457_All	486	7.4777	7.4412	0.007059297
***F-box***	Unigene5822_ALL	1024	17.5606	17.7489	0.015387477
***GAPDH***	Unigene20107_All	1383	3.9587	3.8218	0.050774458
***18S rRNA***	Unigene320_All	1490	13.6523	13.9093	0.026905794
***PP2A***	Unigene21533_All	1452	36.0478	35.7313	0.012722810
***RH 8***	Unigene16896_All	1455	23.8742	24.6959	0.048819156
***SAND***	CL5626.Contig1_All	2158	10.4761	10.3343	0.019661066
***TUBB***	Unigene2152_All	838	15.3193	15.0491	0.025673166

### Design and validation of reference gene primers

An important prerequisite for accurate evaluation of the stability of the genes chosen as candidate internal reference genes is to ensure the amplification efficiency and the specificity of the amplified product. Table 2 lists information about the candidate genes. The dissociation curves of the amplicons obtained in the experiment (S2 Table) had a single peak, and thus the specificity could be determined. The results summarized in Table 2 show that the amplification efficiency of the primers for the 10 candidate genes was 97.4–104.6%, and the linear correlation coefficient (the regression coefficient) of the standard curve (S2 Table) was 0.997–1.000 (Table 2). The above results indicated that the primers we designed met the requirements for qRT-PCR.

**Table 2:** Genes for primer design were screened in each candidate gene family.

Gene	Gene ID	Primer sequence (5’–3’)	TM (°C)	Amplicon length (bp)	Aplification efficiency (%)	Regression coefficient (R^2^)
***ACTIN***	CL7164.Contig7_All	F: ACCGTGAGAAGATGACCCAAA	81.85	120	97.4	0.999
		R: AGTCCAGCACGATTCCAGTTG				
***CYP* 2**	CL4850.Contig1_All	F: GCATACAGGCGCTGGGATA	86.46	120	102.4	0.998
		R: CTCCCGAACACCACATGCTT				
***EF-*1*a***	Unigene457_All	F: ATCCTCACTTGTGGTTGCGG	83.64	128	99.3	0.999
		R: AAGGGCATTGCTCAAAACCA				
***F-box***	Unigene5822_All	F: ACGACTATCACTCTCTGGGCTTCT	80.67	109	104.6	0.997
		R: TGTATCACTATCCCCAGCATCTGT				
***GAPDH***	Unigene20107_All	F: CTGGATAGGCTACTTGATTTGGGT	79.31	143	98.5	1.000
		R: CGGTTATTGGTACGAGGAAGTTTG				
**18*S rRNA***	Unigene320_All	F: GATGGTTTAGTCCGGATTTGCTTC	80.19	146	99.9	0.999
		R: GTATCCTCTAGTGATCCATTCTGCG				
***PP*2*A***	Unigene21533_All	F: GTTCCACATGAAGGGCCAAT	83.63	113	101.3	0.999
		R: GCTGCTATGTCCTGTCCGAAA				
***RH *8**	Unigene16896_All	F: AGTTTTCTGGTAGGGGAGACTTTC	82.29	130	102.4	0.999
		R: GACAAATGTTTACTAGAGCTGCGG				
***SAND***	CL5626.Contig1_All	F: ATGTTGTCTTCTCTTCTCTCGTCC	82.73	146	97.4	0.998
		R: AAAGAGGACACCAGAATCAGCTAC				
***TUBB***	Unigene2152_All	F: CCCTGAAGCTTTCAACACCC	87.36	113	101.7	0.998
		R: CCGAAGGTCGGAGTTGAGTT				

### Analysis of expression stability

#### Analysis of Ct values

The Ct values of the candidate internal reference genes obtained by qRT-PCR under all experimental factors were used to initially assess their expression stability. Specifically, the average Ct, coefficient of difference (CV) and standard deviation (SD) values of the candidate internal reference genes in all samples were calculated (Table 3) using the formula:

CV%=SDMeanCt

**Fig 1:**
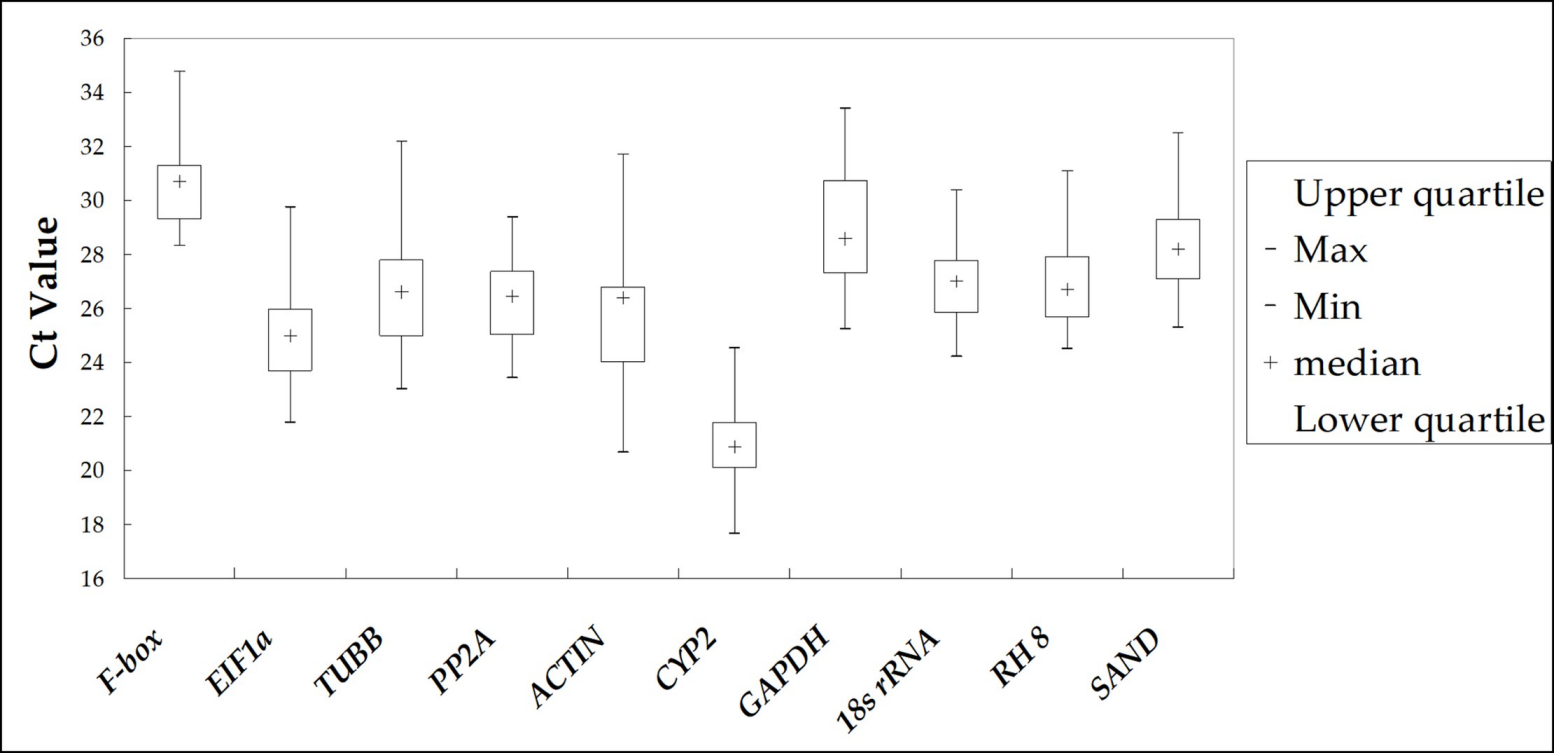
Box-plot based on cycle threshold (Ct) of 10 candidate reference genes in all experimental samples. The Ct value for each gene is the average of three biological replicates. The block diagram represents the quartile range (25th to 75th percentiles) of Ct values; the ‘+’ in the box depicts the median of the Ct value; underline and overline are determined by the minimum and maximum values of the Ct value.

Reverse transcription was performed with the same amount of total RNA from each sample, so we could assume that the range of Ct values was representative of whether the expression of the candidate gene was stable. Therefore, using the mean Ct of each experimental sample to draw a box-plot (Fig 1), we could easily and intuitively observe the expression levels of the candidate genes [26]. In this way, we initially assessed the expression stability of the 10 candidate internal reference genes in all samples under all experimental conditions (different organs, different cultivars, and different stress treatments) included in this study. All sample sets are shown in S3 Table.

**Table 3:** The value of average Ct, standard deviation (SD) and coefficient of variation (CV) were calculated from the all data for this experiment.

Gene name	Mean Ct	SD	CV(%)
***ACTIN***	25.89	2.07	8.01
***CYP 2***	20.84	1.27	6.08
***EF-1a***	24.76	1.57	6.35
***F-box***	30.56	1.48	4.84
***GAPDH***	28.96	2.12	7.31
***18S rRNA***	27.20	1.18	4.35
***PP2A***	26.37	1.44	5.47
***RH8***	27.13	1.44	5.32
***SAND***	28.42	1.54	5.41
***TUBB***	26.84	1.92	7.15

The results of this experiment showed that the Ct values of the candidate reference genes varied from 17.65 to 34.78 under all experimental conditions (Fig 1). Each candidate reference gene showed a broad expression range, which indicated that none of the candidate genes had stable expression across different sets of cranberry samples. According to Table 3, the order of Ct±SD from smallest to largest was *CYP 2* < *EF-1α* < *ACTIN* < *PP2A* < *TUBB* < *RH 8* < *18s rRNA* < *SAND* < *GAPDH* < *F-box*. The most abundantly expressed gene was *CYP 2* and its average Ct±SD (20.84±1.27) was the lowest; the average Ct±SD (30.56±1.48) of *F-box* was the highest, meaning it had the lowest expression level. The standard deviations of *18s rRNA* (SD = 1.18) and *CYP 2* (SD = 1.27) were the smallest, which indicated these two genes had the smallest variation among all the candidate internal reference genes. Regarding the CV of the Ct values, if a candidate gene has a smaller CV, it will have more stable expression. Here, the *18s rRNA* (CV = 4.35%) and *F-box* (CV = 4.84%) genes had relatively small difference coefficients, while *ACTIN* (CV = 8.01%) had the highest coefficient of variation among all test samples.

However, simply determining whether candidate internal reference gene expression is stable based on an oversimplified comparison of average Ct values is not comprehensive enough. Thus, we used three statistical algorithms for further analysis.

#### GeNorm analysis

The stability values (denoted as M) of the candidate reference genes were calculated by the software geNorm to define their order. The M value of a gene is negatively correlated with its stability, and 1.5 is used as the cutoff level of the M value. Specifically, if the M value of a candidate gene is less than 1.5, the gene can be chosen as an internal reference gene; otherwise, it should not be selected as an internal reference gene. In this experiment, the M values for all experimental materials were below 1.5, meaning the most suitable gene among them for use as an internal reference could be determined.

**Fig 2:**
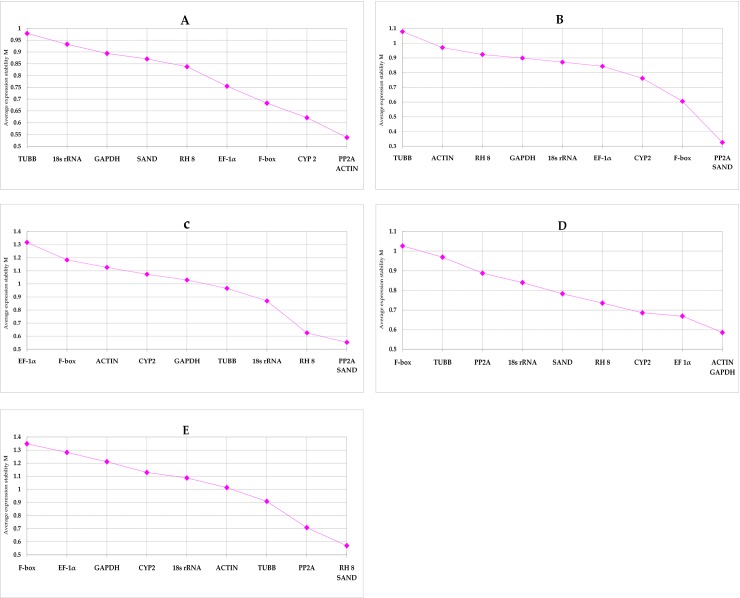
The average expression stability value (M) of the candidate reference genes in the five sample sets obtained by geNorm. (A) different cultivars; (B) different organs of the same genotype; (C) leaves treated by three abiotic stresses; (D) roots treated by three abiotic stresses; (E) ‘leaves + roots’ treated by three abiotic stresses.

Our research showed that in the two sample sets of different cultivars (Fig 2A) and different organs (Fig 2B), the most stably expressed candidate internal reference genes (with the lowest stability values, M) were *PP2A*/*ACTIN* (M = 0.54) and *PP2A*/*SAND* (M = 0.33), while the most unstable candidate internal reference gene (with the highest stability values) was *TUBB*, with expression stability values (M) of 0.98 and 1.08, respectively. Additionally, in the three different sample collections of leaves (Fig 2C), roots (Fig 2D) and ‘leaves + roots’ (Fig 2E) treated with three abiotic stresses, the most stable candidate internal reference genes were *PP2A*/*SAND* (M = 0.55), *ACTIN*/*GAPDH* (M = 0.59), and *RH 8*/*SAND* (M = 0.57), respectively. Furthermore, in the stress-treated leaf sample set, the most unstable candidate internal reference gene was *EF-1α* (M = 1.32). Among stress-treated root samples and the combined root and leaf samples, the most unstable candidate internal reference gene was *F-box*, with M values of 1.03 and 1.35, respectively.

**Fig 3:**
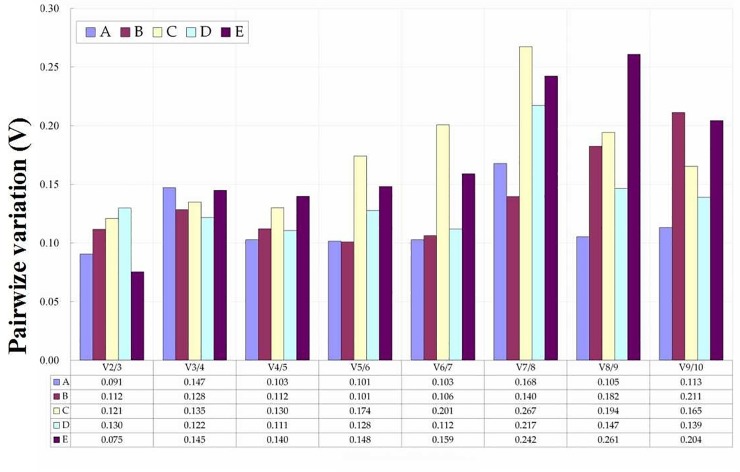
Pair-wise variation (Vn/n+1) calculated by geNorm to determine the minimum number of reference genes for accurate normalization in different experiment conditions. (A) different cultivars; (B) different organs of the same genotype; (C) leaves treated by three abiotic stresses; (D) roots treated by three abiotic stresses; (E) ‘leaves + roots’ treated by three abiotic stresses.

In addition, according to the pairwise coefficient of variation (Vn/n+1) obtained from geNorm, we determined the most appropriate number of reference genes for each set of experimental conditions. In this study, we performed pairwise variation analysis on five sample sets (A: different cultivars; B: different organs; and stress-treated C: leaves; D: roots; E: ‘roots + leaves’). As shown in Fig 3, pairwise variation analysis showed that only two reference genes was required for each sample set for normalization, as the V2/3 values of all sample sets were below 0.15, which is recognized as a threshold. Tables 4–6 summarize the best combinations for all sample sets according to geNorm.

**Table 4:** Rankings of candidate reference 10 candidate reference genes in different cultivars samples and different organs samples in cranberry using the geNorm, NormFinder and BestKeeper algorithms.

Gene	Different cultivars samples in cranberry								Different organs samples in cranberry								
	geNorm		NormFinder		Bestkeeper			Com.	geNorm		NormFinder		Bestkeeper			Com.	
	M	Rank	S	Rank	SD	CV(%)	Rank	Rank	M	Rank	S	Rank	SD	CV(%)	Rank		Rank
***ACTIN***	0.857	1	0.200	4	0.616	2.661	6	3	1.199	9	0.651	9	1.789	7.290	10		10
***CYP 2***	0.949	6	0.340	7	0.629	3.108	8	8	0.921	2	0.342	2	0.989	4.946	4		2
***EF-1a***	0.958	7	0.200	5	0.439	1.836	2	4	1.088	6	0.571	8	0.900	3.626	2		6
***F-box***	0.891	2	0.386	8	0.656	2.188	6	6	1.092	8	0.559	7	1.470	4.790	5		8
***GAPDH***	1.085	8	0.136	3	0.574	2.180	4	5	0.979	3	0.389	3	1.244	4.374	3		3
***18S rRNA***	1.094	9	0.322	6	0.689	2.634	8	9	1.046	5	0.507	5	0.781	3.001	1		5
***PP2A***	0.910	3	0.132	2	0.455	1.805	2	1	0.852	1	0.228	1	1.579	6.108	8		4
***RH8***	0.943	5	0.105	1	0.305	1.204	1	1	1.091	7	0.550	6	1.378	5.127	6		7
***SAND***	0.940	4	0.388	9	0.617	2.340	5	7	1.006	4	0.473	4	1.708	6.201	9		1
***TUBB***	1.163	10	0.417	10	0.974	4.031	10	10	1.513	10	0.954	10	1.495	5.637	7		9
**Best gene**	*ACTIN*		*RH 8*		*RH8*			*PP2A/* *RH 8*	*PP2A*		*PP2A*		*18s rRNA*			*SAND*	
**Worst gene**	*TUBB*		*TUBB*		*TUBB*			*TUBB*	*TUBB*		*TUBB*		*ACTIN*			*ACTIN*	
**Best combination**	*PP2A & ACTIN* (0.091)								*PP2A & SAND*(0.112)								

M: Stability value determined by geNorm analysis. A lower M value indicates higher expression stability; S: Stability value determined by NormFinder analysis. A lower S value indicates higher expression stability; SD: Standard Deviation calculated by BestKeeper analysis. A lower SD value indicates higher expression stability; CV (%): Coefficient of Variation calculated by BestKeeper analysis. A lower CV value indicates higher expression stability; Com.: Comprehensive ranking, which corresponds to the geometric mean of ranks determined by the geNorm, NormFinder, and BestKeeper algorithms.

**Table 5:** Rankings of candidate reference 10 candidate reference genes in leaf or root tissue samples treated with three stresses, using the geNorm, NormFinder and BestKeeper algorithms.

Gene	Leaf tissue samples in cranberry								Root tissue samples in cranberry							
	geNorm		NormFinder		Bestkeeper			Com.	geNorm		NormFinder		Bestkeeper			Com.
	M	Rank	S	Rank	SD	CV(%)	Rank	Rank	M	Rank	S	Rank	SD	CV(%)	Rank	Rank
***ACTIN***	1.419	8	0.405	9	1.525	5.795	10	9	0.986	6	0.276	5	0.808	3.007	5	6
***CYP 2***	1.213	4	0.326	6	0.871	4.231	3	3	0.957	4	0.279	6	0.628	2.888	3	5
***EF-1****α*	1.852	10	0.564	10	1.457	6.015	9	10	0.919	3	0.258	4	0.611	2.377	1	1
***F-box***	1.431	9	0.394	8	0.899	2.880	1	7	1.254	10	0.409	10	1.229	4.059	9	10
***GAPDH***	1.237	5	0.323	4	1.153	4.059	5	5	0.957	5	0.223	2	0.874	2.840	4	3
***18s rRNA***	1.187	3	0.287	1	0.983	3.631	2	1	1.215	9	0.389	9	0.769	2.781	2	7
***PP2A***	1.176	2	0.324	5	1.140	4.273	6	3	1.057	7	0.347	7	1.016	3.789	8	8
***RH 8***	1.299	7	0.374	7	1.317	4.766	7	8	0.840	1	0.192	1	0.881	3.236	6	1
***SAND***	1.112	1	0.287	2	1.135	3.935	4	2	0.885	2	0.234	3	0.971	3.363	7	4
***TUBB***	1.241	6	0.307	3	1.439	5.355	8	6	1.195	8	0.379	8	1.385	5.008	10	9
**Best gene**	*SAND*		*18s rRNA*		*F-box*			*18s rRNA*	*RH 8*		*RH 8*		*EF-1α*			*EF-1α*/ *RH 8*
**Worst gene**	*EF-1α*		*EF-1α*		*ACTIN*			*EF-1α*	***F-box***		***F-box***		*TUBB*			*F-box*
**Best combination**	*PP2A & SAND* (0.121)		*PP2A & CYP2* (0.190)						*ACTIN & GAPDH* (0.130)		*EF-1α & RH 8* (0.107)					

M: Stability value determined by geNorm analysis. A lower M value indicates higher expression stability; S: Stability value determined by NormFinder analysis. A lower S value indicates higher expression stability; SD: Standard Deviation calculated by BestKeeper analysis. A lower SD value indicates higher expression stability; CV (%): Coefficient of Variation calculated by BestKeeper analysis. A lower CV value indicates higher expression stability; Com.: Comprehensive ranking, which corresponds to the geometric mean of ranks determined by the geNorm, NormFinder and BestKeeper algorithms.

**Table 6:** Rankings of candidate reference 10 candidate reference genes in Total (‘leaves+roots’) samples treated by three stresses, using the geNorm, NormFinder and BestKeeper algorithms.

Gene	Total (‘leaves+roots’) samples in cranberry							
	geNorm		NormFinder		Bestkeeper			Com.
	M	Rank	S	Rank	SD	CV (%)	Rank	Rank
***ACTIN***	1.315	6	0.430	4	1.154	4.340	7	6
***CYP 2***	1.223	2	0.392	1	0.976	4.611	3	2
***EF-1a***	1.584	9	0.590	8	1.366	5.473	8	8
***F-box***	1.611	10	0.700	9	1.179	3.835	6	8
***GAPDH***	1.506	8	0.743	10	1.642	5.549	10	10
***18S rRNA***	1.294	5	0.425	3	0.892	3.260	1	3
***PP2A***	1.227	3	0.450	6	1.092	4.084	3	4
***RH8***	1.271	4	0.514	7	1.099	4.006	3	5
***SAND***	1.130	1	0.396	2	1.052	3.645	2	1
***TUBB***	1.319	7	0.446	5	1.406	5.158	8	7
**Best gene**	*SAND*		*CYP 2*		*F-box*			*SAND*
**Worst gene**	*F-box*		*GAPDH*		*ACTIN*			*GAPDH*
**Best combination**	*RH 8 & SAND* (0.075)		*PP2A & CYP2* (0.213)					

#### NormFinder analysis

NormFinder calculates a stability value (‘S’) based on the Q value (calculated from 2^–Δct^), combining the variance within a group and the variance between groups. We ranked the expression stability of all candidate internal reference genes in different sample sets, the gene with the lowest ‘S’ having the most stable expression level. The S values and rankings for every candidate internal reference gene in the five sample sets are summarized in Tables 4–6, and the specific information is given in S4 Table (NormFinder).

As shown in the analysis in Table 4, among the two sample sets of different cultivars and different organs, the most stable genes were *RH 8* and *PP2A*, respectively, and the most unstable candidate internal reference gene was *TUBB*. Additionally, Table 5 shows that the candidate internal reference genes with the most stable expression in the two sample sets of cranberry leaves and roots treated with three different abiotic stresses were *18s rRNA* and *RH 8*, and that *EF-1α* and *F-box* had the most unstable expression in these two sample sets, respectively. However, Table 6 shows that *CYP 2* and *GAPDH* had the most stable and unstable expression in the sample set ‘roots+leaves’, respectively.

NormFinder uses a reliable statistical framework to estimate the overall variability of candidate gene expression and the differences between test sample subgroups [12]. According to the intra-group and inter-group variation of the leaf sample sets (3 groups), root sample sets (3 groups) and root and leaf combined sample sets (6 groups) exposed to different abiotic stresses (Tables 5 and 6), as calculated by NormFinder, we concluded that *PP2A*+*CYP 2* and *EF-1α*+*RH 8* were the best combinations for data normalization in the leaf and root sample sets, respectively. Additionally, *PP2A*+*CYP 2* was selected as the best combination in the sample set ‘roots+leaves’.

#### BestKeeper analysis

BestKeeper can evaluate expression stability directly using the Ct values of the internal reference genes. We used the BestKeeper algorithm to analyze the candidate genes and found that *RH 8* and *18s rRNA* showed excellent stability when used for data normalization in two sample sets (different cultivars and different organs), whereas *TUBB* and *ACTIN* showed the lowest expression stability (Table 4). In the two sample sets (Table 5) of leaves and roots treated with three abiotic stresses, the genes *EF-1α* and *F-box* had the most stable expression, respectively, while the most unstable genes were *ACTIN* and *TUBB*. *F-box* showed outstanding performance in the sample set of leaves and roots combined (Table 6), while the most unstable gene in this set was *ACTIN*.

#### Comprehensive analysis of data

Comparing all the data (Tables 4–6), similar rankings of expression stability under different experimental conditions were obtained for the candidate internal reference genes with all three statistical algorithms. Finally, we used the geometric mean of the rankings obtained by the three statistical algorithms to calculate the consensus ranking for every candidate reference gene (Tables 4–6). Based on our comprehensive assessment, *PP2A* and *RH 8* exhibited the same level of stability and were identified as the most stable candidate internal reference genes in the sample set of different genotypes, thus these two genes were simultaneously identified as the best choice for an internal reference gene in this sample set; at the same time, *TUBB* was considered the most unstable gene in this sample set. *SAND* had the most stable expression in the different organ sample set, and *ACTIN* was the most unstable gene. From Table 6, we concluded that under the three abiotic stress treatments, the candidate internal reference gene with the highest stability in roots and leaves was *SAND*, and the least stable gene was *GAPDH*.

As shown in Tables 4–6, geNorm and NormFinder gave recommendations for the best combination of internal reference genes under the influence of different experimental factors according to their own algorithms. For the sample sets of different cultivars and different organs, ‘*PP2A*+*ACTIN’* (geNorm) and ‘*PP2A*+*SAND’* (geNorm) were chosen as the optimal combinations of internal reference genes, respectively. Among the sample sets treated with three abiotic stresses, the best combination of internal reference genes for the leaf sample set was ‘*PP2A*+*SAND’* (geNorm) and ‘*CYP 2*+*PP2A’* (NormFinder); for the root sample set, ‘*GAPDH*+*ACTIN’* (geNorm) and ‘*EF-1α*+*RH 8*’ (NormFinder) were the best combinations of internal reference genes; and ‘*RH 8*+*SAND’* (geNorm) and ‘*PP2A*+*CYP 2*’ (NormFinder) were the best combinations of internal reference genes in the ‘roots+leaves’ sample set.

#### Validaion of the stability of selected reference genes

**Fig 4:**
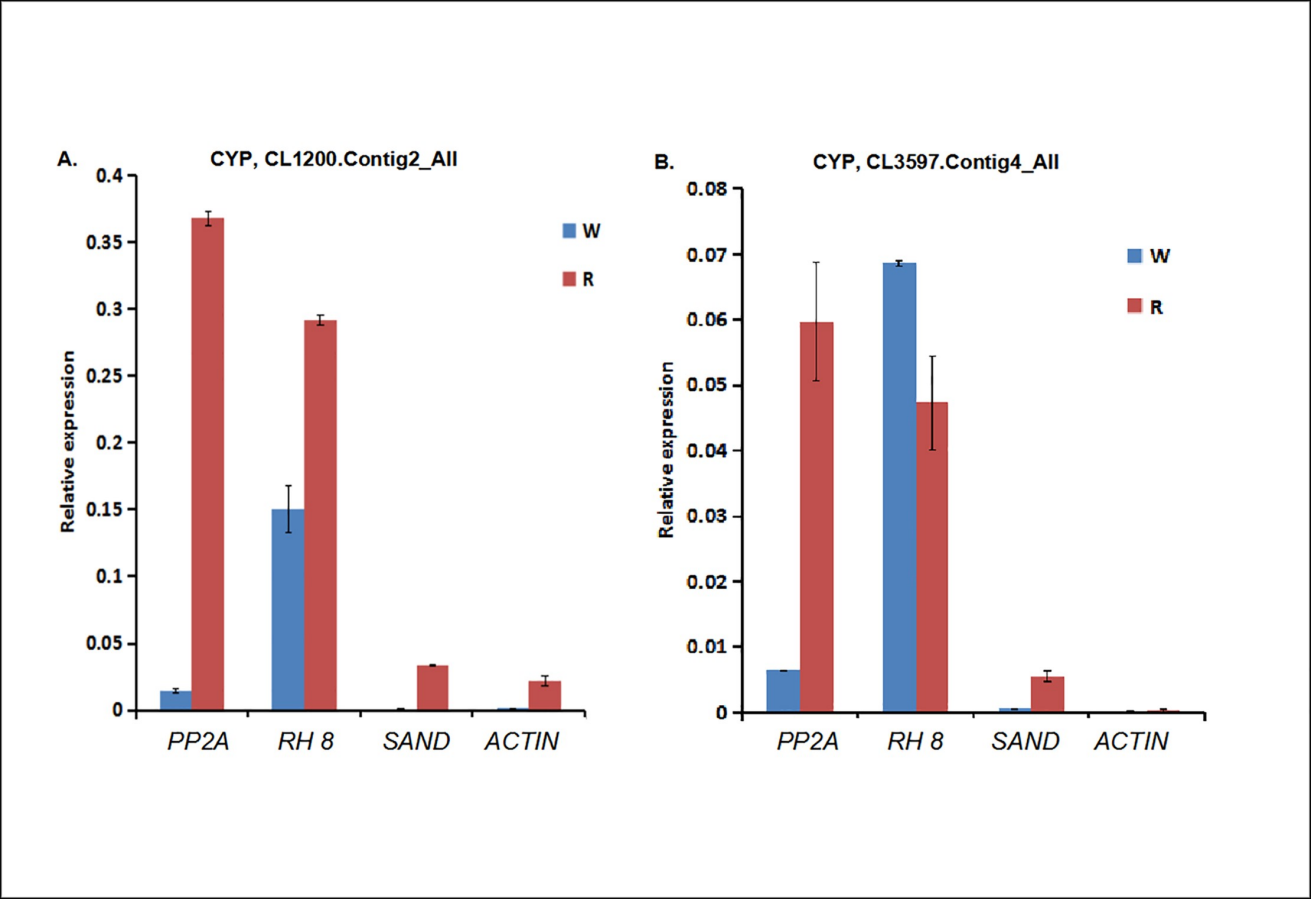
Relative expression profiles of CYPs in between white (W) and red (R) fruits in cranberry normalized by *PP2A*, *RH 8*, *SAND* and *ACTIN*, respectively. CL1200.Contig2_All; (B) CL3597.Contig4_All.

To determine the accuracy and reliability of candidated reference genes, the expression of CYPs was calculated with the four candidate reference genes selected. CYPs play significant roles in a wide range of secondary metabolite biosynthetic reactions. Our transcriptome data showed that the expression of CYPs were up-regulated in the red fruit stage [23]. When the most stable reference gene in cranberry, *PP2A* and *SAND*, were used for normalization, the transcript levels of CYPs were consistent with our digital gene expression. By contrast, *RH 8* and *ACTIN* were used, the expresion patterns were different (Fig 4).

## Discussion

Analysis of gene expression under different experimental conditions is a major aspect of gene function analysis. qRT-PCR technology has the advantages of high specificity, high sensitivity, good reproducibility, speed and efficiency, and can provide accurate results for gene expression analysis. In recent years, it has been widely used by many researchers to analyze gene expression. However, qRT-PCR requires a stably expressed gene as an internal reference to obtain accurate results for gene expression analysis. Currently, no known reference gene has stable expression under all experimental conditions. Therefore, to ensure the accuracy and reliability of qRT-PCR data correction and normalization, it is necessary to screen internal reference genes for specific experimental factors.

Here, ten candidate internal reference genes were selected for study and the expression stability of these genes in five cranberry sample sets (different cultivars, different organs, and leaves, roots, and ‘leaves+roots’ treated with three abiotic stresses) was analyzed using three statistical algorithms. The results showed that the expression stability of the ten candidate internal reference genes differed in the five sample sets. Thus, the three software tools gave the best choice of internal reference genes for different experimental factors. At the same time, we identified the least stable internal reference genes based on data analysis.

From the results this study, we can conclude that the appropriate internal reference genes for cranberry change depending on experimental factors. This result strongly confirms that no genes are stably expressed under all experimental conditions. Therefore, the screening of suitable internal reference genes in cranberry for specific experimental factors in this study is of great value. Furthermore, although the reliability of the experimental results can be improved using an appropriate combination of internal reference genes, it is time consuming and expensive to perform qRT-PCR experiments. Therefore, we recommend that the accuracy and cost of the reference combination be fully considered when selecting internal reference genes.

The experimental data obtained from this study were compared with experimental results obtained from various tissue types in other plant species, and the results showed a degree of consistency with those from plants such as blueberry [27], *Arabidopsis* [15] and tomato [28]. However, the reference genes that were stably expressed in other plant systems were found to have poor expression stability in cranberry. For example, our results indicated that *EF-1α* had the lowest expression stability in the leaf sample collection from cranberry (Bain 11) plants treated with three different abiotic stresses. However, in previous studies, *EF-1α* was one of the most stably expressed genes in cucumber [29] and grape [16]. In addition, *GAPDH* exhibited stable expression in a variety of tissue types in grape and sugarcane [16, 17]; however, in this study, it showed the lowest stability in the combined leaf and root sample collection from cranberry (Bain 11) plants treated with three different abiotic stresses. These results further demonstrate the need for assessment of internal reference genes in specific plant systems.

According to previous studies involving qRT-PCR experiments in other species or plants treated under various experimental conditions, the expression of traditional genes selected as references is not always stable. The results of this study show that among the genes often selected as references, such as *GAPDH*, *TUBB*, *EF-1α*, *18s rRNA* and *ACTIN*, only *18s rRNA* showed high stability in cranberry and only in the leaf sample set treated with three different abiotic stresses, while the rest showed relatively low stability in cranberry under different experimental conditions. This finding is consistent with previous studies in blueberry [27] and *Arabidopsis* [15]. Specifically, some traditional internal reference genes such as *EF-1α*, *GAPDH*, *TUBB* and *ACTIN* were found to have relatively low expression stability in different varieties and in rabbit blueberries treated with abscisic acid. The stability of *ACTIN* and *EF-1α* expression was also observed to be low in a study of several tissue types of *Arabidopsis*. We were pleasantly surprised to find that non-traditional internal reference genes such as *PP2A*, *RH 8* and *SAND* showed the highest expression stability in different cranberry cultivars. The results of this study are relatively consistent with the results of previous studies on blueberry [27] and papaya [30]. Specifically, in previous studies, *PP2A* was the most stably expressed gene and another new internal reference gene, *RH 8*, was ranked second in blueberry under the influence of specific experimental factors, and the *SAND* gene had the most stable expression in papaya (different tissues).

## Conclusions

In summary, we assessed the expression stability of 10 candidate internal reference genes for data normalization in five sample sets: different cranberry cultivars, different organs of the same genotype, and leaves, roots, and leaves + roots under three abiotic stress treatments. When the sample sets treated with the three abiotic stresses were considered together, *SAND* was identified as the best choice for an internal reference gene; however, when considering leaves and roots treated with the three abiotic stresses separately, the *18s rRNA* and *RH 8* or *EF-1α* genes were the best choices for internal reference genes, respectively. Among different cultivars, *PP2A* or *RH 8* was recommended as the best choice for an internal reference gene. In different organs, the expression stability of the *SAND* gene was the highest.

## Supporting information

S1 Fig Agarose gel electrophoresis for total RNA of partial samples. (TIF)

S1 File Effect of three abiotic stresses on SOD activity of cranberry. (DOC)

S2 File The primer pairs location on transcript sequence. (DOC)

S1 Table Differentially expressed gene sequences selected from 10 candidate gene families. (DOC)

S2 Table Primer pair amplification specificities for qRT-PCR. (DOC)

S3 Table Description of the samples used for qRT-PCR, and the 5 different combinations of sample sets. (DOC)

S4 Table NormFinder analysis. (DOC)
