# Supplementary figures and images for: Selection of reference genes for normalization of cranberry (Vaccinium macrocarpon Ait.) gene expression under different experimental conditions
Source: PLoS One. 2019 Nov 12;14(11):e0224798. doi: 10.1371/journal.pone.0224798 (PMC6850891; doi:10.1371/journal.pone.0224798)

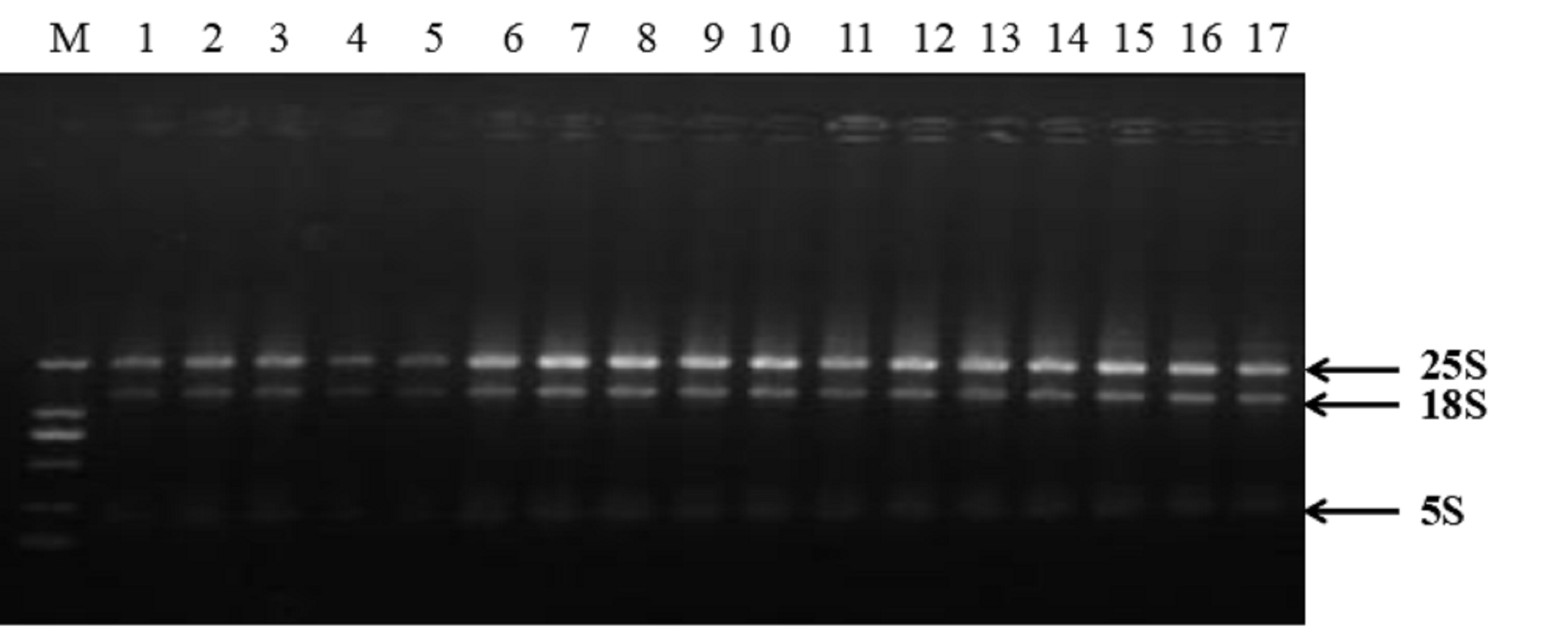

Supplement: S1 Fig — (TIF) [file pone.0224798.s001.tif]

**S2 File. Effect of three abiotic stresses on SOD activity of cranberry.**


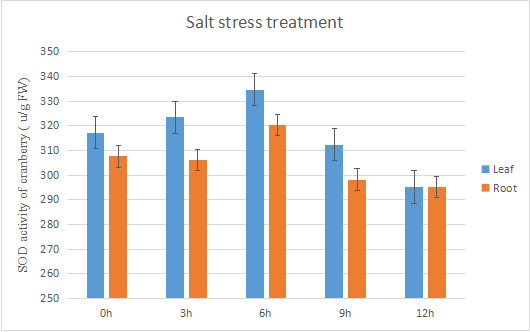


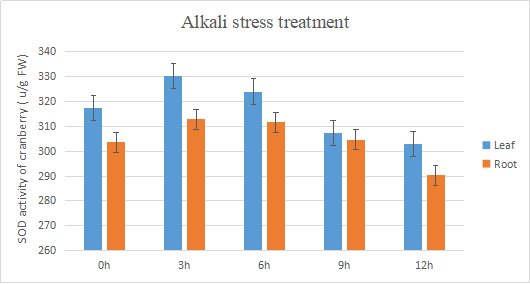


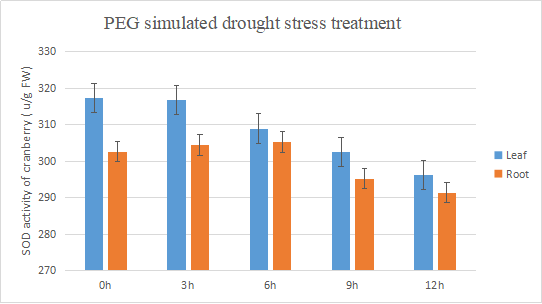

Supplement: S1 File — (DOC) [file pone.0224798.s002.doc]
