# Supplementary material for: Selection of reference genes for normalization of cranberry (Vaccinium macrocarpon Ait.) gene expression under different experimental conditions
Source: PLoS One. 2019 Nov 12;14(11):e0224798. doi: 10.1371/journal.pone.0224798 (PMC6850891; doi:10.1371/journal.pone.0224798)
Supplement: S2 File — (DOC) [file pone.0224798.s003.doc]

**S1 File The primer pairs location on transcript sequence.**

Primer sequences were in red boxes.

CL7164.Contig7_All *Actin*

GCATCACACATTCTACAATGAGCTCCGAGTTGCCCCAGAGGAGCACCCTGTCCTCCTTACTGAAGCACCTCTGAACCCCAAGGCTAACCGTGAGAAGATGACCCAAATCATGTTTGAAACCTTCAACACCCCTGCTATGTATGTTGCTATTCAGGCTGTGCTTTCCCTATATGCTAGTGGTCGTACAACTGGAATCGTGCTGGACTCTGGCGATGGTGTGAGCCATACGGTCCCTATATATGAAGGATACGCACTCCCACACGCCATCCTTCGTCTTGACTTGGCAGGCCGCGATCTCACAGATGCTTTGATGAAAATCCTTACTGAGCGTGGCTACTCCTTCACCACTACAGCAGAGCGGGAAATCGTAAGGGACATGAAGGAGAAGCTCGCTTACATTGCCCTAGACTATGAGCAAGAGCTAGAGACTTCCAAAACAAGCTCCTCAGTTGAGAAAAGCTATGAGCTTCCCGATGGCCAAGTGATCACCATTGGCGCAGAGCGTTTCCGGTGCCCCGAGGTTCTTTTCCAGCCGTCGATGATTGGAATGGAAGCGGCGGGAATTCACGAGACCACTTATAATTCTATCATGAAGTGTGATGTGGATATCAGAAAAGATCTCTACGGAAACATTGTGCTCAGTGGTGGT

CL4850.Contig1_All *CYP 2*

AATCGCTTGAAACCTAAGCAGTGTACAAGTCAACTTCACAGTGTGAGTGTTTCTGTGTGAGAGAGAGAGAGTGAAGAGAGAGAATGGCAAACCCTAAGGTTTTCTTCGACATGACCGTCGGCGGCGCACCGGGTGGCCGGATCGTGATGGAGCTCTACGCCGACACCACCCCTCGCACGGCGGAGAACTTCAGGGCACTTTGCACAGGAGAGAAAGGCGTCGGGAAGAGCGGCAAGCCTTTGCACTTCAAGGGCTCCTCCTTCCACCGCGTGATCCCGGGGTTCATGTGCCAGGGAGGCGACTTCACCGCCGGAAACGGAACCGGAGGCGAGTCGATTTACGGATCGAAGTTTCCCGATGAGAACTTCGAGAAGAAGCATACAGGCGCTGGGATACTGTCGATGGCGAATGCTGGGCCCAACACCAACGGATCGCAGTTTTTCATCTGCACGGCTAAGACCGATTGGCTCGACGGGAAGCATGTGGTGTTCGGGAGAGTTGTGGAAGGGATGGATGTTGTGAAGGCGATCGAGAAGGTTGGATCGAGCTCCGGGAGGTGCTCGAAGGTTGTGACGATTGCTGATTCCGGTCAGCTCTCTTGAGTTTTCACTTATGTTGAATAATTTGATCTGATGAGATCTATTTGAGATGCTTCGTCTGTTATCTATCTAGGCTTGTGGTTTTTGTTGAGTCTGGTGGTTATGATCTAGACCTTGGTTCTGGTTTGGTTACTTTGAATGGAAGACTCTTCCTGTCTTTAATTACTGCTATGGAAATAAAATAGTTAATGTTTACTTATTACGTCTTTTTTTTTTGACTTATTACGTTTTTGGGGCAA

Unigene457_All EF-1α

TTTTGCCGTGAGGGACATGCGGCAAACGGTGGCCGTTGGTGTCATCAAGAGTGTCGAGAAGAAGGATCCAAGTGGGGCCAAGGTCACCAAGGCTGCTCTGAAGAAGAAATGAATGGTGGCCCGCAATGAGGTTTTGATACTTTGGAACAAAACTGAAGTTTTTTAATGCTGTTTTATTAGGTTTTGCAAGTTTTTTGTTGAATGTTTGGTTGGACATTTGTTGCTGTTCACTATCCTCACTTGTGGTTGCGGGGACGGTGGTGGTGTTAAGCATTTAGGTGCCATATCTGGGTGCTTGACAGGCTGCGGCAAATGGTTTCCTTTTTTTTTACTCTCTTTTTGGTTTTGAGCAATGCCCTTAGCATTCTTCTTCATATTGTTTTAATTTTCATTTTAAGTATATGAATTGTTCCATTCGGATACTCGGTACTTCTTCGTTTTTTTAAAATTCCATTTGAAGTATATGAATTGCTACATTCGGATGCC

Unigene5822_All *F-box*

GATTTGCCAATTTTGTTAAACACTGCCGTAAACTTCAGCGTCTATGGATTACAGGCTCTATTGGAGACGGGGGGCTATTAATTGTATCTTCTACTTGTAAGGATCTGCGTGATTTGAGGCTATTTAAGCCTAATCCTGCTGTGGTGAATGGAGGTGCAACTGAAGAGGGTTTGGTTGCTATATCGAAGGGCTGCCCACTGCTTCACTCTTTGGAATTCTTCTGCCACCGGATGACGAATGCTGCCTTAATAACTCTATCTAAGAACTGCCCAAATATCACTTCCTTCAGTTTATGTATCCGCGAGTCAAAGAAACCTGATCATGTCACTTTGCTGCCACTGGATGAAGGTTTTGGGGCTATTGTACAATATTGCTCAGGGTTGAGACGACTATCACTCTCTGGGCTTCTAACGGACAGAGTTTTTCTTTATATTGGAATGTACGGGGAGAAATTGGAGGTGCTTTCGGCCACAGATGCTGGGGATAGTGATACAGGAATGGTTTATGTCGTGAACGGATGTAGAAACCTTAGAAAATTGGAGTTCACCGATTGTCCCTTTGGCGATACTGCACTGCTTGCTAATGTCGAAAGGTACGAAACGATGCGATACCTCTATATGTCCTGGTGTCAAGTTACGTTGGGAGGTTGCAAGGTTCTAGCAAAGAAAGTGCCATCGCTGAATGTAGAGGTGATAAATGGACAGGAAGAAGTCGAGGATTATCCTGATGATAGATTGAGGGTGAAGAAGTTGTATGTTTACCGGACCCTTCATGGACCACGGGCTGATGCACCTGAGCACGTCTGGACTTTATAGGTAGGAGTTATTTCAGGCATTTTTGTGTTTTGAGGGAGGTATTTTGAATCATGTTTTTGCATGGGTTTCAAATTAGTGATGAAGAAGTTGGATATTGTGCATATATCATTGGTCTTGAGCTTTGAAAAACATATATTTAGTTTCTGATGTAAATAAATACGAGTGCAAGGGATTCTGGTTTGAATACTAGAATAAGAAGTTGATTACCA

Unigene20107_All GAPDH

TCATATTTATATATACTCCGCACAAATCGTACGAGCGGTGCGTACCATATCTCCCACCTCTGAAATATTGTATGATAAAGAAAGAACAGCATTCCGAGTTGGATGGGTGAGTCAAATGAAGATTTTTGTGATCAAGACAAGAAGTCTCCTAGTTCAAATTCATTTGGGTTTGTTTCTTGCACTACTTTCAATATTCTTGCTCCAATTTACAAGCGACTGAACGACCAGAACTGTGAGAGTGAGTTCCGTGAACTTTGGCTTAGCAGGAATCAGAGCATACTGGATAGGCTACTTGATTTGGGTTCCTCAATTTTATGTTTACAGGAATTTTGGGTGCAAAATGAAGAACTTGTGAGGATGTATGAGAAGAGACTTGGAGATGCTGGCTATCAGACTTACAAACTTCCTCGTACCAATAACCGGGGAGATGGTCTTCTTACAGCAGTTCTCCGGACCCACTTCCATGTTTTGAATCACCAAGAATTTCGATTTAATGATATCGGTGATCGTGTAGCCCAGCTTCTACATGTTGAGTTACTTGATCATTCTTCTCATAACCAACCCACTAAGATGGAGAAGCAAGTTCTTGTTGTCAATACTCACTTAATTTTCCCGCATGATTCGAGTTATTGCTTTGTCAGGTTGCAACAGGTATACAAGATTTTGCAGTACATAGATCAATACTATAAAGAAAACCAGCTCCCACCTCTTCCGGTAATTTTATGCGGGGATTGGAATGGAAGTAAGAAAGGGAATGTATATAAGTTTCTCCAATCACAAGGTTTTGTCTCATCATATGATATTGCTCCCCCACATACCAATTGTGGAGAAGATTTCTTTGAGTGGATTAGCCATCGTAACCACAGAGGAAATGTATGTGGAGTGGATTTTATTTTGCTTCGCAATCCCAGCAAACATCGGAAGCCACTAAAAGAAAGCCTGATGGAAGCAGTTCTTGGGAACATAAATAACCTGTCACTCAAATTGTCAACAGAAGCCATTGGTCCATTACACTTTCTTGAAACTGATGGGAGTCACATAAACTATTCTCAGTTTTCACAAGCTTTAGCTAAGTTGGGCCTAAGTGGTCATCCTGAAGATTGTCTTGGTACTGAAGACATAAAAGAGTTGTGGGAACATATCGACACAGATGAGGATGGTGTGATTCATATATCAGACTTCTCTAAGGCAAGAAACTCGTCTGGTTTCGCGCGGCAGAATGAAGACAATGAAGAAAATACTGCCACCGCAATTCATTTCAAGGTGACCAAAGCGACGTTGTTTCCTGCAGAAGTTGAAAGAGGGATGTGGCCTGAGGATTATTCCCTATCCGATCATGCCCAGTTAACTGTAGTGCTTTCCCCAGTGCAGTAACACAGTTCT

Unigene21533_All *PP2A*

AGAGAGAGAGAGAGAGATAGAGAGGAGAGAGAGAATGCCTACGCAGGGGGATCTGGATCGGCAGATCGAGCACTTGATGGAGTGCAAGCCTTTGACGGAAGCAGAGGTGAAGACGCTGTGCGATCAAGCTAGGGCTATTCTGGTGGAGGAATGGAATGTACAGCCAGTCAAGTGTCCTGTTACGGTCTGTGGTGATATTCACGGACAGTTTTACGATCTGATCGAGCTGTTTCGGATAGGCGGAAATGCGCCCGATACTAATTATCTTTTCATGGGGGATTATGTTGATCGTGGGTACTATTCAGTGGAGACTGTCACACTTTTAGTGGCTCTGAAAGTTCGTTATAGGGATAGAATTACAATCCTTAGAGGAAATCATGAGAGCCGGCAAATTACACAAGTGTATGGTTTCTATGATGAATGCTTGAGGAAGTATGGAAATGCCAATGTCTGGAAGTATTTTACTGACCTTTTTGATTATCTGCCCCTTACAGCTCTAATTGAGAGTCAGATTTTCTGTTTGCATGGTGGACTTTCCCCATCCTTGGATACACTAGACAACATCCGAGCCTTGGACCGTATACAGGAGGTTCCACATGAAGGGCCAATGTGTGATCTGTTGTGGTCTGATCCAGACGATCGATGTGGGTGGGGAATATCTCCACGCGGAGCTGGTTATACTTTCGGACAGGACATAGCAGCTCAGTTCAACCATACCAATGGGCTCACTCTCATATCAAGAGCCCACCAGCTTGTCATGGAGGGTTATAATTGGTGTCAGGAAAAGAATGTGGTGACAGTCTTTAGCGCTCCGAACTACTGTTACCGATGTGGGAATATGGCTGCCATACTGGAAATTGGGGAAAACATGGAGCAGAATTTCCTTCAGTTTGATCCAGCACCTCGACAAATTGAGCCAGACACTTCGCGCAAGACTCCTGATTATTTTTTGTGATCTCTCAATACATCAATCAGCTGTTAGTATTTGTCGCTTGCCCTCGTGTCAACGTAGATGTGTCTTAAGAGCGGAGTTTTTGTTGTTTGAGGGTTTCATTGTCAGCATCATCATTCTTTCGGTTGGAGTTTGCCCTGCTGCTGCTGACTTCTGTTATGTGCTCAAAAGAACTCGAGACCTGATCGAAAGGAGCCATGTCCTCTTTTTTTTTTTTTCCCTTGTATATTTTTCGAGAGGAGGAAGCAGCAATAACGTGGTATATTGTGTTCCTGTAATTTCCCAATTTAAGGTAGGAATCAGATTTTGGCCGCGAGGGAGATGCATGGTATTGGTTTCTTTCCACCTTGCAACCTGCTGAGATCGTTTGACCTCATCGGACAATGAATCTATGATCTTTATAGTTTCTGTAATTGAGGTATCCATGCTTCTAATCTTGCTGTAGATCGGATTTCACTTCGGTGGTTGTGTGCTTAGTTCTATGGAAAAGGAAGGCGCGA

Unigene16896_All *RH 8*

AGCCACTGATGTTGCTGCTCGTGGATTAGACATCAAAGATATCAGGATGGTTATCAACTATGACTTTCCTACGGGAGTAGAGGATTACGTGCACAGGATTGGAAGAACTGGGAGAGCAGGTGCCACCGGGGTGGCTTTCACATTTTTGAGTGACCAAGACGCAAAGCATGCTTCAGATCTCATTAAGGTTTTGGAAGGGGCAAACCAGCGGATACCAGTTGAACTTCGCGATATGGCTTCACGTGGCGGTGGAATGGGCAGACCTAGGCGTAAGTGGGGTTCTGGATCTGGTGGACGTGATGCAAGCCATGGTGGACAGAATGGCTCGGCCTACAGTGGAAGGGATGGTGGTAGGGGCACTTGGGGGATACCAGAAAGAGTTGGTGGCCGTGGTTTTGACCATGACTCCAGAAATAGTGACAGGTATGGCCGCAGTTTCAATGATGATGTGGATGCTCATGGGAGCTACCGTAATCGGGTCCATCATGAAAGGTTGGTTCGAGCTCGAGATGGTGACCACCGAAGCAGAAGCCGGAGCCGAAGTAGAAGCCCAATCAAGGTCCCCCACAGGTTGATTCGTGCTGGAGATGGTGCTCACCGAAGTAGTAGCCGTAGCCGAAGTAGAAGCCCAAACAAGTTTTCTGGTAGGGGAGACTTTCGGAGGCGGGCTGAGAGCCGAAGCCGTAGCCGTAGCCTTGACAGGTTTGATCGGGCCCTGCCAGCCCGGGTACGTTCCCCAATCCGCAGCTCTAGTAAACATTTGTCTCCACCATACAGAAGTGACCCTGAAAGGAGATTCAAGAATTCTGTGGACCCACCACCTCGTTCCTCGTCTGGTGGTCTTAGGAAGGAAAATCACATGGGATCAGATGAAGATGAGTGGGGAAAAGCAACAAGTGGTGGACTGAATGGTGAGAGGCTTGGAAATGGACATGGGTCTTCATACTTGGGGGAGGAGGGGGAATTGGAGGAAGGAATGATTCCAGCCGACGAAGAAGGCATGATTCCAGGAGATGATTAAGATCCTCGTCCCTCTTCTCCGTAGAACTATGTGCCTGTGGATTACCATCGATTGGTGGTTGTGTTGTGCCTTCTCTTTTCTTTTCTATTTTTCGGTTGGGATGGGGCCTTGGAGTAGGGGGAGTTGTAACTGTAGCAGTGGTTTTGAAGTTGCCCTTTGGAGGGCATCGTGGTATTTATAGATTCAAAAACTCAATAAAGCAGACTTGATTATTCTGTAACCGGTTTGTATTTTCAAATTGTAATGGTGTATTGAGATTGAATGTCGTTAATTAGGTAGGAAATGTTAATCTTATTGAAGAATTGTAGCTATTTGACAGAGTAATTTTGTACTCTATTTTTTGCCTCTGTAATATCATTGGTTGGCATTGAGAATCTAAGATGTCTAAACTGTCTTCTCTTTCTTTCTAGGTGTTGTTGGTCTCTCTCTCTCTC

CL5626.Contig1_All *SAND*

TCTCTCTCTCTCTCTCTCTCAGACTCAGAGTTGGACCTGTGTTCCCTCATAGAATACGTTCGAATCTGTAATTTATTGATCTGACCGACACTCTCTCAGCAATGGGATCCGATTCGGAAT

CTCCAACGTCCTCCGACGATTCCACGTCCCAAAACCCTAATCCAAACCCTCACCCGAATTCCACTTCAATTGACCAGTCCCTCGACGCCATCGAGGACCAATTAACCTCTATCGCACTCACCCAACCTAAAGACCTTGCATCCGATGATCCTGCCTCCTCCGAAGAGGAAGAAGTATTCAAGGATGCTCCCAATGGTTCTGTATTGGAAGAAGAAAACCAGAAGGAGAGAAATGAAGGTGAGATAGTAGAAGAGGAGGTAGGAGTTGTTGCTGCGGAGGAGGAGGTTAGGGAGGAGGGGAGGTTGGGTTGGAGGAGTGTGAATTCGGAGGTGGAAGTGGACCGCTTGTCGAGCCCGAGCAGCAGTGGGTATGCGGGTGAGAGAGGGAGCAGTGGTGGTGCCAGTACTGCTAGTGGAATTGAGGAAATTGGTGAAGATCGAGGTTTTGATGGGAATGGGAGGGGTGAGTTTTTTGATGGAGCTTCAGATTCTCAGGCGCCGCCGTGGGTTCCGGGAAAGCGGCATGTCGATGAAGATGATGCTTCTGTTTCGTGGAGGAAAAGGAAAAAGCATTTCTTTATTTTGAGTCACTCAGGCAAACCTATTTATTCCAGGTATGGAGATGAACATAAGTTAGCAGGTTTTTCAGCAACTTTGCAAGCAATCATTTCGTTTGTGGAGAATGGGGGAGATCGTGTTAAATTGGTAAGGGCAGGCAAACACCAGGTGGTTTTTCTTGTGAAAGGGCCAATCTACTTGGTTTGCATAAGCTGTACGGAAGAACCTTATGAATCGTTAAGGGGGCAGTTGGAGCTTATTTATGGCCAGATGATACTTATTCTTACGAAGTCTGTGAATAGATGTTTTGAGAAGAATCCAAAGTTTGATATGACACCTTTGCTTGGAGGAACGGATGTTGTCTTCTCTTCTCTCGTCCATTCTTTCAGTTGGAACCCGGCCACTTTTCTTCATGCATATACCTGTCTTCCCCTTGCTTATGCAACAAGACAAGCCGCAGGTGCTATATTGCATGACGTAGCTGATTCTGGTGTCCTCTTTGCAATATTAATGTGTAAACACAAGGTAGTCAGTCTTGTTGGTGCACAAAAAGCATCTCTTCATCCTGATGATATGCTCCTACTTTCCAATTTTGTTACATCATCTGAATCATTTAGGACATCTGAATCTTTCTCACCAATTTGCCTGCCAAGATACAATCCCATGGCATTTTTGTATGCTTATGTCAATTATCTTGATGTGGACACCTACTTAATGTTGCTTACTACCAGTTCAGATGCCTTCTATCATCTAAAAGATTGCAGGATTCGTATCGAAATGGTCCTTCTGAAGTCAAACGTTCTTAGTGAAGTTCAGAGATCCATGTTGGATGGTGGCATGCGCGTTGAGGATTTGCCTGTTGATCCATCTCCTCGTTCTGAATTTTCATCACCTCATTTAGGTCAGTCCCGAATTGCGACAGACTCGCCGGATAGATTCCGAGAAGCATTTGCTGGTATTGGTGGTCCTGCTGGACTTTGGCATTTCATATACCGTAGTATTTTCCTGGACCAATATGTTTCTTCTGAGTTCGCATCACCGATCAACAGTTCGCAACAGCAGAAAAGATTGTATAGAGCTTACCAAAAGCTTTATGCCTCTATGCATGATAAAGTAATTGGACCCCACAAAACTCAGTTCAGAAGAGATGAGAACTATGTTTTGCTCTGCTGGATCACCCCGGACTTCGAACTCTATGCGGCATTTGATCCACTTGCAGACAAGGCTTTGGCAATAAGTACTTGTAACCGGGTGTGTCAATGGGTGAAAGATGTGGAAAATGAAATTTTCCTGTTGGGAGCTAGCCCCTTTTCATGGTGATTCTCCTCAAAATACCTTGTATCACAGGTTGTACCCTAGATTTTTTTTTTTTTTGGTGTAGTATTTTTTTTCCATCTATAAAATCCTTACTATATTTTTTTGACCTGAACTGTATTCTCCATGTTAGTCGTTTGACCTTCATAGATGGCAGTCTGACGCCC

Unigene2152_All T*UBB*

CTCTCTCTCTCTCTCTCTCTACGACCCAATTCAAACGATGAGAGAGATCCTCCACATCCAAGGCGGCCAATGCGGCAACCAGATCGGCGCCAAGTTCTGGGAGGTGGTGTGCGCGGAGCACGGCATCGACTCCACCGGCCGCTACAACGGCGACTCCGATCTCCAGCTTGAGCGTGTCAATGTCTACTACAACGAGGCCAGTTGTGGCCGGTTCGTCCCGCGTGCCGTCCTCATGGACCTCGAGCCCGGTACCATGGACTCGGTCCGATCCGGCCCCTTTGGCCAGATCTTCCGGCCCGATAACTTCGTGTTTGGGCAGTCTGGGGCGGGGAATAATTGGGCTAAAGGGCATTATACGGAGGGTGCTGAGCTGATTGATTCGGTTCTTGATGTTGTGAGGAAGGAGGCTGAGAATTGTGATTGTCTGCAGGGATTTCAAGTTTGTCATTCATTGGGTGGAGGCACGGGATCTGGTATGGGCACCCTTCTCATTTCCAAGATCAGGGAAGAGTATCCAGATCGAATGATGTTGACATTTTCAGTCTTTCCTTCTCCGAAAGTATCCGACACTGTTGTTGAGCCCTACAATGCCACCCTTTCTGTTCATCAACTTGTTGAGAATGCTGATGAATGTATGGTTTTGGACAATGAAGCACTCTATGACATATGCTTTCGTACCCTGAAGCTTTCAACACCCACTTTTGGTGATCTCAACCACCTGATCTCTGCCACAATGAGTGGTGTTACATGCTGTCTTCGTTTCCCTGGACAACTCAACTCCGACCTTCGGAAACTCGCGGTTAACCTAATCCCATTCCCTCGTCTCCACTTCTTCATG

Unigene320_All *18s rRNA*

AAAAGTCTAAAACTCCAGTACTAGTACGTTCAGTTCACCTTATATTCATCAGTTAGATCAGTTAGGGTTTTCTTTTACCCACCGCCTCCTCCACCCAGCCTCGCCTCCTCCACCGCCTCTCTCCGACTCTTCCACGACGCCTCTTCTCCGTTACCGATTCACCGGTGTCGTGCTGATGTCCTAAAACCTCTACGCCTCTTCTTCATAATCGATAAACAGGATCAACCATGGCAGGAGGGAAGATCAAGAGGGAGAAGCCCCATCATGGTGGCACTCCTGCCTCAGCAAACCCGCACTACCAAGGGGGCATACCATTCCACAAGTCAAAGGGTCAGCACATCCTTAAAAACCCTCTGCTGATTGACACAATCATTGAGAAATCAGGCATCAAGAGCACTGATATTATCCTCGAGATTGGCCCCGGTACTGGAAACCTGACGAAGAAGCTTCTGGAAGCCGGAAAATCTGTCATTGCAGTCGAGCTTGACCCCCGTATGGTCCTCGAGCTCCAGCGCCGCTTCCAGGGCACTGCCTTCTCCAACCGCCTCAAGGTTATCCAAGGAGATGTGCTTAAGTGTGATCTCCCATACTTCGATATCTGTGTGGCAAACATACCTTACCAAATTTCCTCTCCTCTCACTTTCAAATTGCTAGCCCATCGTCCATTATTCAGGTGTGCAGTTATAATGTTCCAGAGGGAATTCGCCATGAGACTTGTTGCTAAGCCCGGTGACACTCTTTACTGCCGCCTTTCTGTGAACACCCAACTCTTATCTCGCGTTTCCCATTTGCTAAAAGTTGGAAAGAACAATTTCCGGCCTCCACCAAAGGTTGATTCTTCTGTAGTTAGAATCGAGCCAAGAAAACCACAGCCTTCAGTCAGTTTCAAAGAATGGGATGGTTTAGTCCGGATTTGCTTCAACCGAAAGAACAAAACCCTAGGTTCAATATTTAGACAGAAATCGGTGTTGTCAATTCTAGAGAAGAACTATAAAACATTGCAAGCGTTACAGCTCTCGCAGAATGGATCACTAGAGGATACTGAGATTGCAATGGATGTATCCAGTTTGGGGAACGCGGGTGAGGAGTTAAGTATGGAGATCGATGATGAAAGAGATGAGGAGGAGATGGAGGTAGAGGAGGGGGACATGAGAACGTCCGATTTCAAGGATAAAGTTTTGGGTGTGTTGAAGCAAGGGGGTTTTGAAGAGAAGAGGTCATCCAAGCTCAGTCAAGAAGATTTCATGTACCTGCTCTCTGTGTTTAACAAGGCTGGAATACATTTCTCTTGATTGCCGGGCTATGAATTTACATACCCTTGTTTTTTTTTTTGTGTGGCTAATTTAGTTGTAATTTTTTTTTTTTAAATGTTGAGTTTCTATTCCAATTTTGTGAACGGTGAGGGATGGTAAGATATAGGATGAGCTGATGAGGCCTTTTGAATTCTGTTGGAGGATACTTGTTTTTGAGCGTGCTCGCACTTGGGAGTT

>CL1200.Contig2_All *cytochrome P450*

CTTACTTTTCTTCTTCCTCTTCTTCTTCTTCTTGCAGTCGTTTGGACAATGGTGCGGAACAGTGCCACCAAACGGAGACCACCAGGGCCAACGGGATGGCCTGTGATTGGCAATATGTTTGATCTTGGAGCTATGCCACACATAGACATGTACAAGCTCAGATTCAAATATGGACCAGTAATTTGGTTTAAGTTTGGATCGGTAAACACCATGGTTATACAATCACCCAAGGCAGCCGCAGAGTTTTTCAAGAACCACGATTCAAGTTTCTGCGACCGAAATGTCACCGACGTAATGACATCGTGCAGCTACAACCAAGGATCACTTGCTCTGAGCAAATATGGCACGTATTGGCGACTACTAAGGAGAATATGCTCCTCGGAGCTACTGGTCACCAAACGAGTCAACAGTACAGCTGCTCTCAGGGAGAAGTGTGTCCAGAACATGATCCGGTGGATCGAGGAGGATTCACAGGCGTCACATGCAAGTGGAGGATCGGGAGAGGTACAGATAAGCCATTTCCTCTTCCTCATGTCTTTCAATGTGGTAGGCAATCTCATGCTATCTAGAGATCTGTTGGAACCGAACTCAGACAAAGGGCGTGAATTCCTCGAGGCAATGGAAATGATAATGGTGGTTTCGGGGAAGCCTAACGTAGCTGATTTCCTTCCATTTCTGAAATGGATGGACCCCATGGGGCTCAAAAGAGATATGGATCTGCACTTGGGACGAGCCCTGAAGATTGTGGAAGAGTTTGTAACGGAGAGGGTTCGGGAGAAGAAGAATTCAGGTACTGAGAAGGTGAACAAAGACTTCTTGGAT

>CL3597.Contig4_All *cytochrome P450*

ATGGATTTACTTTCTCATCTACCACTAGCAATTGCCTTGCTTTTAGCCTCACTCTTCCTGTACAATCGGTGGAGATCAAGAACTCATAGCCACAACATCAAGTTCAAGTCACCCCCTGAACCACCGGGCGCATGGCCCATCACAGGCCACCTTCATCTACTCCGCGGCCAAGCTCCCCTGGCTCGAACCTTGGGAGCCATGGCCGACAAATACGGCCCGGTCTTCTCGTTCCGGCTCGGCGTCCACCCTGTAGTCGTGGTGAGCAATTGGGAGTCTTTCAAAGAGTGTTTCACCACCCATGACAAAGCCTTCGCCTCCCGCCCTCAATCCGGTGCATGTAAGCTGATA
