# Supplementary material for: Selection of reference genes for normalization of cranberry (Vaccinium macrocarpon Ait.) gene expression under different experimental conditions
Source: PLoS One. 2019 Nov 12;14(11):e0224798. doi: 10.1371/journal.pone.0224798 (PMC6850891; doi:10.1371/journal.pone.0224798)
Supplement: S1 Table — (DOC) [file pone.0224798.s004.doc]

**Table S1. Differentially expressed gene sequences selected from 10 candidate gene families.**

| **Gene Name** | **Gene ID** | **R_FPKM** | **W_FPKM** | **log2(R_FPKM/W_FPKM)** | **gene Length** |
| --- | --- | --- | --- | --- | --- |
| ***Actin*** | CL3266.Contig2_All | 1.3852 | 2.5714 | -0.892459757 | 569 |
|  | CL383.Contig3_All | 5.9347 | 4.7197 | 0.330479943 | 1000 |
|  | CL383.Contig4_All | 1.1689 | 1.5865 | -0.440696009 | 357 |
|  | CL7164.Contig1_All | 2.8286 | 3.8809 | -0.456303084 | 377 |
|  | CL7164.Contig2_All | 191.4676 | 163.5103 | 0.227718763 | 1813 |
|  | CL7164.Contig3_All | 195.395 | 209.5471 | -0.100881005 | 1787 |
|  | CL7164.Contig4_All | 13.6589 | 20.7018 | -0.599914911 | 611 |
|  | CL7164.Contig5_All | 9.3286 | 8.2914 | 0.170044862 | 666 |
|  | CL7164.Contig6_All | 4.1684 | 1.8611 | 1.16333815 | 634 |
|  | CL7164.Contig7_All | 33.5053 | 31.4889 | 0.089545964 | 649 |
|  | CL7164.Contig8_All | 4.2963 | 0.5467 | 2.974273456 | 259 |
|  | CL7164.Contig9_All | 7.1267 | 4.9198 | 0.534634527 | 566 |
|  | Unigene10093_All | 7.549 | 9.2922 | -0.29973466 | 1732 |
|  | Unigene1137_All | 0.9935 | 2.8655 | -1.528195024 | 280 |
|  | Unigene1225_All | 1.5119 | 1.71 | -0.177633605 | 276 |
|  | Unigene25299_All | 1.815 | 2.5194 | -0.473110646 | 281 |
|  | Unigene25681_All | 1.1991 | 1.6275 | -0.440705565 | 232 |
|  | Unigene27506_All | 0.5778 | 1.7644 | -1.610535557 | 321 |
|  | Unigene29623_All | 0.3864 | 0.7866 | -1.025535092 | 240 |
|  | Unigene29807_All | 3.3231 | 1.1276 | 1.559274325 | 293 |
|  | Unigene36_All | 5.4323 | 6.4808 | -0.254608758 | 1340 |
|  | Unigene38604_All | 1.4797 | 0.6695 | 1.144148747 | 282 |
|  | Unigene6321_All | 3.0199 | 2.5316 | 0.254451305 | 261 |
| ***CYP 2*** | CL1035.Contig1_All | 3.3606 | 4.6319 | -0.462885273 | 1559 |
|  | CL1404.Contig1_All | 7.8825 | 6.3793 | 0.305255139 | 947 |
|  | CL1404.Contig2_All | 66.5571 | 63.214 | 0.074348467 | 2652 |
|  | CL1573.Contig2_All | 0.8701 | 1.1388 | -0.388261275 | 373 |
|  | CL1573.Contig4_All | 0.3384 | 0.1723 | 0.973806867 | 548 |
|  | CL1818.Contig1_All | 11.6155 | 11.9234 | -0.037744426 | 475 |
|  | CL1818.Contig2_All | 22.5834 | 25.3691 | -0.167809634 | 813 |
|  | CL404.Contig1_All | 7.0985 | 6.5224 | 0.122111278 | 2482 |
|  | CL4850.Contig1_All | 747.7516 | 738.7621 | 0.017449237 | 838 |
|  | CL4850.Contig2_All | 619.956 | 596.4345 | 0.055802116 | 824 |
|  | CL4850.Contig3_All | 0.7912 | 1.2887 | -0.703802123 | 293 |
|  | CL5471.Contig1_All | 2.5465 | 1.7878 | 0.510330365 | 528 |
|  | CL5947.Contig2_All | 15.0954 | 13.2792 | 0.184940751 | 2623 |
|  | CL5947.Contig3_All | 1.8394 | 0.5165 | 1.832394991 | 731 |
|  | CL945.Contig1_All | 0.7475 | 0.878 | -0.23214736 | 2419 |
|  | CL945.Contig2_All | 0.15 | 0.3709 | -1.306067767 | 2163 |
|  | Unigene12814_All | 25.7101 | 26.2117 | -0.027875703 | 1183 |
|  | Unigene13052_All | 112.8448 | 119.7405 | -0.08557126 | 756 |
|  | Unigene13318_All | 10.613 | 11.6997 | -0.140639013 | 948 |
|  | Unigene13402_All | 10.0289 | 9.1357 | 0.134576194 | 1803 |
|  | Unigene13612_All | 51.9858 | 57.085 | -0.134994101 | 1031 |
|  | Unigene14256_All | 65.3459 | 67.5047 | -0.046891233 | 1341 |
|  | Unigene14838_All | 208.1694 | 258.8646 | -0.314439674 | 394 |
|  | Unigene14872_All | 1.6073 | 1.5103 | 0.089804082 | 375 |
|  | Unigene15042_All | 25.784 | 23.2589 | 0.148693226 | 1390 |
|  | Unigene18257_All | 18.6201 | 17.7777 | 0.066792134 | 1123 |
|  | Unigene18865_All | 1.9068 | 3.6777 | -0.947650269 | 462 |
|  | Unigene26664_All | 0.7994 | 2.0344 | -1.347613889 | 232 |
|  | Unigene30217_All | 1.4956 | 0.9135 | 0.711247742 | 310 |
|  | Unigene32396_All | 2.917 | 1.7191 | 0.762831917 | 302 |
|  | Unigene34889_All | 1.4869 | 0.4128 | 1.848792748 | 343 |
|  | Unigene3898_All | 21.7674 | 23.7093 | -0.123283973 | 1065 |
|  | Unigene3974_All | 6.3813 | 5.5873 | 0.191699075 | 1039 |
|  | Unigene4044_All | 29.28 | 38.7201 | -0.403167125 | 863 |
|  | Unigene4766_All | 31.0394 | 29.7148 | 0.062918999 | 953 |
|  | Unigene4864_All | 27.4713 | 20.0213 | 0.456389531 | 1735 |
|  | Unigene6308_All | 3.1918 | 1.026 | 1.637339523 | 276 |
|  | Unigene9153_All | 2.7739 | 3.8322 | -0.466257077 | 234 |
|  | Unigene9524_All | 18.8468 | 16.4132 | 0.199463048 | 647 |
| ***EF-1α*** | CL537.Contig1_All | 9.2531 | 15.0103 | -0.697944124 | 937 |
|  | CL537.Contig2_All | 7.3821 | 8.4374 | -0.192767217 | 358 |
|  | CL5384.Contig1_All | 0.4228 | 0.5738 | -0.440572591 | 329 |
|  | CL5384.Contig2_All | 4.761 | 4.3936 | 0.115861098 | 1042 |
|  | CL6870.Contig1_All | 1.8468 | 4.9473 | -1.421613748 | 477 |
|  | CL6870.Contig2_All | 2.2369 | 0.621 | 1.848835589 | 228 |
|  | CL897.Contig1_All | 66.8176 | 61.4843 | 0.120010098 | 1546 |
|  | CL897.Contig2_All | 1.6559 | 1.2642 | 0.389390831 | 224 |
|  | Unigene10645_All | 2.0606 | 1.1537 | 0.836796354 | 450 |
|  | Unigene13249_All | 81.1282 | 92.3342 | -0.186661632 | 2406 |
|  | Unigene14606_All | 228.7742 | 194.3405 | 0.235337776 | 289 |
|  | Unigene1527_All | 1.2201 | 2.6615 | -1.12524017 | 266 |
|  | Unigene16491_All | 399.1223 | 239.3945 | 0.737440881 | 263 |
|  | Unigene17242_All | 82.943 | 68.0391 | 0.285756172 | 976 |
|  | Unigene17504_All | 8.7193 | 8.101 | 0.10611231 | 670 |
|  | Unigene18891_All | 0.8613 | 2.3379 | -1.440625486 | 323 |
|  | Unigene18911_All | 54.8789 | 43.9094 | 0.321721743 | 1895 |
|  | Unigene21117_All | 269.8034 | 338.9127 | -0.329005171 | 1814 |
|  | Unigene21932_All | 2.1203 | 2.1584 | -0.025693848 | 328 |
|  | Unigene22183_All | 6.6381 | 6.0296 | 0.138708065 | 454 |
|  | Unigene22953_All | 3.2146 | 5.7895 | -0.848799527 | 375 |
|  | Unigene23825_All | 1.352 | 5.9097 | -2.127989744 | 583 |
|  | Unigene24131_All | 26.773 | 29.1775 | -0.124077468 | 736 |
|  | Unigene24809_All | 2.2918 | 2.5124 | -0.132585025 | 263 |
|  | Unigene25111_All | 3.3034 | 5.5351 | -0.744657709 | 1586 |
|  | Unigene26880_All | 0.552 | 1.8729 | -1.7625337 | 252 |
|  | Unigene27629_All | 1.9922 | 5.1622 | -1.373623548 | 256 |
|  | Unigene27630_All | 1.4173 | 2.4732 | -0.803233745 | 229 |
|  | Unigene27660_All | 0.4049 | 1.6488 | -2.025778862 | 229 |
|  | Unigene27711_All | 0.3848 | 4.5043 | -3.549122214 | 241 |
|  | Unigene27864_All | 0.6265 | 1.7539 | -1.485180079 | 296 |
|  | Unigene30189_All | 1.4235 | 0.621 | 1.19677732 | 228 |
|  | Unigene32850_All | 1.9039 | 1.026 | 0.891926974 | 414 |
|  | Unigene35364_All | 2.7346 | 2.0245 | 0.433764177 | 373 |
|  | Unigene373_All | 4.7255 | 4.8289 | -0.0312276 | 2551 |
|  | Unigene457_All | 7.4412 | 7.4777 | -0.007059297 | 486 |
|  | Unigene634_All | 2.0885 | 2.126 | -0.025674453 | 444 |
|  | Unigene8673_All | 2.8431 | 2.2263 | 0.352816831 | 212 |
|  | Unigene9998_All | 17.1572 | 18.8379 | -0.134824017 | 1616 |
| ***F-box*** | CL1020.Contig2_All | 5.0124 | 6.5656 | -0.389425309 | 1258 |
|  | CL1166.Contig1_All | 0.2722 | 0.4618 | -0.762601106 | 511 |
|  | CL1166.Contig2_All | 4.4512 | 5.4739 | -0.298374754 | 1552 |
|  | CL1365.Contig2_All | 8.0394 | 12.2196 | -0.604037322 | 421 |
|  | CL1562.Contig4_All | 10.12 | 9.9403 | 0.025847992 | 1828 |
|  | CL1905.Contig2_All | 4.071 | 6.7534 | -0.730230789 | 615 |
|  | CL224.Contig1_All | 1.1039 | 1.1237 | -0.025647436 | 420 |
|  | CL2412.Contig2_All | 2.3291 | 2.6303 | -0.175454773 | 1274 |
|  | CL4451.Contig1_All | 29.2734 | 22.3584 | 0.388773369 | 2588 |
|  | CL4888.Contig2_All | 0.5937 | 0.0604 | 3.297113658 | 781 |
|  | CL4888.Contig3_All | 0.4404 | 0.1921 | 1.19695695 | 737 |
|  | CL4966.Contig1_All | 3.6937 | 4.4335 | -0.263379379 | 841 |
|  | CL5056.Contig1_All | 2.1222 | 1.6437 | 0.368613615 | 1005 |
|  | CL5145.Contig2_All | 8.6365 | 4.1644 | 1.05233812 | 306 |
|  | CL5190.Contig1_All | 2.6444 | 2.4227 | 0.126324651 | 1578 |
|  | CL5584.Contig1_All | 2.385 | 2.7722 | -0.217042078 | 2741 |
|  | CL5584.Contig2_All | 0.9257 | 1.2369 | -0.41811224 | 2404 |
|  | CL5737.Contig3_All | 0.3589 | 0.7306 | -1.025499831 | 646 |
|  | CL581.Contig2_All | 1.7752 | 2.2323 | -0.330549352 | 444 |
|  | CL6227.Contig1_All | 3.9459 | 3.4142 | 0.208806822 | 235 |
|  | CL6548.Contig2_All | 7.3933 | 4.7197 | 0.647523295 | 740 |
|  | CL6656.Contig1_All | 2.3934 | 2.833 | -0.243269072 | 833 |
|  | CL6656.Contig2_All | 0.2557 | 0.7808 | -1.610500892 | 544 |
|  | CL94.Contig1_All | 1.0079 | 2.052 | -1.025678224 | 322 |
|  | Unigene10089_All | 7.7936 | 6.9069 | 0.174251552 | 1517 |
|  | Unigene10599_All | 2.1436 | 2.3437 | -0.128752192 | 584 |
|  | Unigene1299_All | 1.65 | 1.1757 | 0.488946046 | 281 |
|  | Unigene13223_All | 6.2549 | 5.7305 | 0.126325796 | 593 |
|  | Unigene13653_All | 2.4864 | 2.68 | -0.108174592 | 317 |
|  | Unigene1404_All | 37.2196 | 43.0544 | -0.210098132 | 1014 |
|  | Unigene15143_All | 1.7193 | 1.2251 | 0.488921787 | 809 |
|  | Unigene1556_All | 2.529 | 3.218 | -0.347597289 | 220 |
|  | Unigene15622_All | 2.4185 | 2.4619 | -0.025659624 | 786 |
|  | Unigene16093_All | 1.686 | 3.0893 | -0.873675441 | 275 |
|  | Unigene16194_All | 2.7159 | 2.1722 | 0.322273418 | 478 |
|  | Unigene17278_All | 5.1279 | 4.6762 | 0.133031493 | 434 |
|  | Unigene1740_All | 7.782 | 5.5035 | 0.499791575 | 566 |
|  | Unigene1783_All | 7.6378 | 6.4608 | 0.241444327 | 862 |
|  | Unigene17930_All | 16.7659 | 19.069 | -0.185699259 | 943 |
|  | Unigene18946_All | 31.2419 | 48.1233 | -0.623253382 | 1070 |
|  | Unigene20218_All | 26.5758 | 35.7986 | -0.429790047 | 1619 |
|  | Unigene2121_All | 70.3667 | 60.5517 | 0.216725392 | 311 |
|  | Unigene2318_All | 5.5933 | 4.6194 | 0.275994236 | 1318 |
|  | Unigene24412_All | 265.0723 | 394.5666 | -0.573882921 | 350 |
|  | Unigene25106_All | 0.7151 | 1.2133 | -0.762719406 | 389 |
|  | Unigene25194_All | 0.6462 | 0.1644 | 1.974770452 | 574 |
|  | Unigene26931_All | 1.7832 | 2.8238 | -0.663169389 | 234 |
|  | Unigene27189_All | 0.6983 | 1.8481 | -1.404123945 | 332 |
|  | Unigene27394_All | 2.8532 | 4.1751 | -0.549229874 | 260 |
|  | Unigene2966_All | 22.4797 | 21.9895 | 0.031807982 | 1584 |
|  | Unigene31645_All | 1.6832 | 1.4019 | 0.263823167 | 303 |
|  | Unigene3884_All | 1.963 | 1.237 | 0.666214672 | 496 |
|  | Unigene4806_All | 1.6425 | 1.596 | 0.041432719 | 621 |
|  | Unigene5236_All | 4.3518 | 5.2379 | -0.267376262 | 1694 |
|  | Unigene5288_All | 21.3228 | 27.5883 | -0.371659662 | 1733 |
|  | Unigene5822_All | 17.7489 | 17.5606 | 0.015387477 | 1024 |
|  | Unigene7005_All | 2.8673 | 2.5333 | 0.178674924 | 857 |
|  | Unigene7690_All | 8.0281 | 7.8049 | 0.04067844 | 1542 |
|  | Unigene9199_All | 20.277 | 12.9158 | 0.650707214 | 1173 |
| ***GAPDH*** | CL3606.Contig3_All | 1.5951 | 2.2732 | -0.511077748 | 872 |
|  | CL441.Contig1_All | 2.0648 | 3.0573 | -0.566256078 | 247 |
|  | CL441.Contig2_All | 6.6668 | 7.095 | -0.089808237 | 612 |
|  | CL441.Contig4_All | 4.2398 | 3.2022 | 0.404932794 | 339 |
|  | CL441.Contig5_All | 450.9319 | 294.0295 | 0.616948665 | 248 |
|  | CL441.Contig6_All | 3.9163 | 3.7723 | 0.05404687 | 1101 |
|  | CL441.Contig7_All | 473.117 | 347.8027 | 0.443927868 | 279 |
|  | CL7019.Contig1_All | 3.8637 | 5.7839 | -0.58205953 | 204 |
|  | CL7019.Contig2_All | 0.8316 | 1.4815 | -0.833096963 | 223 |
|  | CL792.Contig2_All | 1.5529 | 0.4516 | 1.781847538 | 209 |
|  | CL792.Contig4_All | 1.3374 | 0.9076 | 0.559302508 | 208 |
|  | Unigene10846_All | 7.8463 | 8.158 | -0.05620301 | 1105 |
|  | Unigene13040_All | 495.8649 | 381.4803 | 0.37833855 | 967 |
|  | Unigene14862_All | 72.7714 | 81.4186 | -0.161986848 | 1826 |
|  | Unigene16420_All | 1.1111 | 4.4215 | -1.992547223 | 459 |
|  | Unigene18643_All | 56.547 | 52.0802 | 0.118715498 | 2049 |
|  | Unigene20107_All | 3.8218 | 3.9587 | -0.050774458 | 1383 |
|  | Unigene2257_All | 2.8581 | 2.4245 | 0.237369141 | 292 |
|  | Unigene2265_All | 24.1948 | 43.6165 | -0.850176992 | 435 |
|  | Unigene30794_All | 1.1591 | 0.472 | 1.296146274 | 200 |
|  | Unigene3692_All | 83.5199 | 74.16 | 0.171478741 | 2034 |
|  | Unigene4318_All | 320.7488 | 275.5203 | 0.219285249 | 1768 |
|  | Unigene4918_All | 1.076 | 1.8919 | -0.814157756 | 474 |
|  | Unigene6792_All | 198.3251 | 175.0401 | 0.180181809 | 436 |
| ***PP2A*** | Unigene21533_All | 35.7313 | 36.0478 | -0.01272281 | 1452 |
|  | Unigene22115_All | 19.1434 | 17.3721 | 0.140074923 | 1986 |
|  | Unigene38303_All | 1.5053 | 0.9194 | 0.711286467 | 308 |
|  | Unigene104_All | 1.8935 | 1.6522 | 0.196667085 | 857 |
|  | Unigene105_All | 1.9601 | 3.1465 | -0.682820684 | 615 |
|  | Unigene11111_All | 2.954 | 1.4381 | 1.038505827 | 361 |
|  | Unigene16942_All | 19.4513 | 22.2756 | -0.195597713 | 553 |
|  | Unigene5237_All | 31.9677 | 32.9744 | -0.044731459 | 1636 |
|  | Unigene967_All | 15.4125 | 15.7323 | -0.029628708 | 1095 |
| ***TUBB*** | Unigene1892_All | 22.2711 | 16.0758 | 0.470282283 | 229 |
|  | Unigene7287_All | 19.364 | 14.992 | 0.369184141 | 340 |
|  | CL5536.Contig2_All | 3.0415 | 1.2838 | 1.244362538 | 625 |
|  | CL568.Contig1_All | 41.6846 | 51.4505 | -0.303670606 | 1387 |
|  | CL568.Contig2_All | 24.319 | 25.4218 | -0.063982278 | 1346 |
|  | CL568.Contig3_All | 173.3041 | 211.1899 | -0.285235055 | 1854 |
|  | CL568.Contig4_All | 3.032 | 7.7161 | -1.347602087 | 367 |
|  | Unigene10938_All | 16.4177 | 17.3497 | -0.079658687 | 963 |
|  | Unigene11733_All | 15.9221 | 14.2712 | 0.157923979 | 463 |
|  | Unigene13672_All | 2.0158 | 1.1726 | 0.781641545 | 322 |
|  | Unigene13713_All | 2.176 | 1.0223 | 1.089859931 | 277 |
|  | Unigene13852_All | 2.9626 | 5.4284 | -0.87366318 | 313 |
|  | Unigene14656_All | 3.5078 | 5.2786 | -0.589588852 | 304 |
|  | Unigene14817_All | 5.2661 | 5.9433 | -0.174529288 | 405 |
|  | Unigene1714_All | 38.7206 | 38.0127 | 0.026619806 | 370 |
|  | Unigene17263_All | 20.3084 | 17.3576 | 0.226509098 | 242 |
|  | Unigene1997_All | 41.2822 | 44.0202 | -0.092645843 | 520 |
|  | Unigene2152_All | 15.0491 | 15.3193 | -0.025673166 | 838 |
|  | Unigene21777_All | 28.6806 | 29.309 | -0.03126854 | 1248 |
|  | Unigene24001_All | 8.8758 | 3.9232 | 1.177846275 | 397 |
|  | Unigene24102_All | 43.9599 | 57.1134 | -0.377641167 | 752 |
|  | Unigene24254_All | 14.5038 | 13.1909 | 0.136887935 | 780 |
|  | Unigene34463_All | 7.9872 | 3.1729 | 1.331887787 | 238 |
|  | Unigene5013_All | 3.0089 | 8.075 | -1.4242261 | 339 |
|  | Unigene5944_All | 5.1319 | 5.7645 | -0.167702419 | 262 |
|  | Unigene9435_All | 2.4907 | 3.5105 | -0.495125266 | 484 |
| ***RH 8*** | CL4442.Contig1_All | 12.3982 | 8.5304 | 0.539445384 | 1350 |
|  | Unigene16896_All | 24.6959 | 23.8742 | 0.048819156 | 1455 |
| ***SAND*** | CL5626.Contig1_All | 10.3343 | 10.4761 | -0.019661066 | 2158 |
|  | CL5626.Contig2_All | 0.1307 | 0.3105 | -1.248334127 | 1064 |
| ***18s rRNA*** | Unigene8648_All | 3.3874 | 3.2327 | 0.067438728 | 219 |
|  | Unigene21930_All | 6.2302 | 9.1444 | -0.553610035 | 320 |
|  | Unigene24755_All | 4.5981 | 9.3614 | -1.025686459 | 242 |
|  | Unigene31135_All | 4.4612 | 1.9463 | 1.196697725 | 291 |
|  | Unigene443_All | 1.9603 | 6.0731 | -1.631358667 | 544 |
|  | Unigene8291_All | 5.1516 | 4.0339 | 0.352845258 | 585 |
|  | Unigene9091_All | 18.5458 | 10.1386 | 0.871234052 | 270 |
|  | CL4474.Contig2_All | 13.0087 | 23.4287 | -0.848800109 | 278 |
|  | CL4518.Contig1_All | 2.4304 | 2.6643 | -0.132562764 | 248 |
|  | CL6082.Contig3_All | 2432.9504 | 868.936 | 1.485385082 | 4795 |
|  | CL7039.Contig1_All | 4.681 | 10.4378 | -1.156928994 | 208 |
|  | Unigene17211_All | 27.944 | 4.337 | 2.6877692 | 370 |
|  | Unigene18079_All | 3.971 | 1.2018 | 1.724305534 | 432 |
|  | Unigene22200_All | 11.7119 | 12.7825 | -0.126194885 | 384 |
|  | Unigene31183_All | 7.29 | 0.9895 | 2.881147202 | 477 |
|  | Unigene33913_All | 5.3951 | 0.6865 | 2.974318075 | 275 |
|  | Unigene36115_All | 2.2369 | 0.621 | 1.848835589 | 228 |
|  | Unigene5645_All | 8.7052 | 2.697 | 1.690521922 | 490 |
|  | Unigene6657_All | 4.1728 | 0.708 | 2.559194509 | 200 |
|  | Unigene7115_All | 34.1468 | 10.3642 | 1.720141625 | 296 |
|  | Unigene320_All | 13.9093 | 13.6523 | 0.026905794 | 1490 |
