# Supplementary material for: Selection of reference genes for normalization of cranberry (Vaccinium macrocarpon Ait.) gene expression under different experimental conditions
Source: PLoS One. 2019 Nov 12;14(11):e0224798. doi: 10.1371/journal.pone.0224798 (PMC6850891; doi:10.1371/journal.pone.0224798)
Supplement: S2 Table — (DOC) [file pone.0224798.s005.doc]

**Table S2. Primer pair amplification specificities for qRT-PCR**

| **Gene** | **Amplification plots** | **Dissociation curve** | **Standard curves** |
| --- | --- | --- | --- |
| **The amplification specificity of ten candidate reference genes.** | | | |
| ***ACTIN*** | 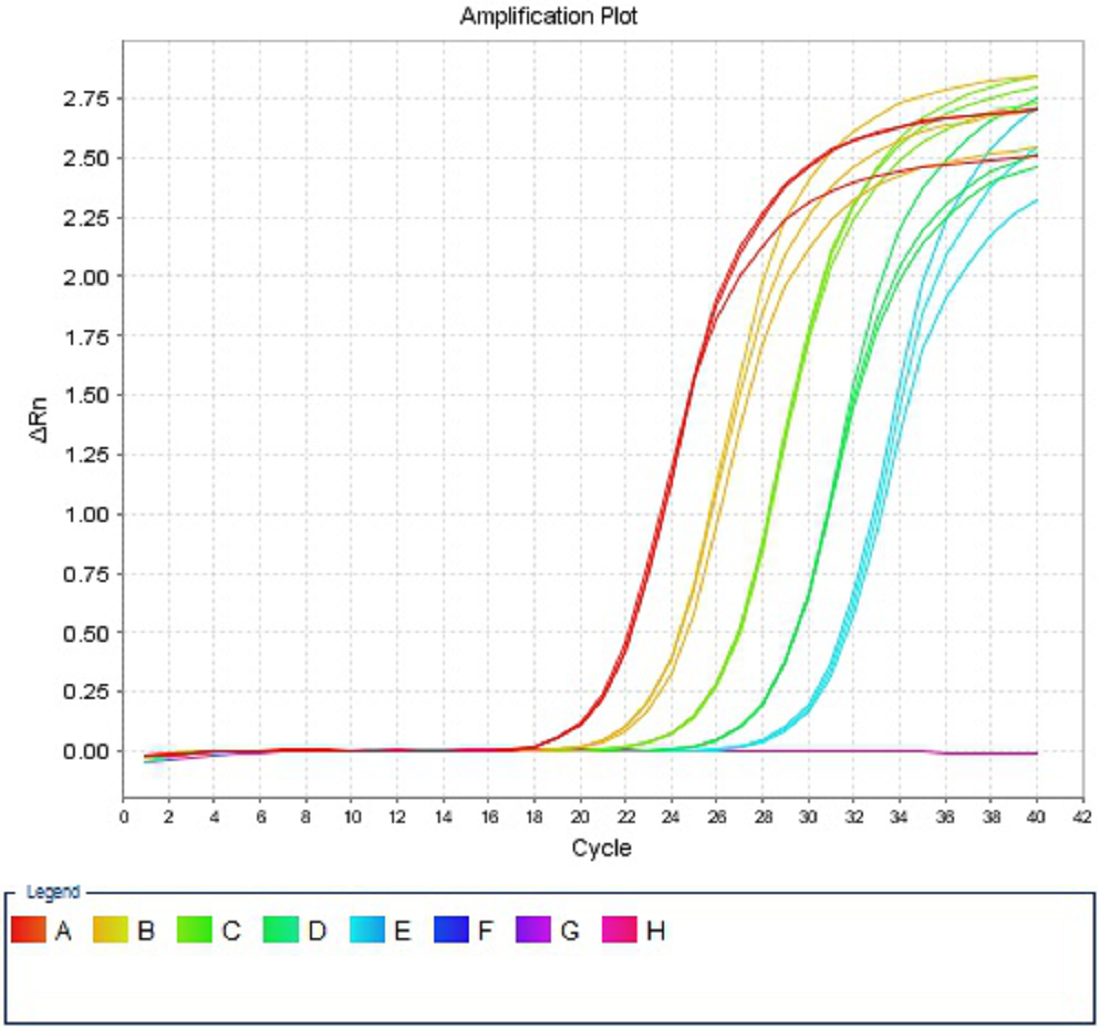 | 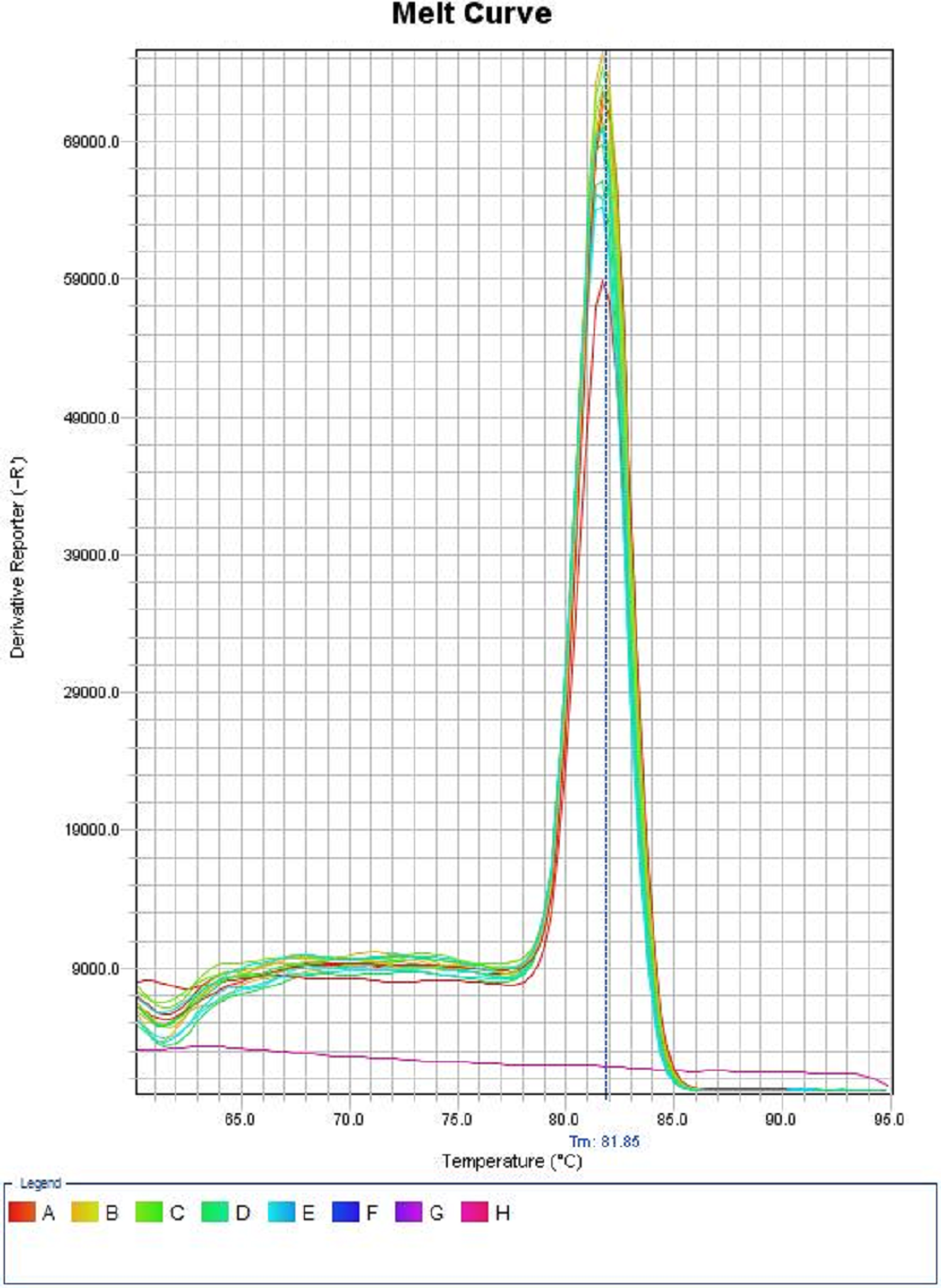 | 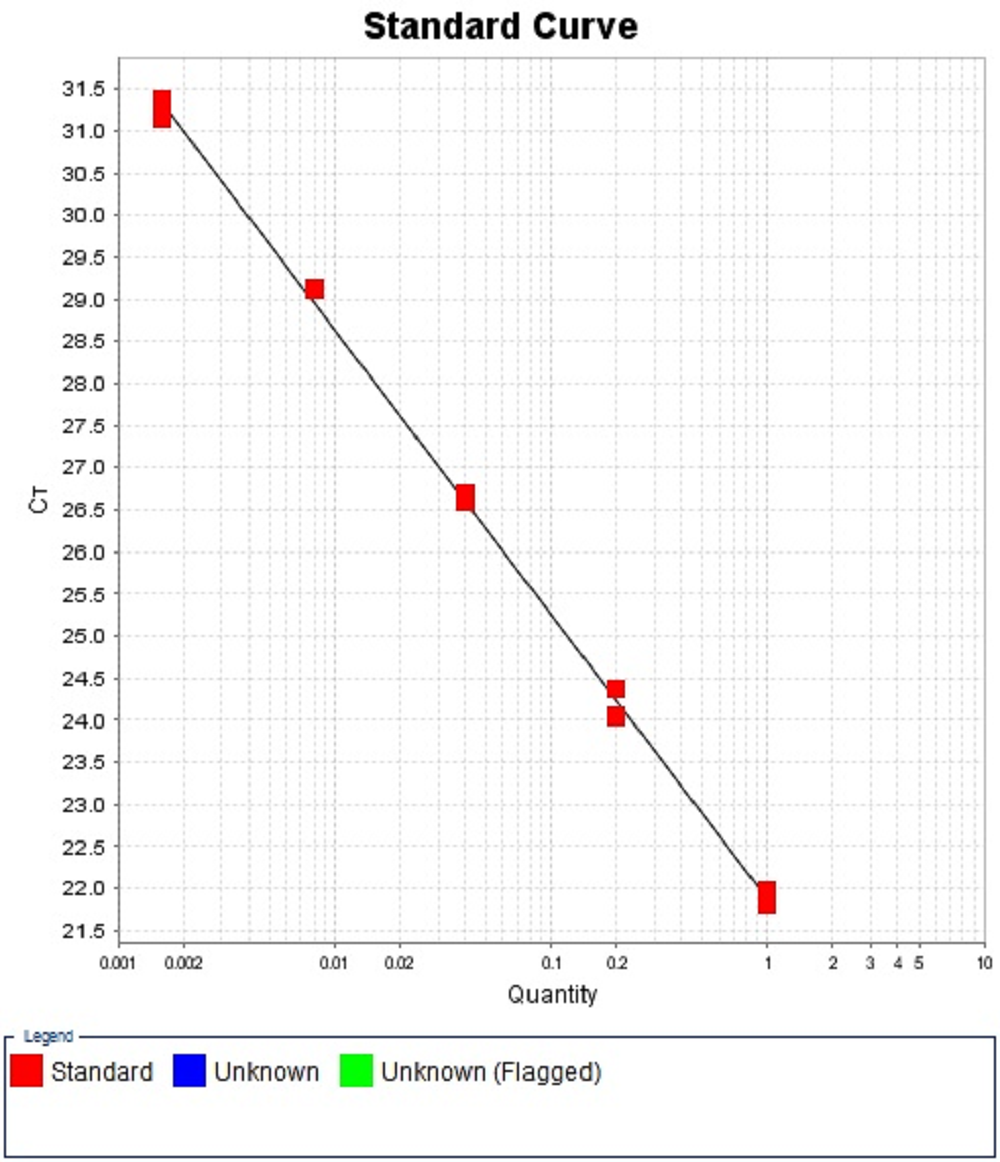 |
| ***Cyp 2*** | **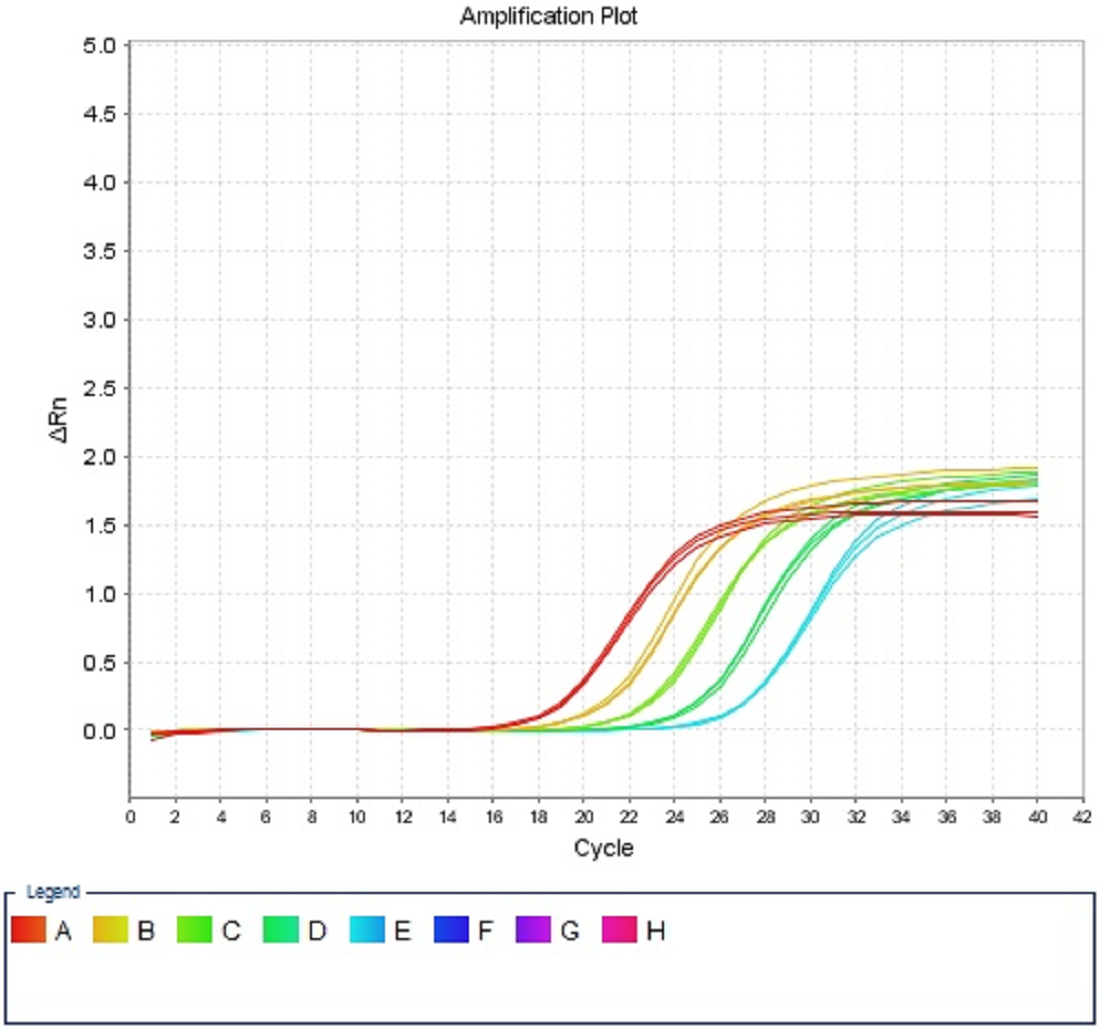** | **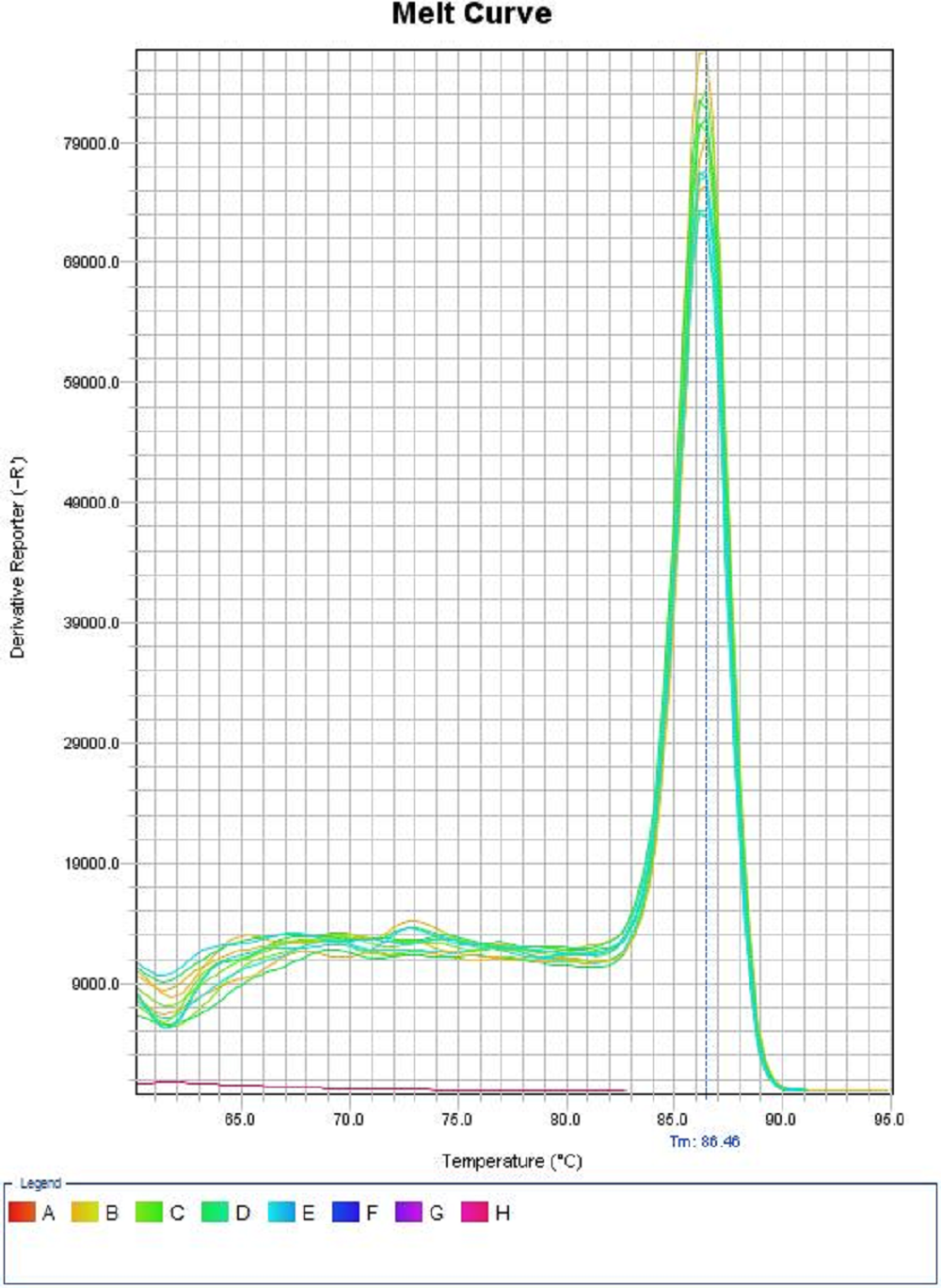** | **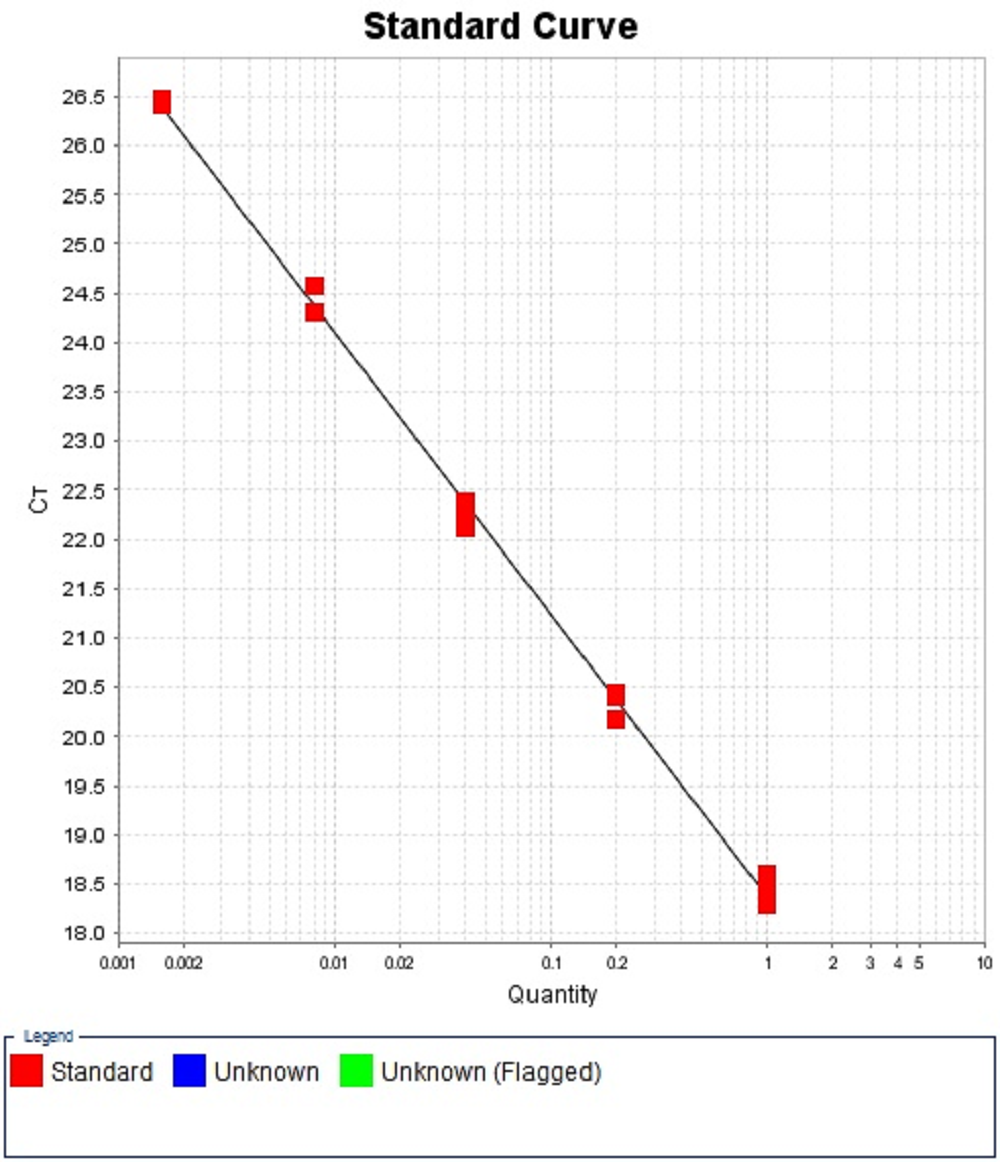** |
| ***EF-1α*** | **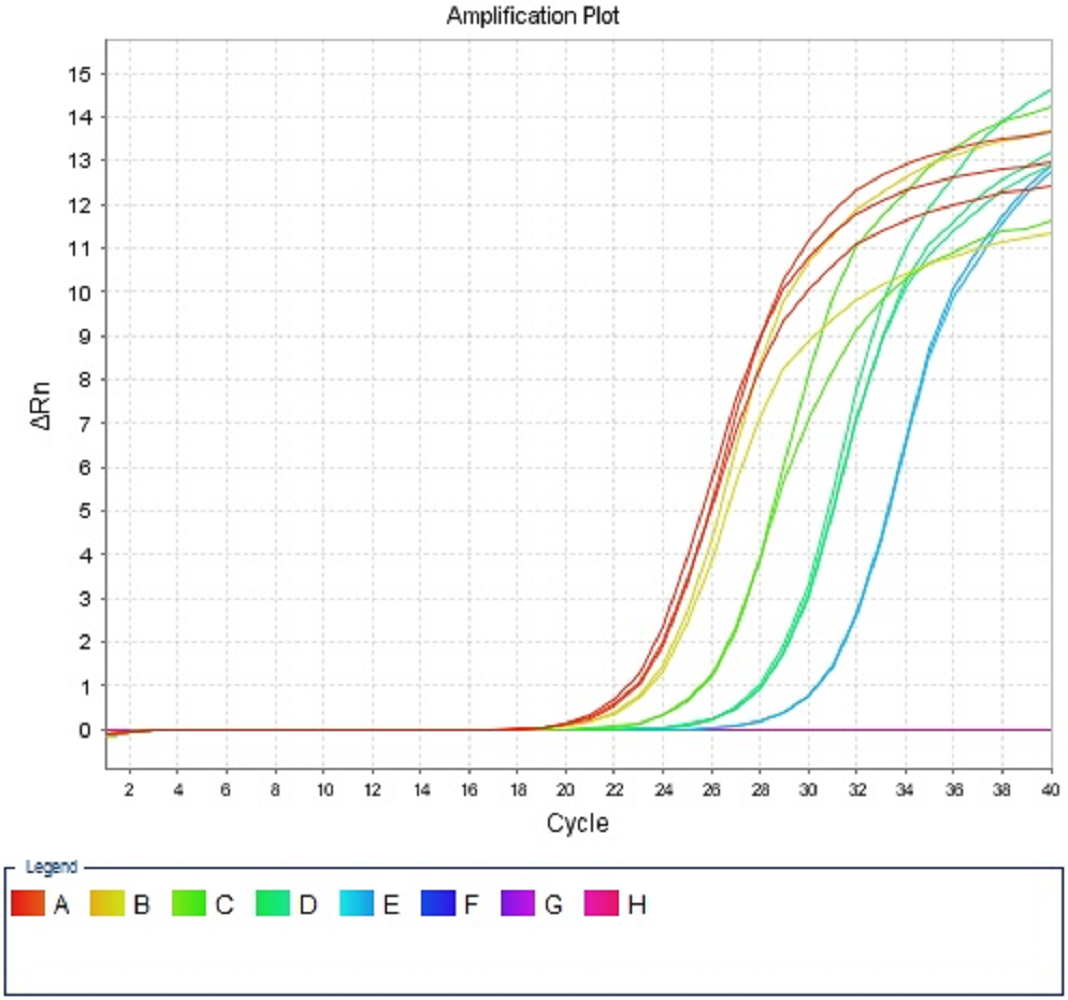** | 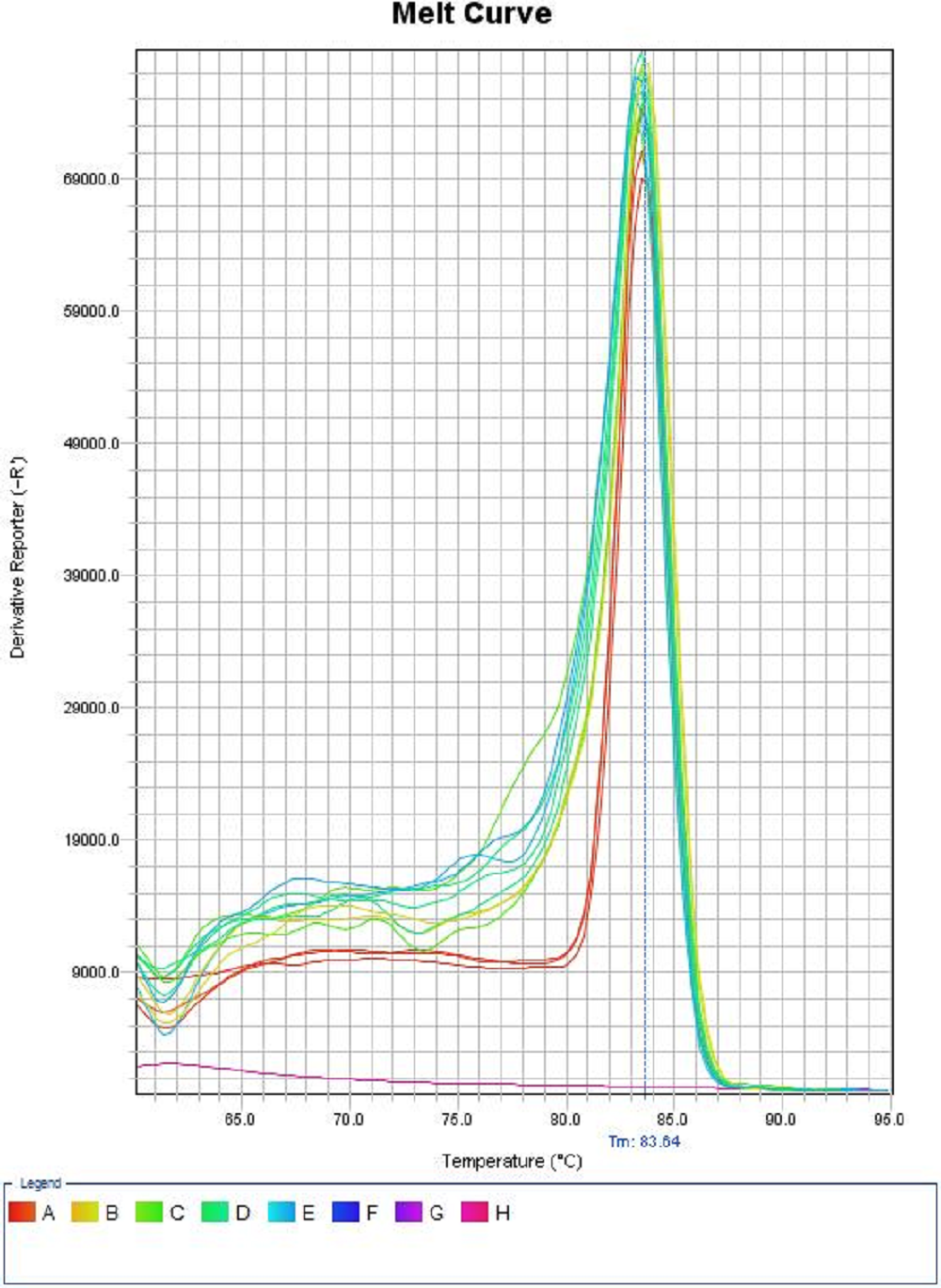 | 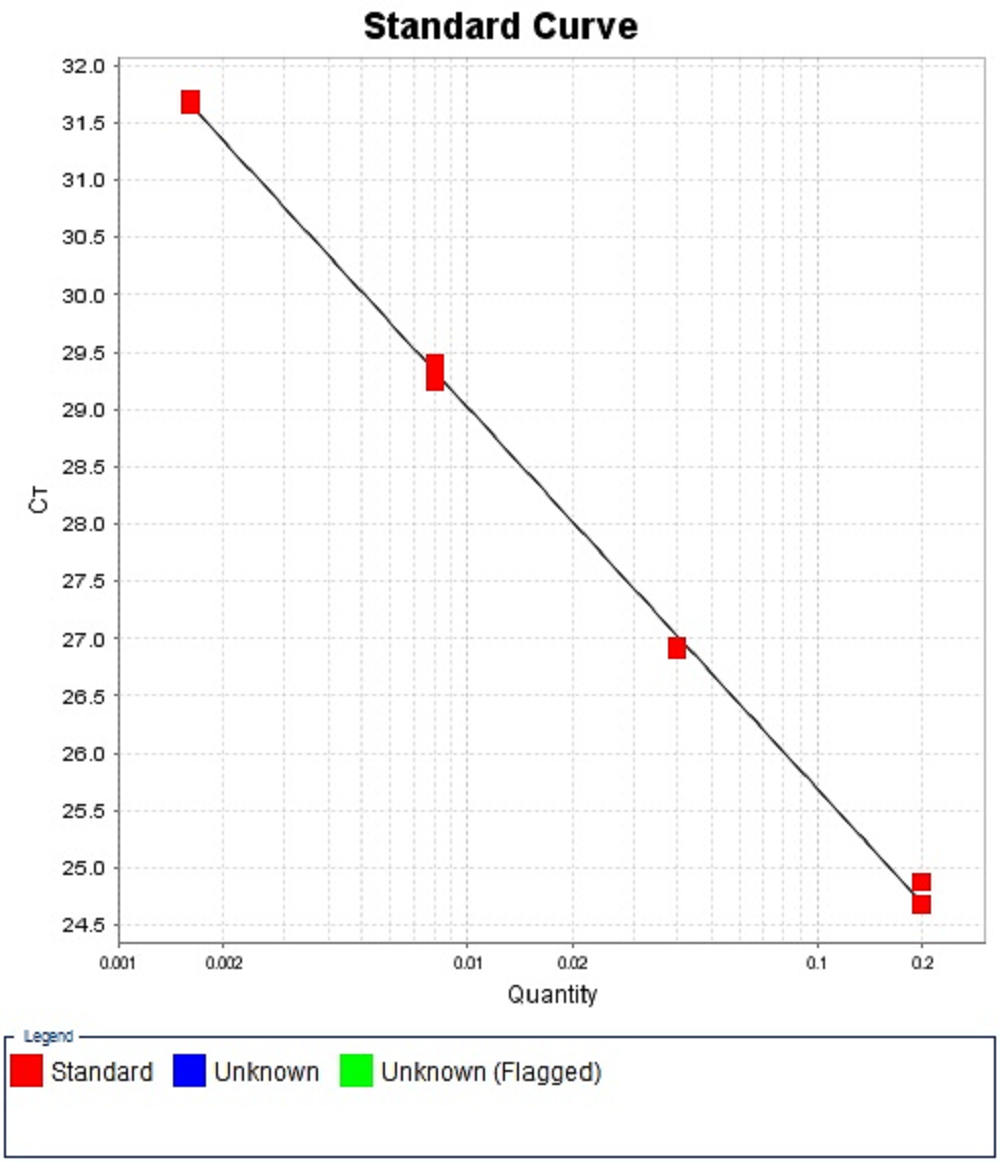 |
| ***F-box*** | **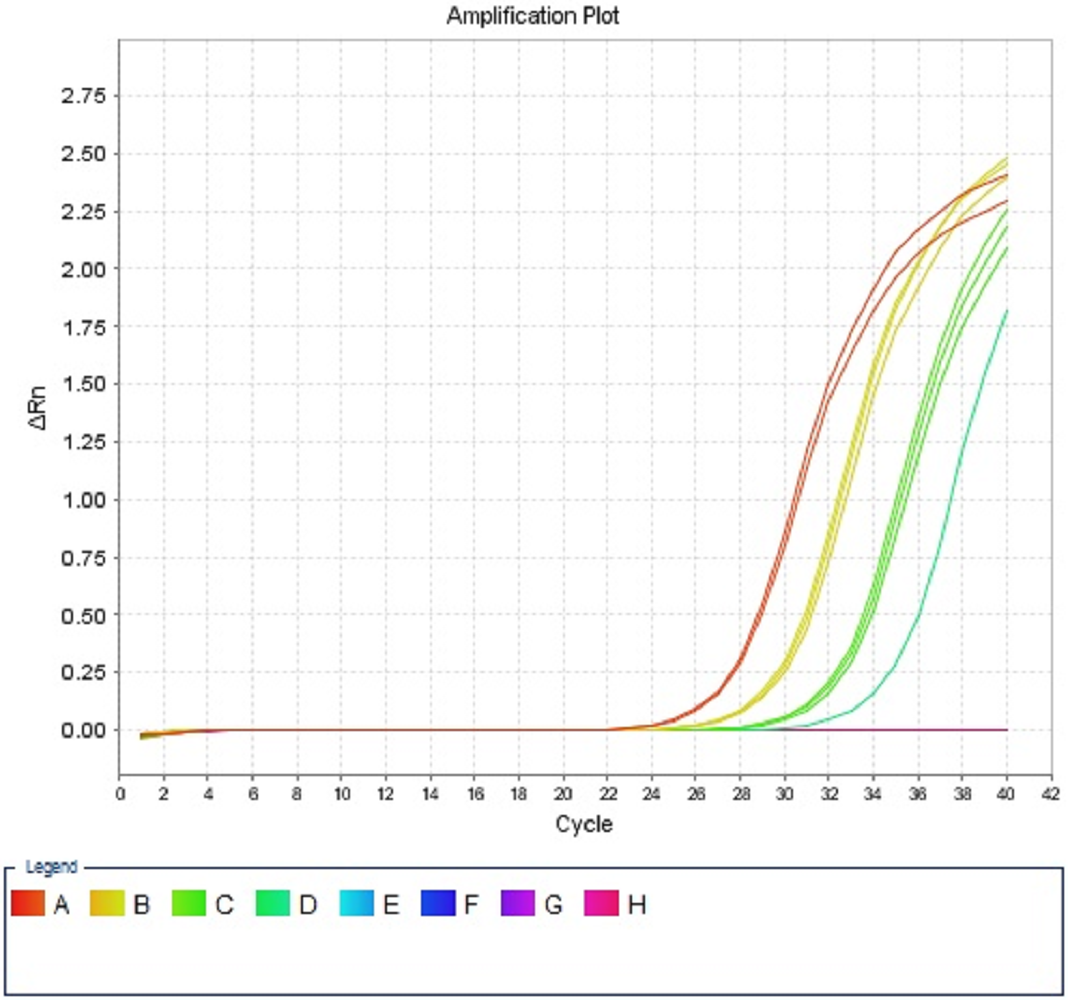** | **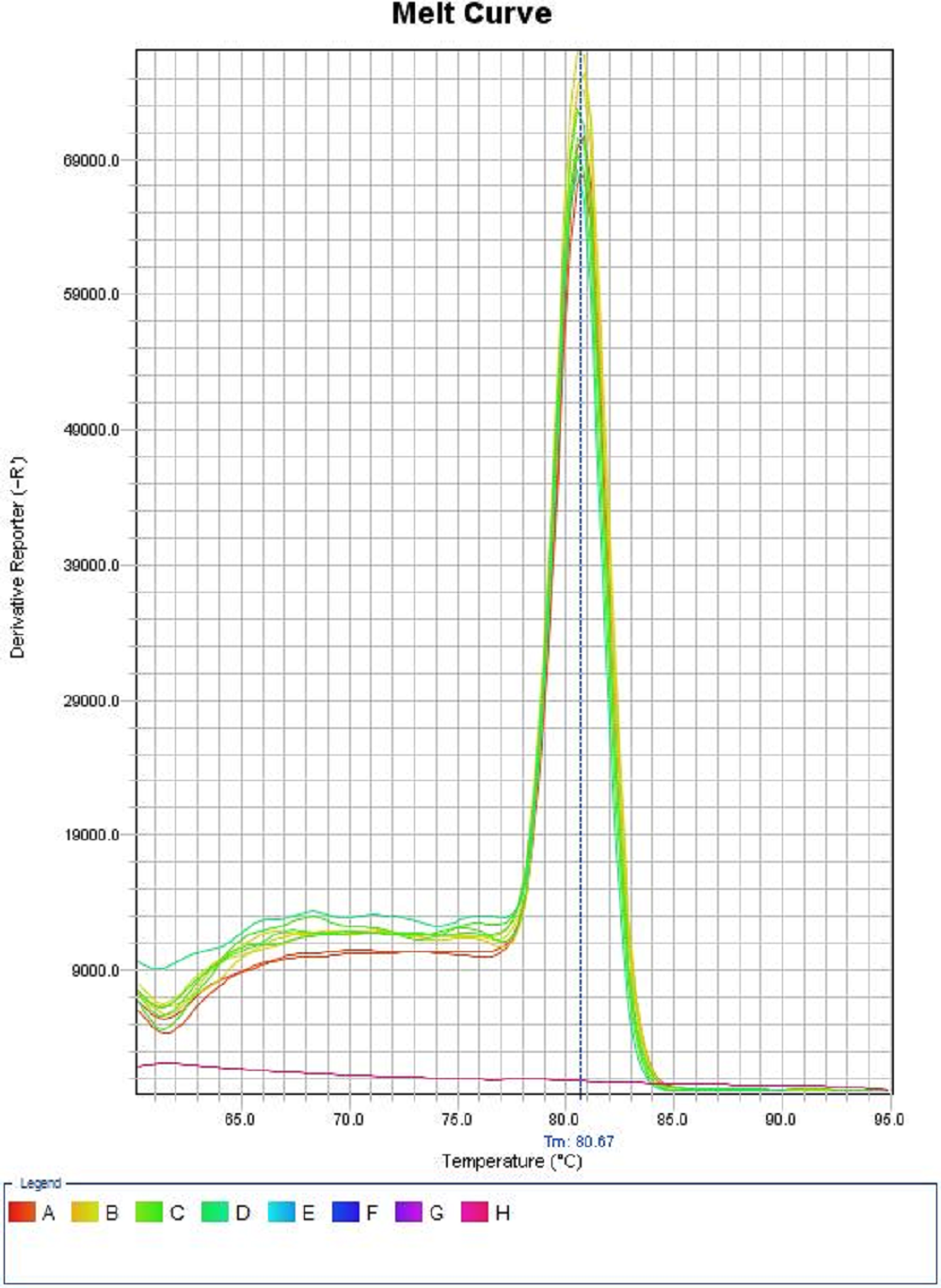** | **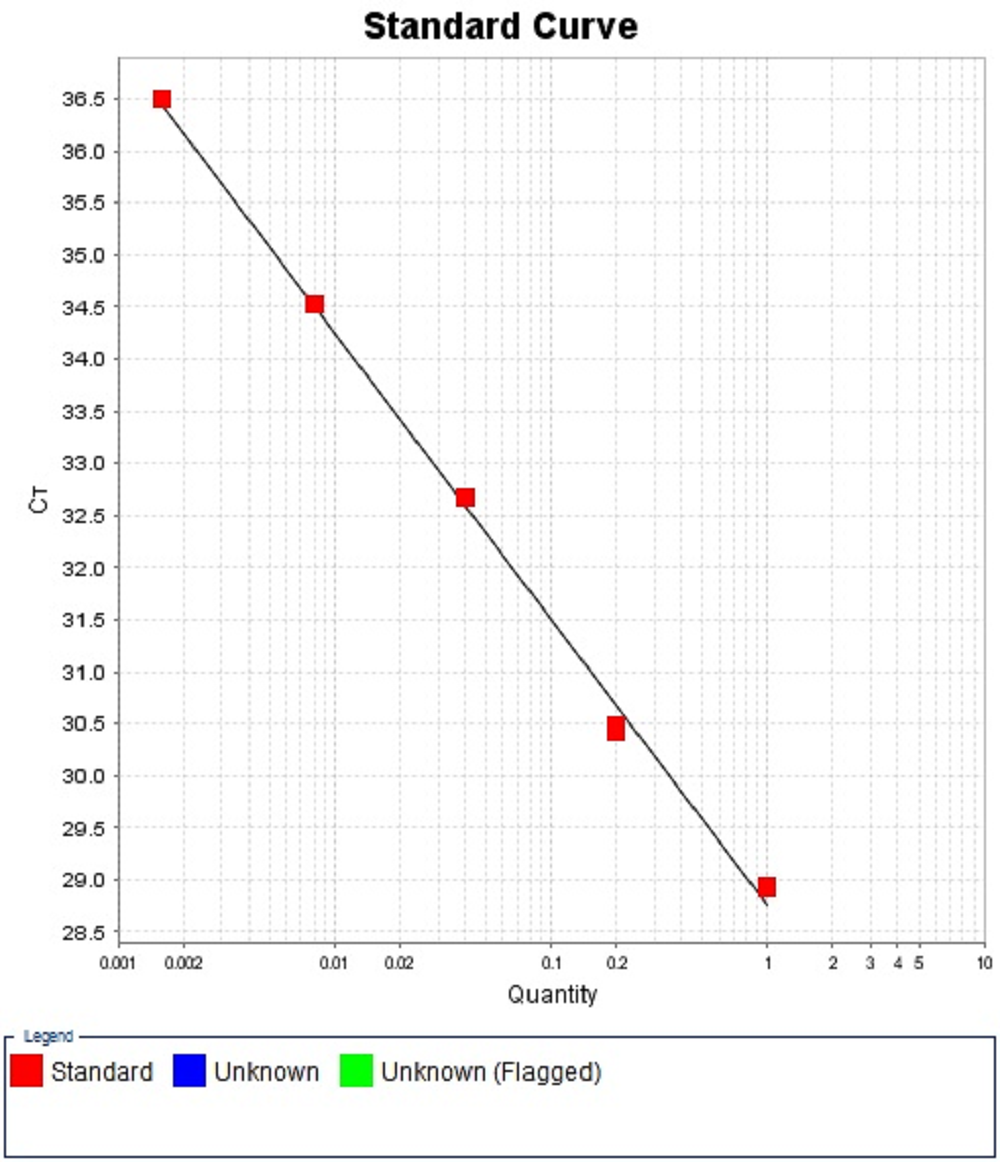** |
| ***GAPDH*** | **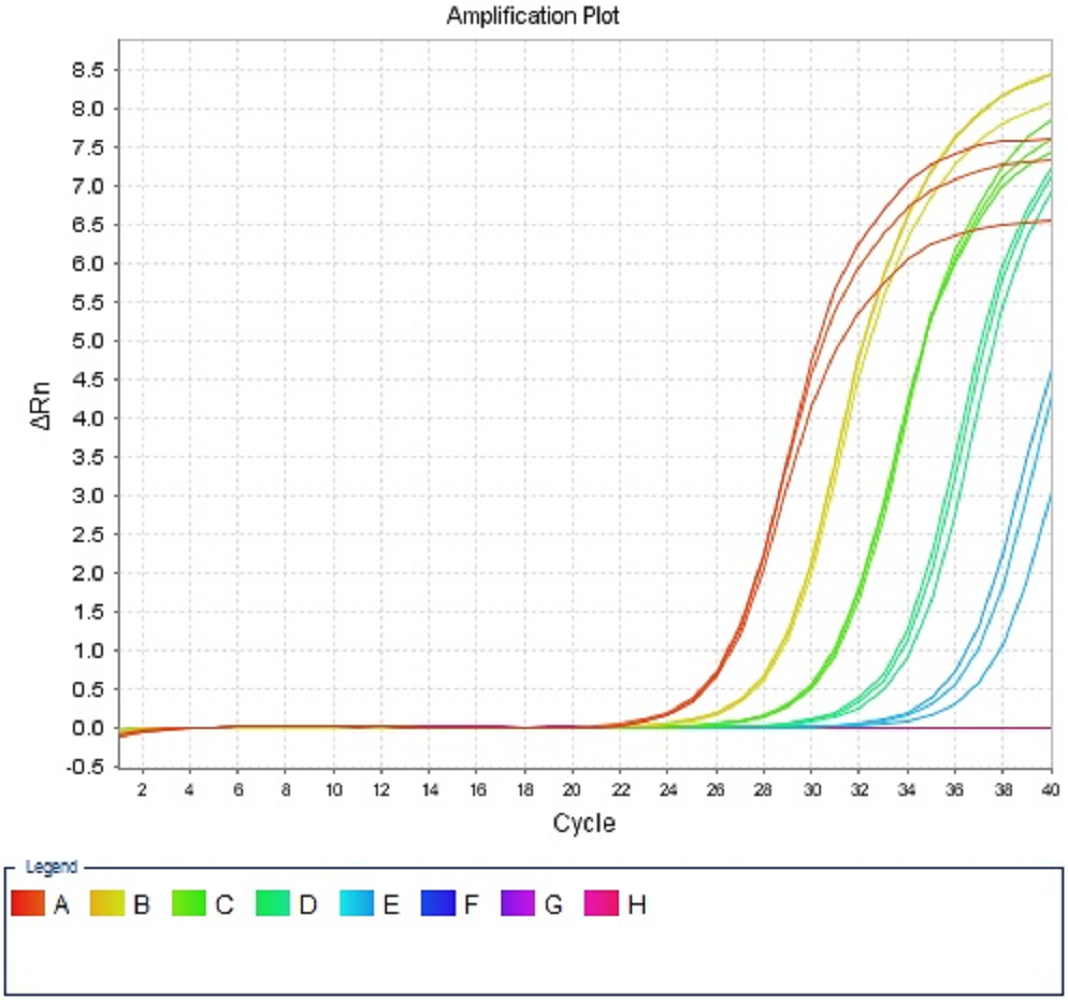** | **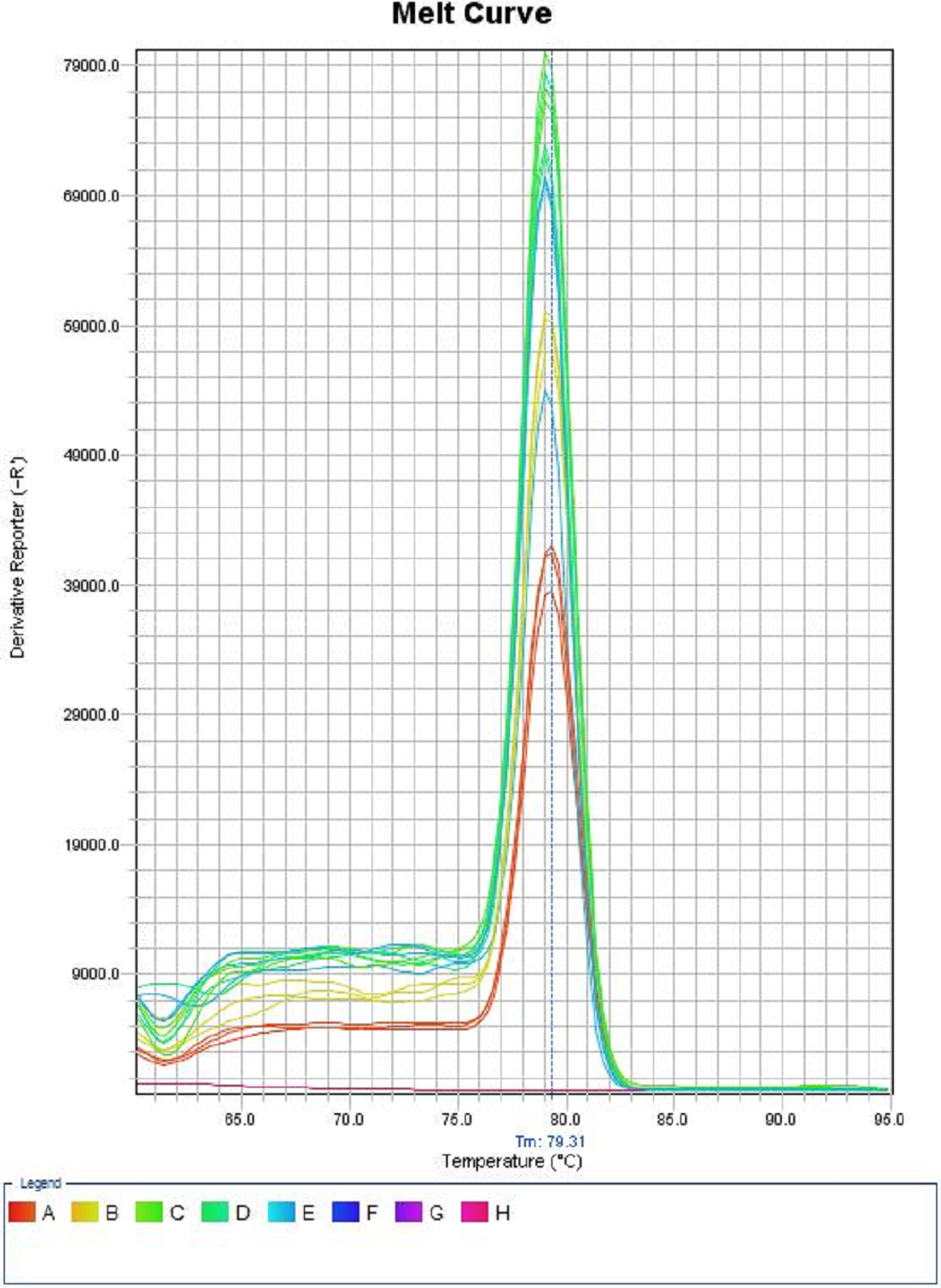** | **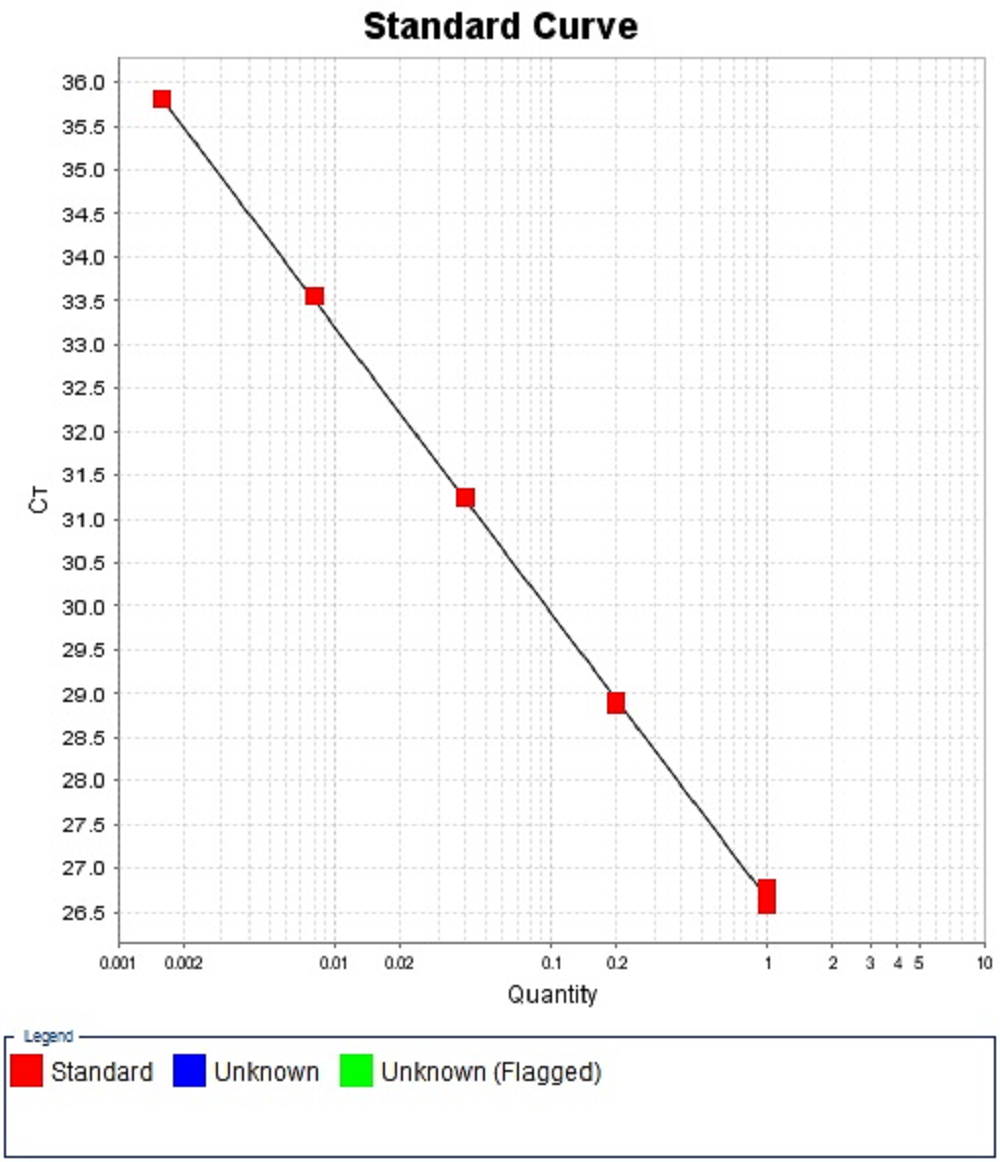** |
| ***18s rRNA*** | **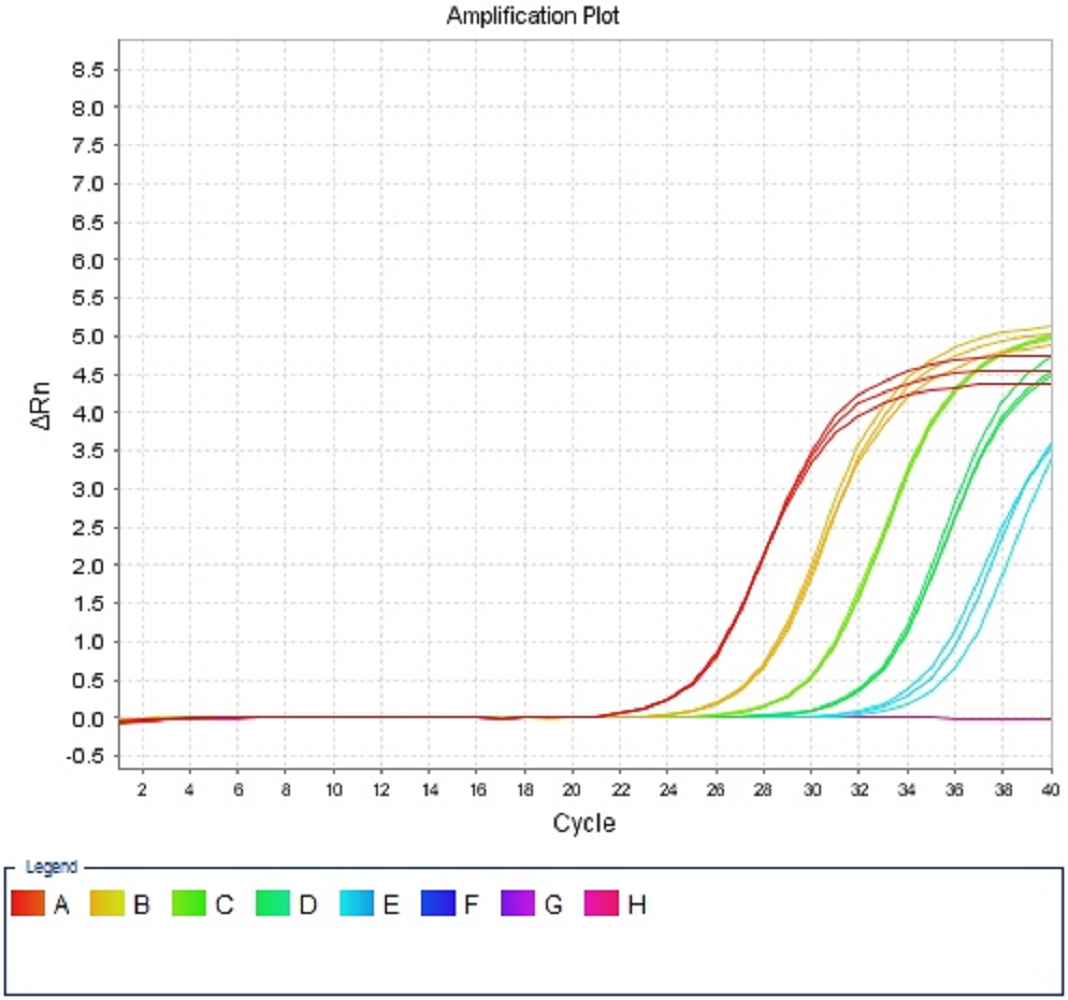** | **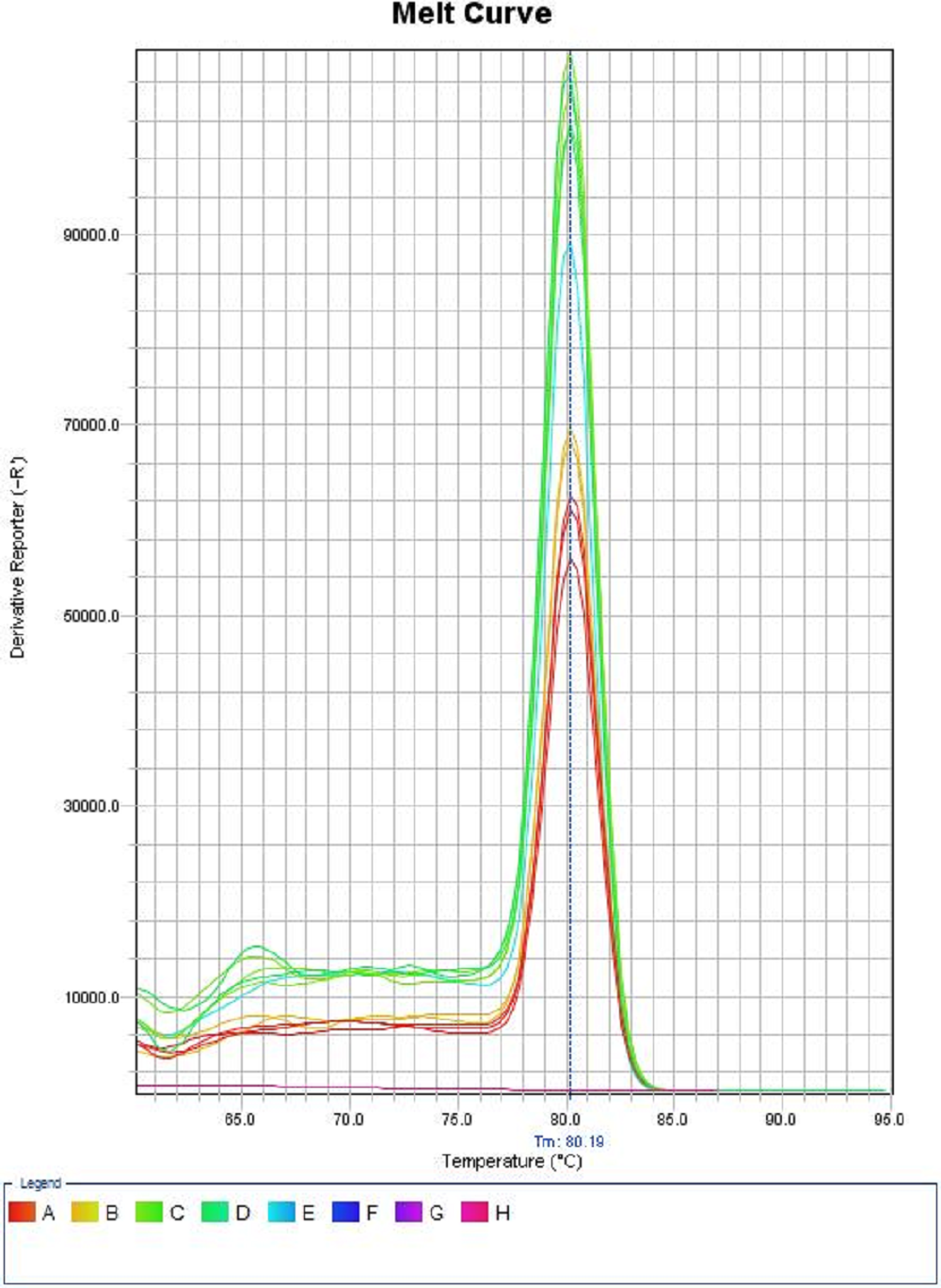** | **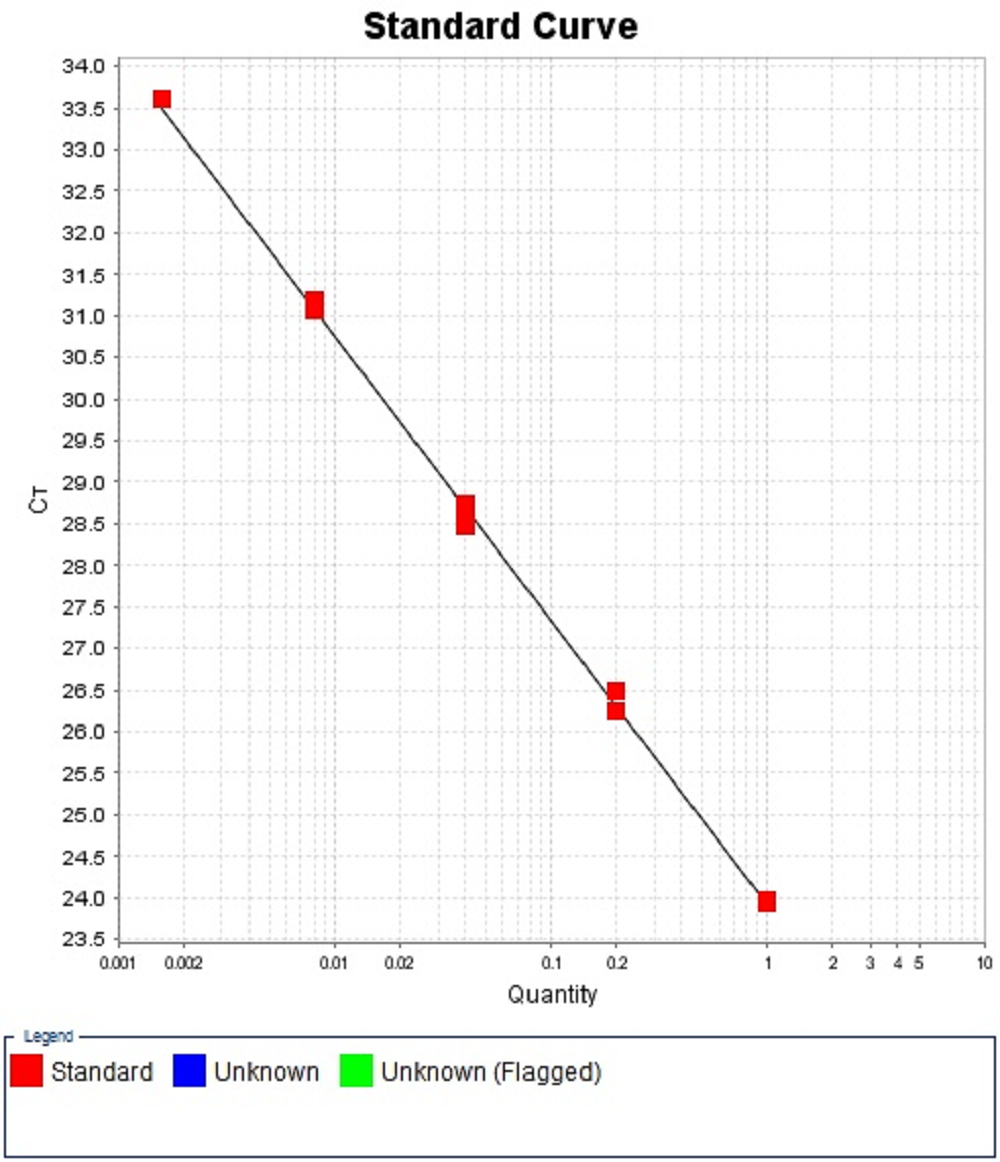** |
| ***TUBB*** | 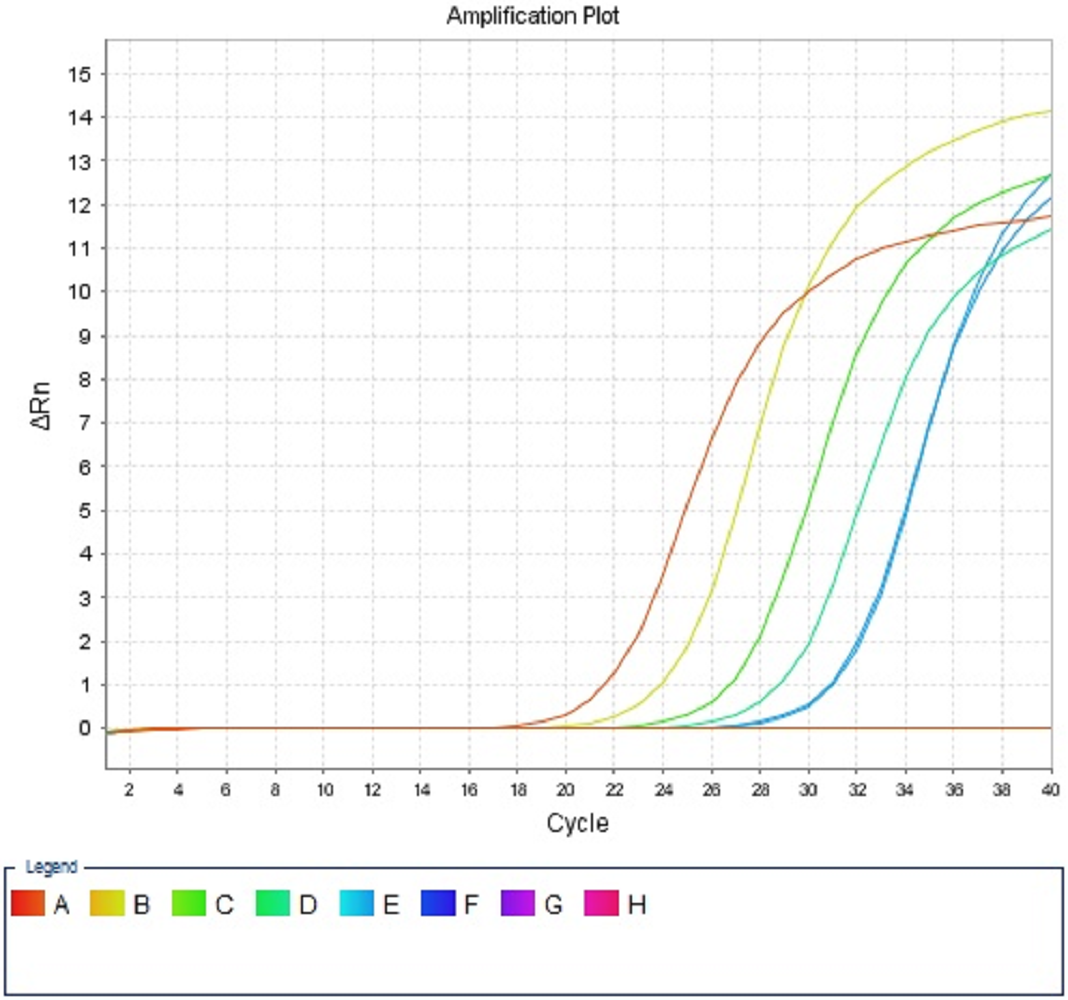 | 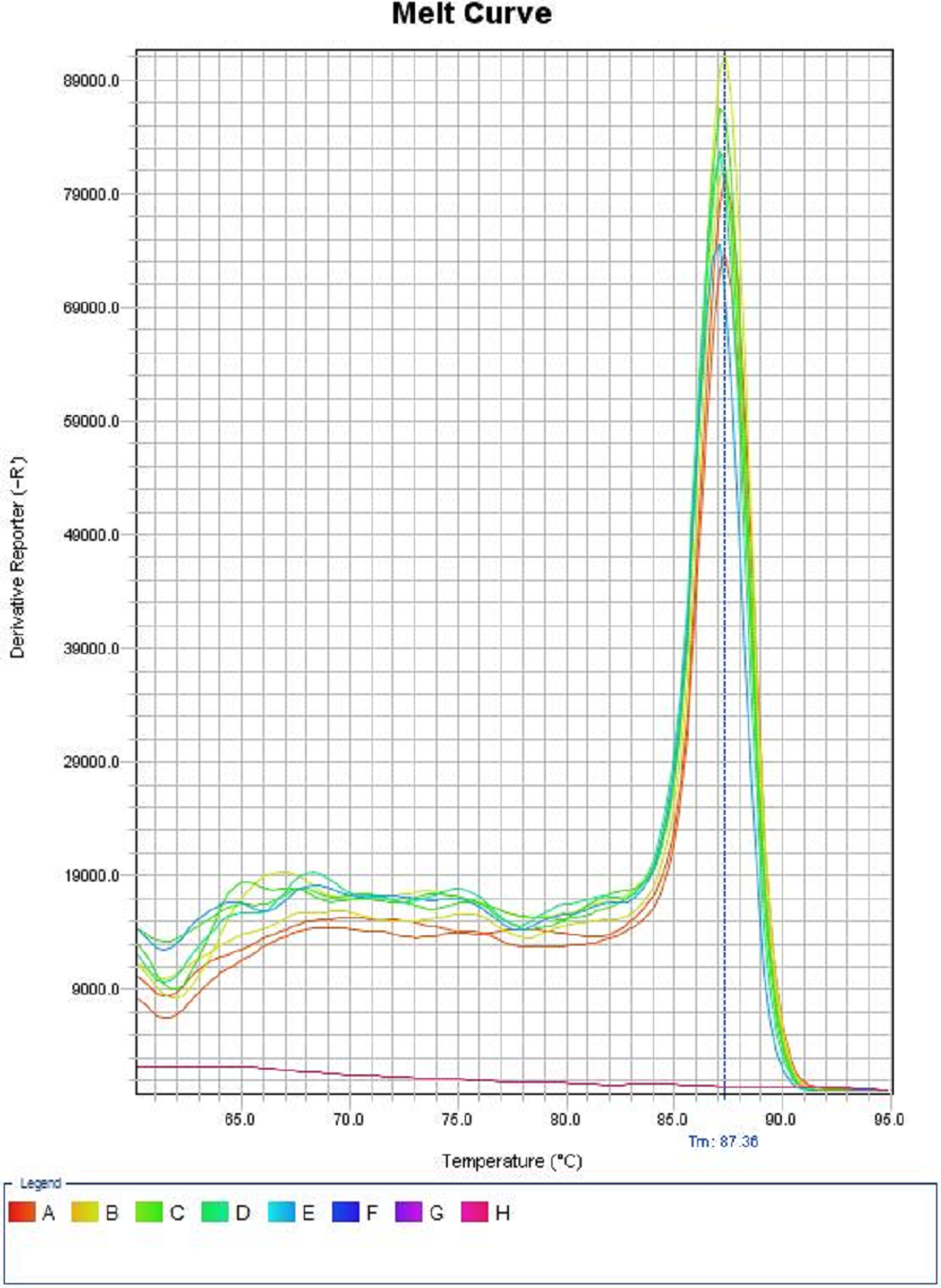 | 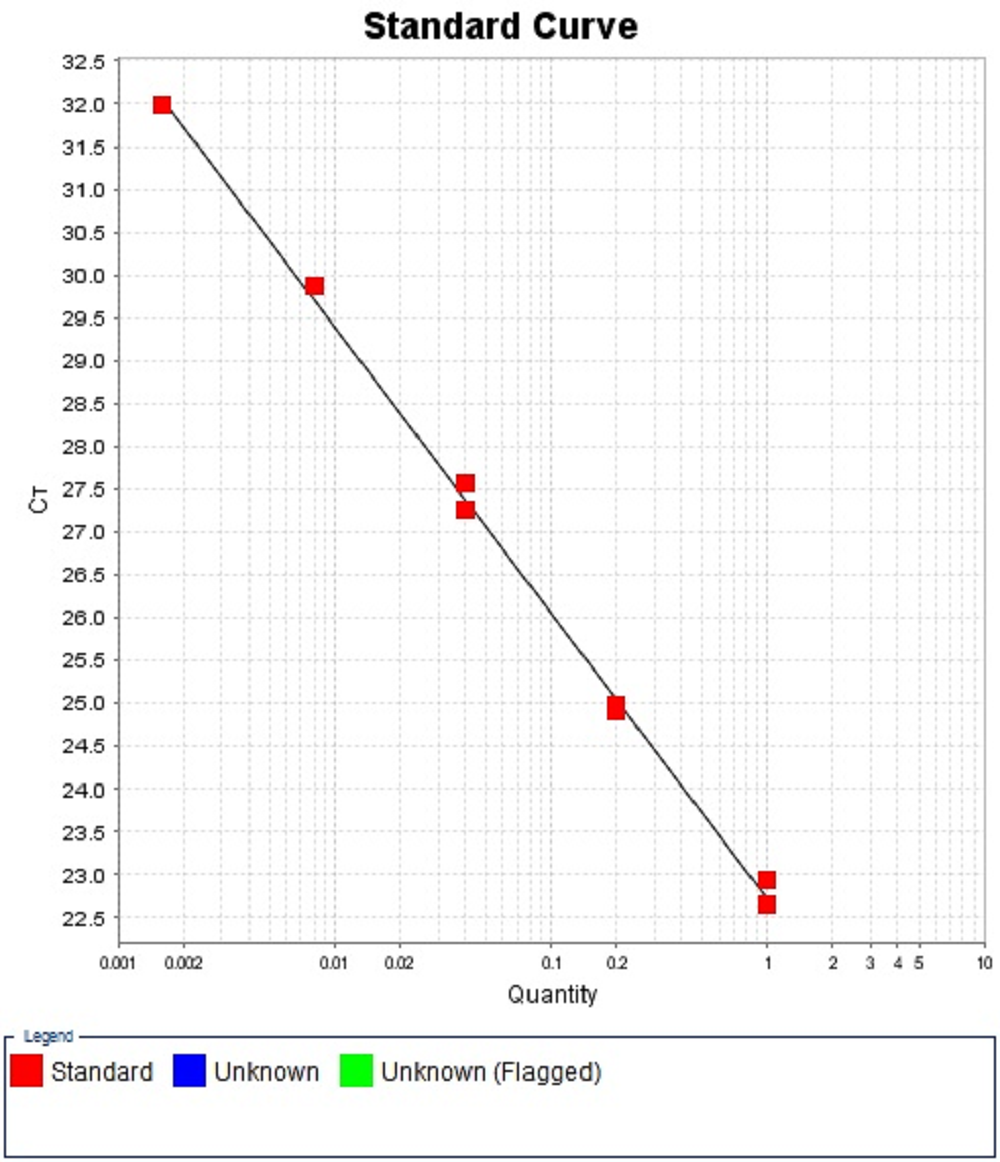 |
| ***PP2A*** | 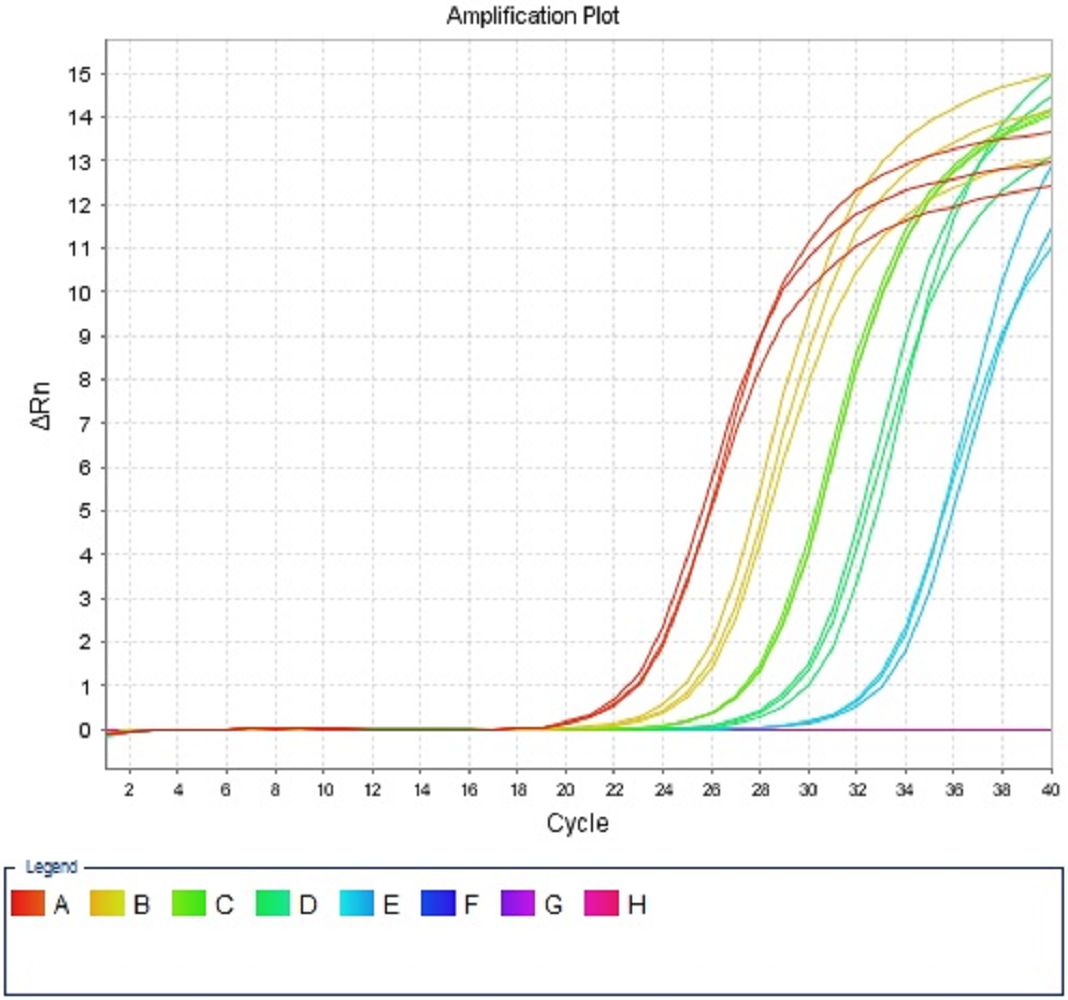 | 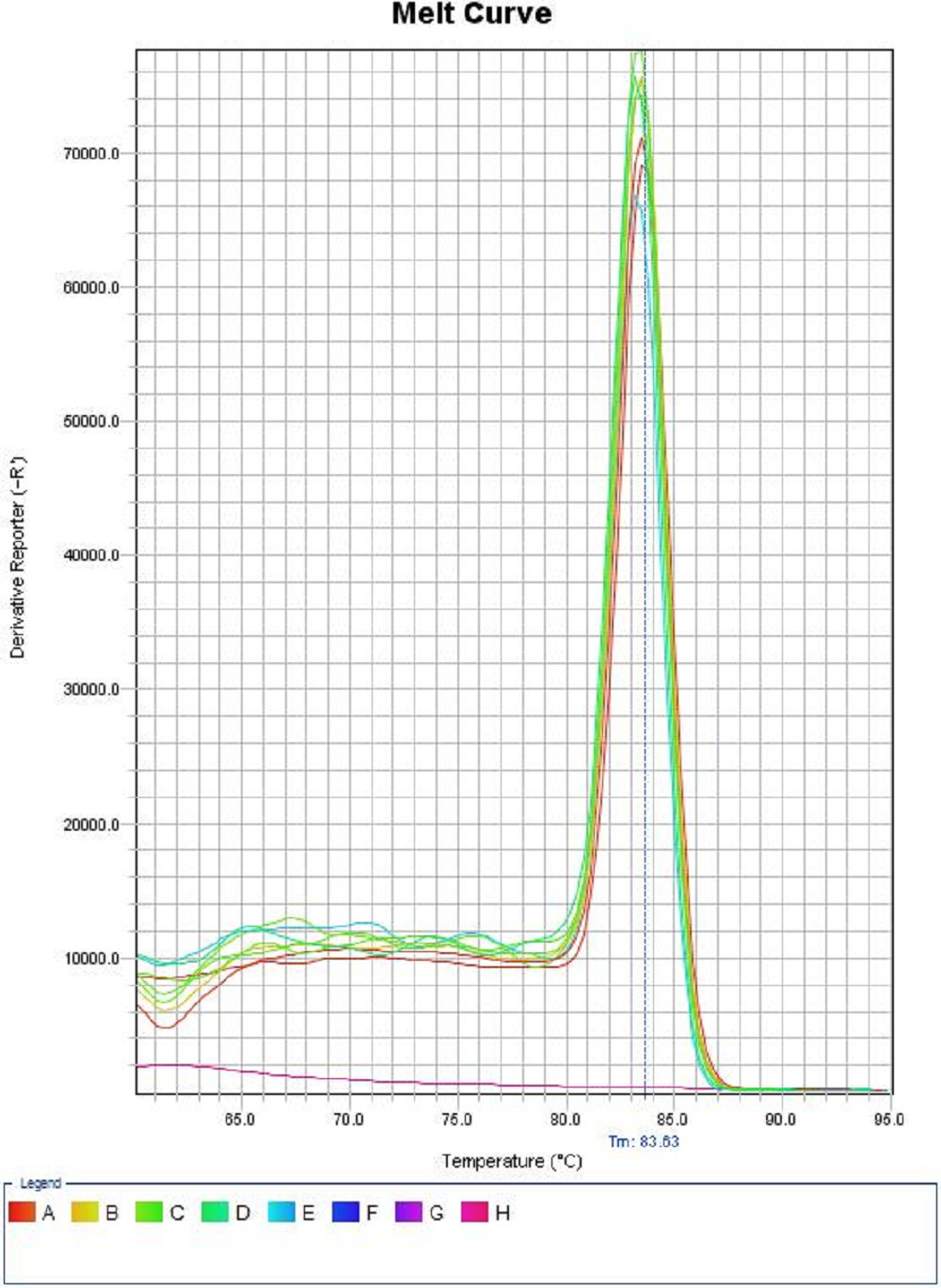 | 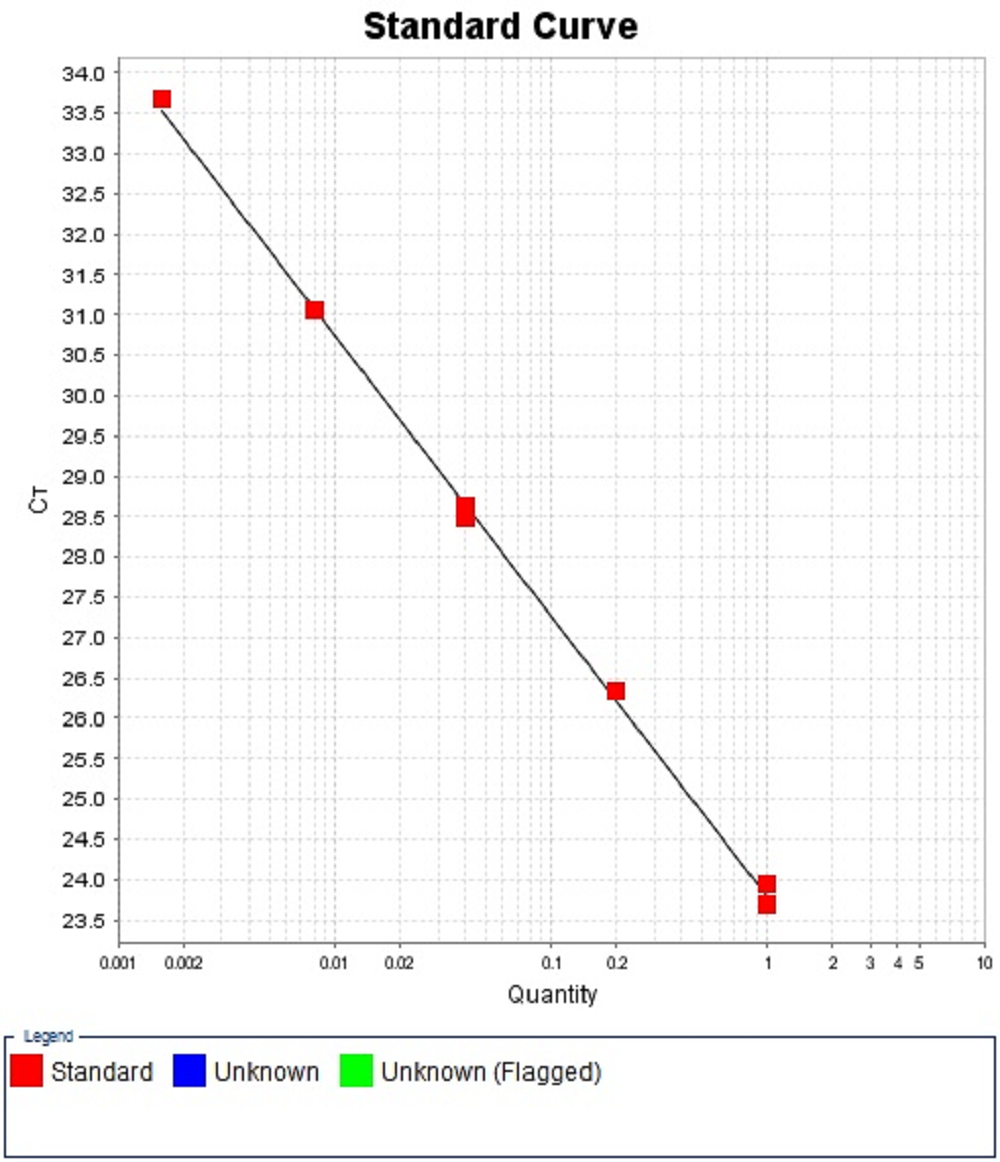 |
| ***SAND*** | **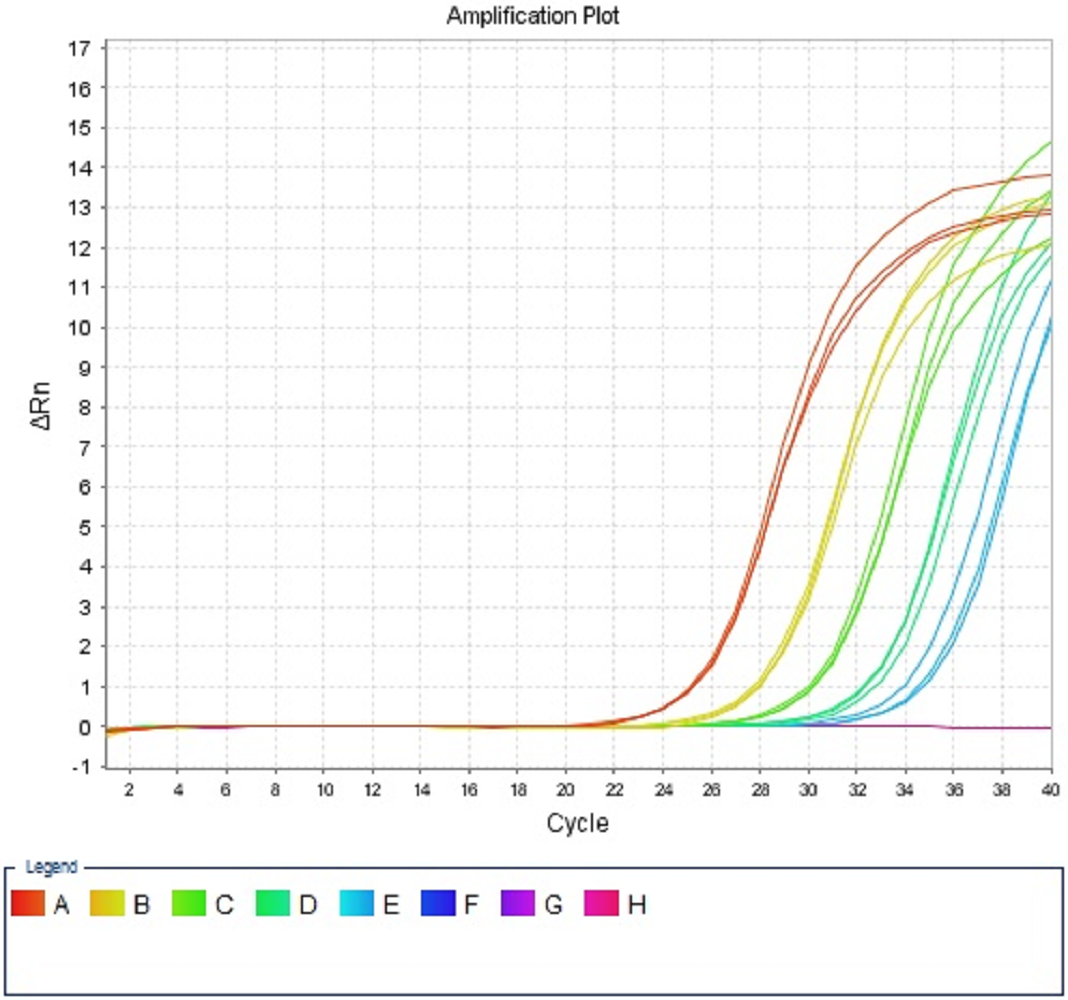** | **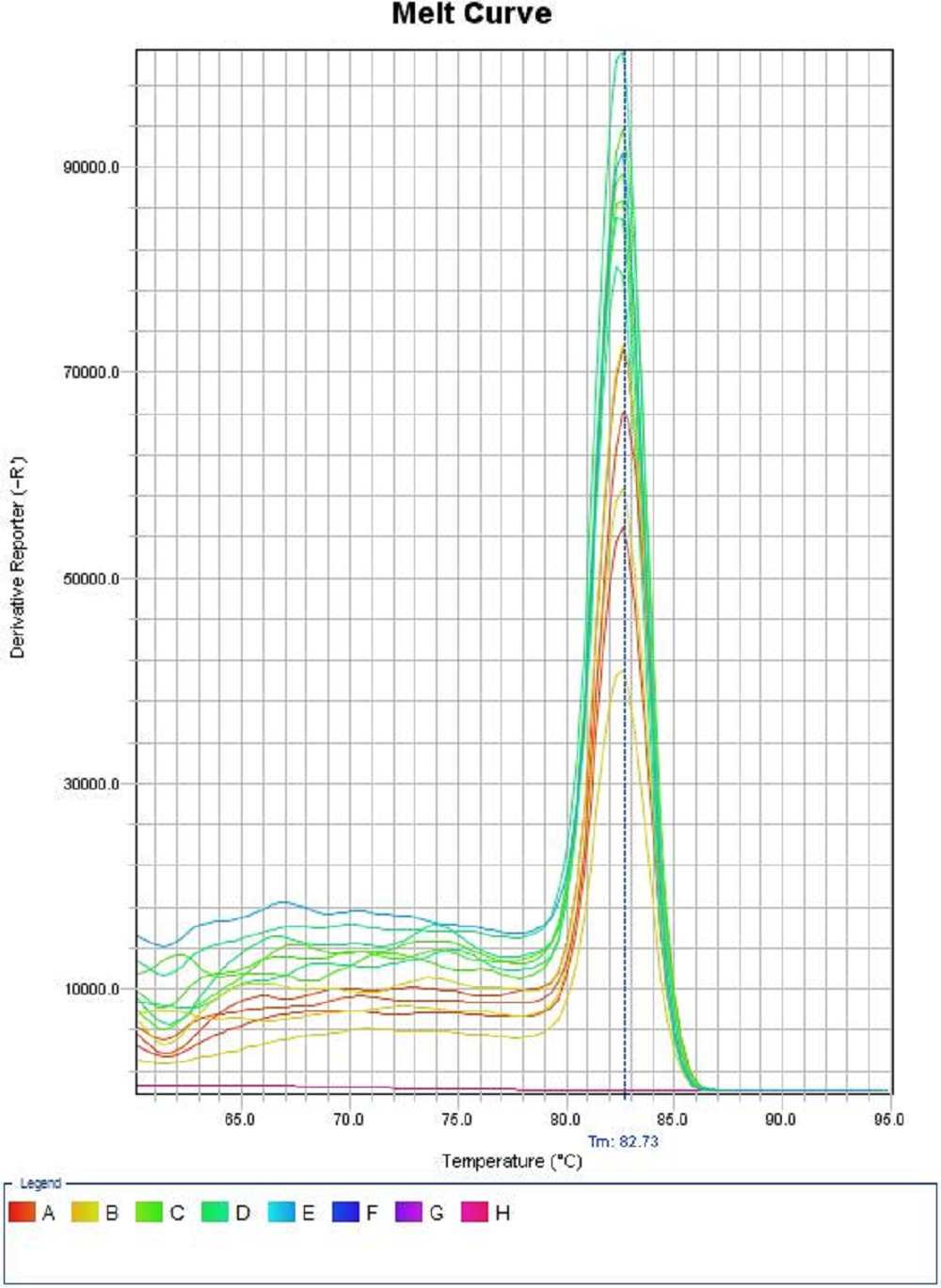** | **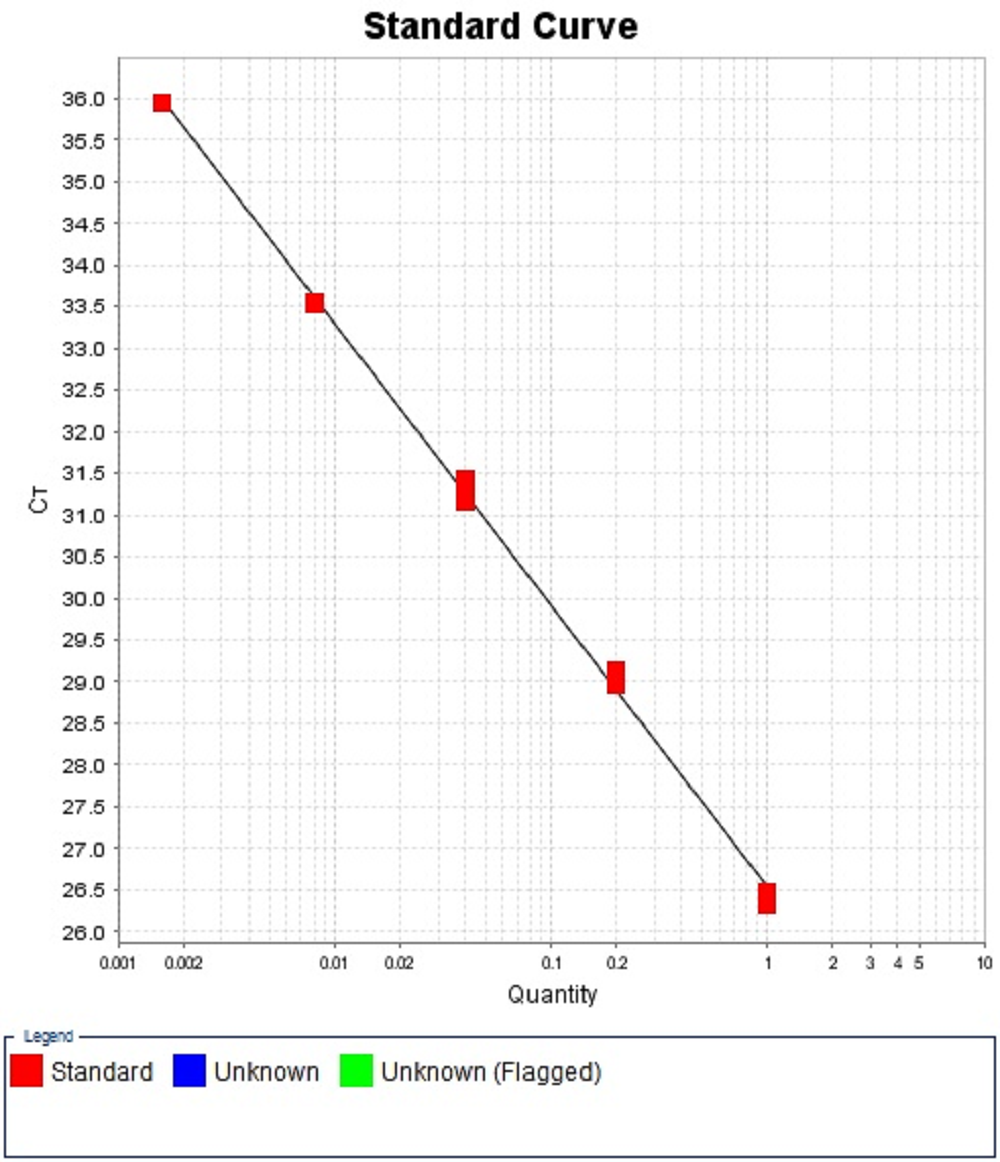** |
| ***RN 8*** | **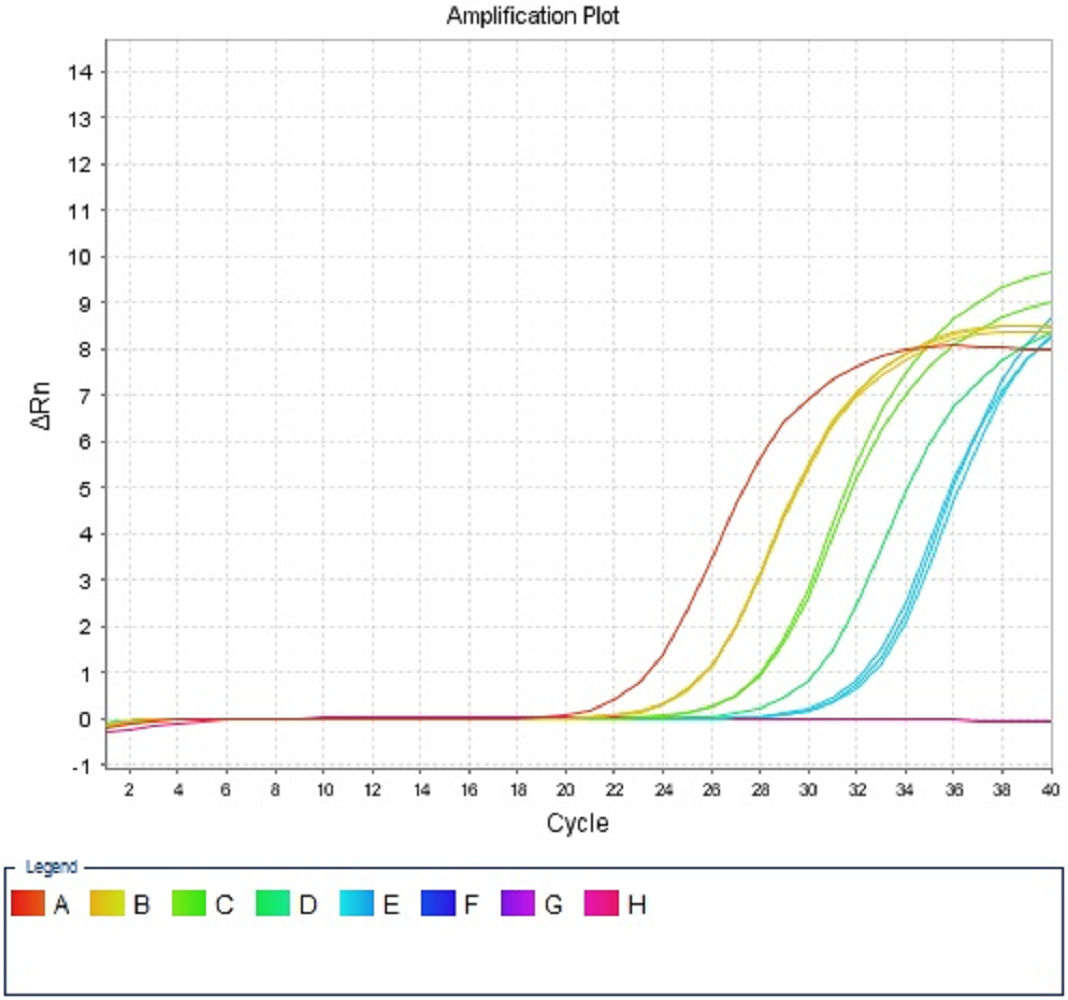** | **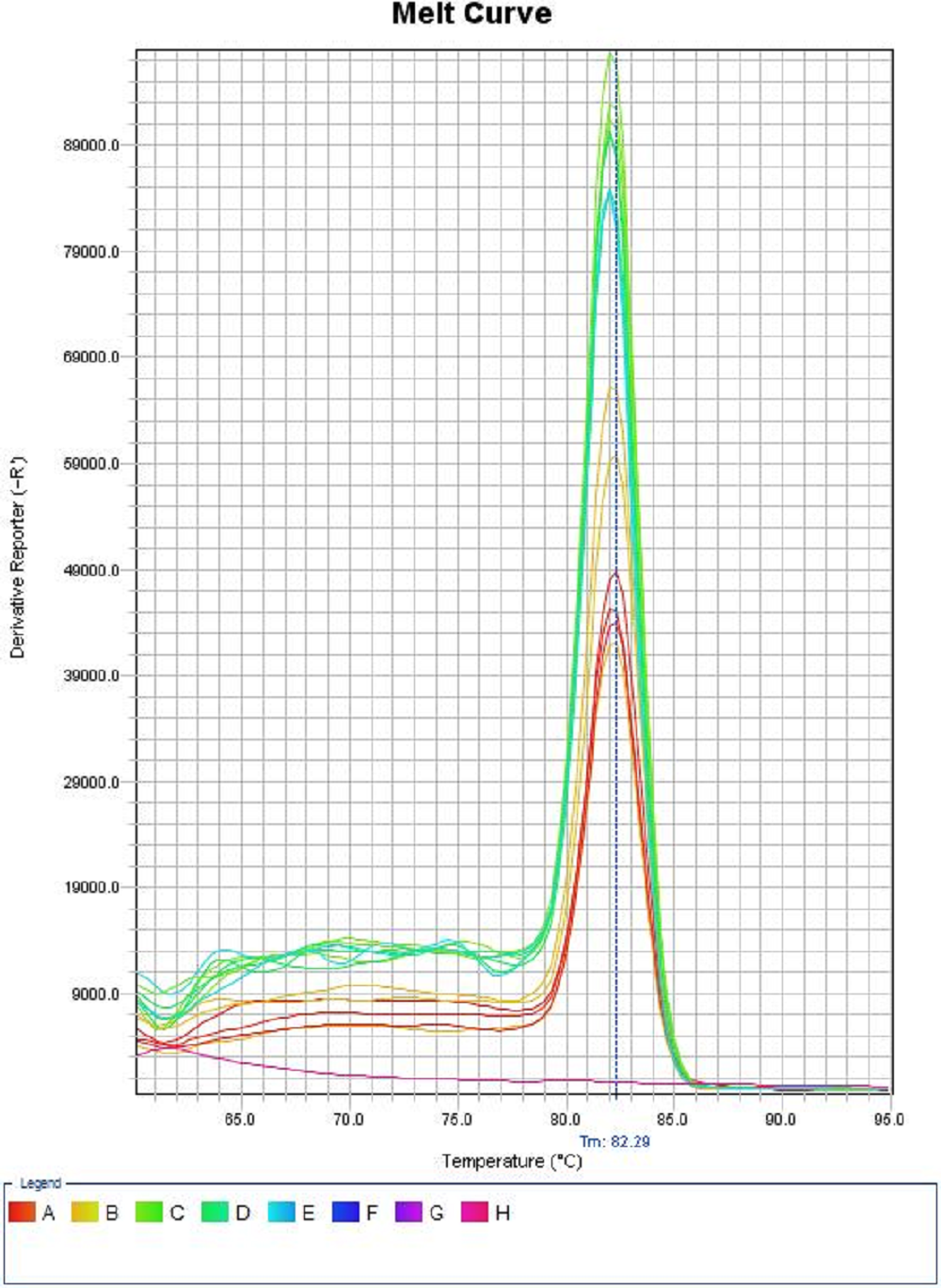** | **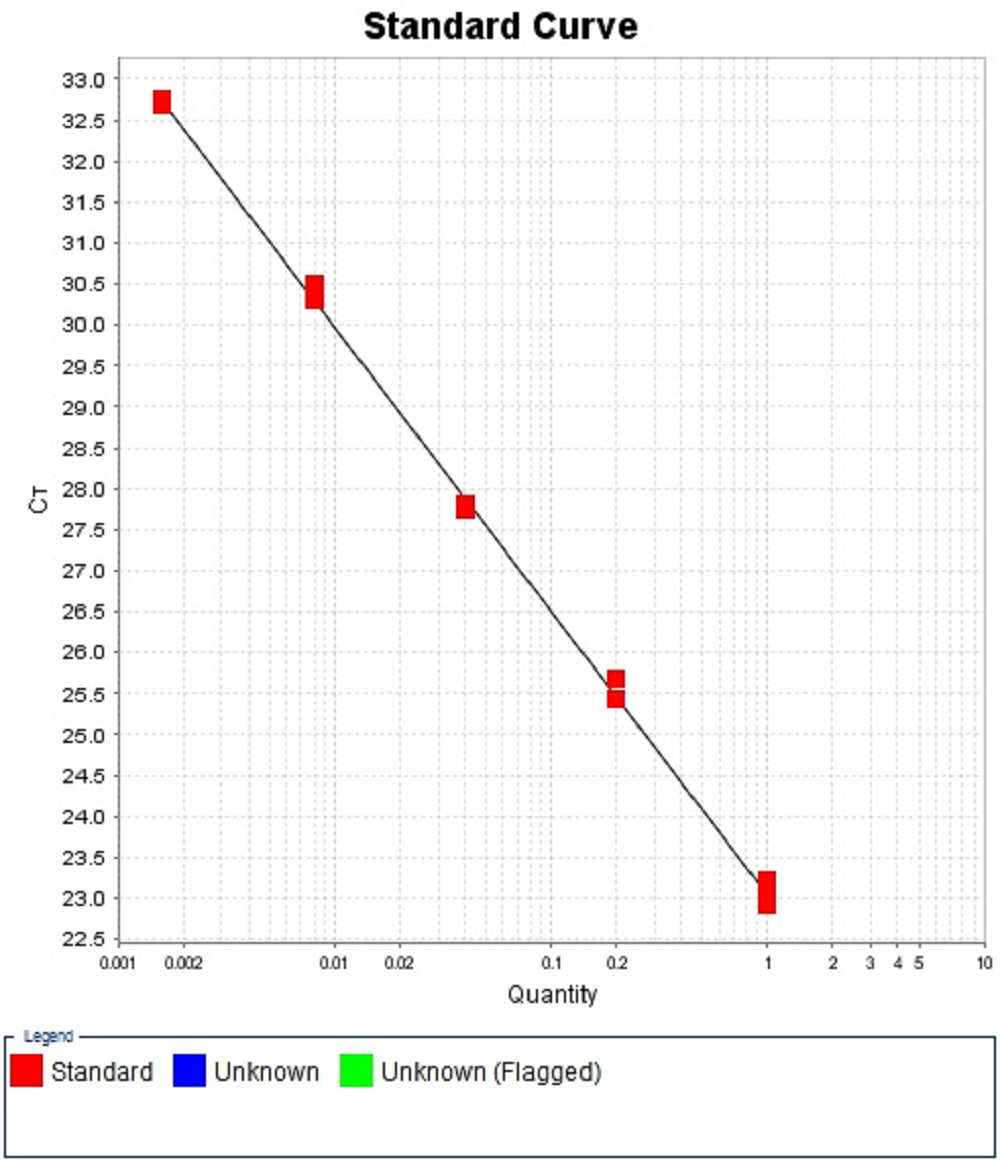** |
