# Supplementary material for: Selection of reference genes for normalization of cranberry (Vaccinium macrocarpon Ait.) gene expression under different experimental conditions
Source: PLoS One. 2019 Nov 12;14(11):e0224798. doi: 10.1371/journal.pone.0224798 (PMC6850891; doi:10.1371/journal.pone.0224798)
Supplement: S3 Table — (DOC) [file pone.0224798.s006.doc]

**Table S3. Description of the samples used for qRT-PCR, and the 5 different combinations of sample sets.**

| **Different experimental conditions** | **Abiotic Stress Conditions** | **Sample No.** | **Cultivar** | **Treated time** | | **Tissue** | **The number of technical replicates** | **The number of biological replicates** |
| --- | --- | --- | --- | --- | --- | --- | --- | --- |
|  |  |  |  |  |  |  |  |  |
|  |  |  |  |  |  |  |  |  |
|  |  |  |  |  |  |  |  |  |
| **Abiotic stress treatments** | salt stress treatment (200mmol/L NaCl) | 1 | Bain 11 | 3 hours | | leaves | 3 | 3 |
|  |  | 2 | Bain 11 | 6 hours | | leaves | 3 | 3 |
|  |  | 3 | Bain 11 | 9 hours | | leaves | 3 | 3 |
|  |  | 4 | Bain 11 | 12 hours | | leaves | 3 | 3 |
|  |  | 5 | Bain 11 | 3 hours | | roots | 3 | 3 |
|  |  | 6 | Bain 11 | 6 hours | | roots | 3 | 3 |
|  |  | 7 | Bain 11 | 9 hours | | roots | 3 | 3 |
|  |  | 8 | Bain 11 | 12 hours | | roots | 3 | 3 |
|  | alkali stress treatment (200mmol/L NaHCO_3_) | 9 | Bain 11 | 3 hours | | leaves | 3 | 3 |
|  |  | 10 | Bain 11 | 6 hours | | leaves | 3 | 3 |
|  |  | 11 | Bain 11 | 9 hours | | leaves | 3 | 3 |
|  |  | 12 | Bain 11 | 12 hours | | leaves | 3 | 3 |
|  |  | 13 | Bain 11 | 3 hours | | roots | 3 | 3 |
|  |  | 14 | Bain 11 | 6 hours | | roots | 3 | 3 |
|  |  | 15 | Bain 11 | 9 hours | | roots | 3 | 3 |
|  |  | 16 | Bain 11 | 12 hours | | roots | 3 | 3 |
|  | PEG simulated drought stress treatment (8%PEG8000) | 17 | Bain 11 | 3 hours | | leaves | 3 | 3 |
|  |  | 18 | Bain 11 | 6 hours | | leaves | 3 | 3 |
|  |  | 19 | Bain 11 | 9 hours | | leaves | 3 | 3 |
|  |  | 20 | Bain 11 | 12 hours | | leaves | 3 | 3 |
|  |  | 21 | Bain 11 | 3 hours | | roots | 3 | 3 |
|  |  | 22 | Bain 11 | 6 hours | | roots | 3 | 3 |
|  |  | 23 | Bain 11 | 9 hours | | roots | 3 | 3 |
|  |  | 24 | Bain 11 | 12 hours | | roots | 3 | 3 |
|  | control | 25 | Bain 11 | 0 hours | | leaves | 3 | 3 |
|  |  | 26 | Bain 11 | 0 hours | | roots | 3 | 3 |
| **Different cultivars** |  | 27 | Brewer |  | | leaves | 3 | 3 |
|  |  | 28 | Bain Fav.No.1 |  |  | leaves | 3 | 3 |
|  |  | 29 | Bain 11 |  |  | leaves | 3 | 3 |
|  |  | 30 | Hollister Red |  |  | leaves | 3 | 3 |
|  |  | 31 | Bain 6 |  |  | leaves | 3 | 3 |
|  |  | 32 | Washington |  |  | leaves | 3 | 3 |
| **Different organs** |  | 33 | Bain 11 |  | | roots | 3 | 3 |
|  |  | 34 | Bain 11 |  |  | stems | 3 | 3 |
|  |  | 35 | Bain 11 |  |  | leaves | 3 | 3 |
|  |  | 36 | Bain 11 |  |  | flowers | 3 | 3 |
|  |  | 37 | Bain 11 |  |  | fruits | 3 | 3 |
|  |  | 38 | Bain 11 |  |  | seeds | 3 | 3 |
| **The five different combinations of sample sets.** | | | | | | | | |
| **Item** | **Sample sets** | | | | **samples** | | | |
| **A** | Different cultivars | | | | Samples of 27-32 | | | |
| **B** | Different organs | | | | Samples of 33-38 | | | |
| **C** | Leaves treated with three abiotic stresses | | | | Samples of ‘1-4’ + ‘9-12’ +’17-20’ + 25 | | | |
| **D** | Roots treated with three abiotic stresses | | | | Samples of ‘5-8’ + ‘13-16’ +’21-24’ + 26 | | | |
| **E** | ‘Leaves+Roots’ treated with three abiotic stresses | | | | Samples of 1-26 | | | |
