# Supplementary material for: Selection of reference genes for normalization of cranberry (Vaccinium macrocarpon Ait.) gene expression under different experimental conditions
Source: PLoS One. 2019 Nov 12;14(11):e0224798. doi: 10.1371/journal.pone.0224798 (PMC6850891; doi:10.1371/journal.pone.0224798)
Supplement: S4 Table — (DOC) [file pone.0224798.s007.doc]

**Table S4.** NormFinder Analysis

**No.1** NormFinder analysis of ten candidate reference genes for normalization in different cultivars samples.

| **Gene name** | **Stability value** |  |  |  |
| --- | --- | --- | --- | --- |
| ***F-box*** | 0.386 |  |  |  |
| ***EF-1a*** | 0.200 |  |  |  |
| ***TUBB*** | 0.417 |  |  |  |
| ***PP2A*** | 0.132 |  |  |  |
| ***ACTIN*** | 0.200 |  | **Best gene** | *RH 8* |
| ***CYP2*** | 0.340 |  |  |  |
| ***GAPDH*** | 0.136 |  |  |  |
| ***18s rRNA*** | 0.322 |  |  |  |
| ***RH 8*** | 0.105 |  |  |  |
| ***SAND*** | 0.388 |  |  |  |

**No.2** NormFinder analysis of ten candidate reference genes for normalization in different organs samples.

| **Gene name** | **Stability value** |  |  |  |
| --- | --- | --- | --- | --- |
| ***F-box*** | 0.559 |  |  |  |
| ***EF-1a*** | 0.571 |  |  |  |
| ***TUBB*** | 0.954 |  |  |  |
| ***PP2A*** | 0.228 |  |  |  |
| ***ACTIN*** | 0.651 |  | **Best gene** | *PP2A* |
| ***CYP2*** | 0.342 |  |  |  |
| ***GAPDH*** | 0.389 |  |  |  |
| ***18s rRNA*** | 0.507 |  |  |  |
| ***RH 8*** | 0.550 |  |  |  |
| ***SAND*** | 0.473 |  |  |  |

**No.3** NormFinder analysis of ten candidate reference genes for normalization in leaves samples treated by three abiotic stresses.

| **Gene name** | **Stability value** |  |  |  |
| --- | --- | --- | --- | --- |
| ***F-box*** | 0.394 |  |  |  |
| ***EF-1a*** | 0.564 |  | **Best gene** | 18s rRNA |
| ***TUBB*** | 0.307 |  | **Stability value** | 0.287 |
| ***PP2A*** | 0.324 |  |  |  |
| ***ACTIN*** | 0.405 |  | **Best combination of two genes** | PP2A and CYP2 |
| ***CYP2*** | 0.326 |  | **Stability value for best combination of two genes** | 0.190 |
| ***GAPDH*** | 0.323 |  |  |  |
| ***18s rRNA*** | 0.287 |  |  |  |
| ***RH 8*** | 0.374 |  |  |  |
| ***SAND*** | 0.287 |  |  |  |

| **Intragroup variation** | |  |  |  | **Intergroup variation** | | |  |
| --- | --- | --- | --- | --- | --- | --- | --- | --- |
| **Group identifier** | **1** | **2** | **3** |  | **Group identifier** | **1** | **2** | **3** |
| ***F-box*** | 1.013 | 0.335 | 0.276 |  | ***F-box*** | 0.345 | 0.027 | -0.372 |
| ***EF-1a*** | 1.138 | 1.218 | 1.572 |  | ***EF-1a*** | -0.593 | 0.185 | 0.408 |
| ***TUBB*** | 0.529 | 0.199 | 0.405 |  | ***TUBB*** | 0.045 | -0.007 | -0.038 |
| ***PP2A*** | 0.385 | 0.191 | 0.027 |  | ***PP2A*** | 0.296 | -0.427 | 0.131 |
| ***ACTIN*** | 0.806 | 0.451 | 0.608 |  | ***ACTIN*** | 0.288 | -0.094 | -0.193 |
| ***CYP2*** | 0.048 | 0.289 | 0.105 |  | ***CYP2*** | -0.298 | 0.425 | -0.127 |
| ***GAPDH*** | 0.378 | 0.048 | 0.251 |  | ***GAPDH*** | -0.426 | 0.114 | 0.312 |
| ***18s rRNA*** | 0.285 | 0.250 | 0.271 |  | ***18s rRNA*** | -0.114 | 0.071 | 0.043 |
| ***RH 8*** | 0.644 | 0.380 | 0.481 |  | ***RH 8*** | 0.244 | -0.210 | -0.035 |
| ***SAND*** | 0.305 | 0.161 | 0.182 |  | ***SAND*** | 0.213 | -0.084 | -0.129 |

**No.4** NormFinder analysis of ten candidate reference genes for normalization in roots samples treated by three abiotic stresses.

| **Gene name** | **Stability value** |  |  |  |
| --- | --- | --- | --- | --- |
| ***F-box*** | 0.409 |  |  |  |
| ***EF-1a*** | 0.258 |  | **Best gene** | *RH 8* |
| ***TUBB*** | 0.379 |  | **Stability value** | 0.192 |
| ***PP2A*** | 0.347 |  |  |  |
| ***ACTIN*** | 0.276 |  | **Best combination of two genes** | *EF-1a* and *RH 8* |
| ***CYP2*** | 0.279 |  | **Stability value for best combination of two genes** | 0.107 |
| ***GAPDH*** | 0.223 |  |  |  |
| ***18s rRNA*** | 0.389 |  |  |  |
| ***RH 8*** | 0.192 |  |  |  |
| ***SAND*** | 0.234 |  |  |  |

| **Intragroup variation** | | |  |  | **Intergroup variation** | | |  |
| --- | --- | --- | --- | --- | --- | --- | --- | --- |
| **Group identifier** | **1** | **2** | **3** |  | **Group identifier** | **1** | **2** | **3** |
| ***F-box*** | 0.773 | 0.691 | 0.204 |  | ***F-box*** | 0.166 | 0.227 | -0.393 |
| ***EF-1a*** | 0.126 | 0.139 | 0.002 |  | ***EF-1a*** | 0.215 | -0.333 | 0.118 |
| ***TUBB*** | 0.252 | 0.712 | 0.578 |  | ***TUBB*** | -0.274 | 0.221 | 0.053 |
| ***PP2A*** | 0.057 | 0.007 | 0.262 |  | ***PP2A*** | 0.025 | -0.478 | 0.453 |
| ***ACTIN*** | 0.395 | 0.214 | 0.260 |  | ***ACTIN*** | -0.044 | 0.014 | 0.029 |
| ***CYP2*** | 0.137 | 0.027 | 0.222 |  | ***CYP2*** | 0.221 | 0.120 | -0.340 |
| ***GAPDH*** | 0.579 | 0.020 | 0.054 |  | ***GAPDH*** | 0.076 | -0.082 | 0.006 |
| ***18s rRNA*** | 0.727 | 0.609 | 0.475 |  | ***18s rRNA*** | -0.106 | -0.068 | 0.174 |
| ***RH 8*** | 0.125 | 0.008 | 0.002 |  | ***RH 8*** | -0.103 | 0.187 | -0.084 |
| ***SAND*** | 0.100 | 0.102 | 0.077 |  | ***SAND*** | -0.176 | 0.193 | -0.017 |

**No.5** NormFinder analysis of ten candidate reference genes for normalization in ‘leaves + roots’ samples treated by three abiotic stresses.

| **Gene name** | **Stability value** |  |  |  |  |  |
| --- | --- | --- | --- | --- | --- | --- |
| ***F-box*** | 0.700 |  |  |  |  |  |
| ***EF-1a*** | 0.590 |  |  | **Best gene** | *CYP2* |  |
| ***TUBB*** | 0.446 |  |  | **Stability value** | 0.392 |  |
| ***PP2A*** | 0.450 |  |  |  |  |  |
| ***ACTIN*** | 0.430 |  |  | **Best combination of two genes** | *PP2A* and *CYP2* |  |
| ***CYP2*** | 0.392 |  |  | **Stability value for best combination of two genes** | 0.213 |  |
| ***GAPDH*** | 0.743 |  |  |  |  |  |
| ***18s rRNA*** | 0.425 |  |  |  |  |  |
| ***RH 8*** | 0.514 |  |  |  |  |  |
| ***SAND*** | 0.396 |  |  |  |  |  |

| **Intragroup variation** | | |  |  |  |  |  | **Intergroup variation** | | |  |  |  |  |
| --- | --- | --- | --- | --- | --- | --- | --- | --- | --- | --- | --- | --- | --- | --- |
| **Group identifier** | **1** | **2** | **3** | **4** | **5** | **6** |  | **Group identifier** | **1** | **2** | **3** | **4** | **5** | **6** |
| ***F-box*** | 1.013 | 0.335 | 0.276 | 0.773 | 0.691 | 0.204 |  | ***F-box*** | -0.179 | -0.497 | -0.897 | 0.690 | 0.751 | 0.132 |
| ***EF-1a*** | 1.138 | 1.218 | 1.572 | 0.126 | 0.139 | 0.002 |  | ***EF-1a*** | -0.282 | 0.496 | 0.719 | -0.096 | -0.644 | -0.194 |
| ***TUBB*** | 0.529 | 0.199 | 0.405 | 0.252 | 0.712 | 0.578 |  | ***TUBB*** | 0.115 | 0.063 | 0.031 | -0.343 | 0.152 | -0.017 |
| ***PP2A*** | 0.385 | 0.191 | 0.027 | 0.057 | 0.007 | 0.262 |  | ***PP2A*** | 0.147 | -0.577 | -0.019 | 0.175 | -0.329 | 0.603 |
| ***ACTIN*** | 0.806 | 0.451 | 0.608 | 0.395 | 0.214 | 0.260 |  | ***ACTIN*** | 0.284 | -0.099 | -0.197 | -0.040 | 0.018 | 0.034 |
| ***CYP2*** | 0.048 | 0.289 | 0.105 | 0.137 | 0.027 | 0.222 |  | ***CYP2*** | -0.092 | 0.631 | 0.079 | 0.015 | -0.086 | -0.546 |
| ***GAPDH*** | 0.378 | 0.048 | 0.251 | 0.579 | 0.020 | 0.054 |  | ***GAPDH*** | 0.192 | 0.731 | 0.930 | -0.542 | -0.700 | -0.612 |
| ***18s rRNA*** | 0.285 | 0.250 | 0.271 | 0.727 | 0.609 | 0.475 |  | ***18s rRNA*** | -0.105 | 0.080 | 0.051 | -0.115 | -0.077 | 0.166 |
| ***RH 8*** | 0.644 | 0.380 | 0.481 | 0.125 | 0.008 | 0.002 |  | ***RH 8*** | -0.098 | -0.552 | -0.377 | 0.239 | 0.529 | 0.259 |
| ***SAND*** | 0.305 | 0.161 | 0.182 | 0.100 | 0.102 | 0.077 |  | ***SAND*** | 0.020 | -0.277 | -0.322 | 0.017 | 0.386 | 0.176 |
